# KRAS/ABHD17C/ALOX15B Axis Promotes Pancreatic Cancer Progression via Ferroptosis Evasion

**DOI:** 10.1002/advs.202504470

**Published:** 2025-06-26

**Authors:** Man Li, Xuexin Yu, Yuanji Liu, Shuqin Ouyang, Long Wu, Xiaohong Chen, Huiqi Yu, Haoming Chen, Senmao Lian, Ziwen Li, Liyun Gong, Libing Song, Jun Li

**Affiliations:** ^1^ State Key Laboratory of Oncology in South China Collaborative Innovation Center for Cancer Medicine Sun Yat‐sen University Cancer Center Guangzhou 510080 China; ^2^ Department of Biochemistry Zhongshan School of Medicine Sun Yat‐sen University Guangzhou 510080 China; ^3^ School of Pharmaceutical Sciences Sun Yat‐sen University Guangzhou 510080 China; ^4^ Department of Biochemistry and Molecular Biology Zhongshan School of Medicine Sun Yat‐sen University Guangzhou 510080 China; ^5^ Guangdong Provincial Key Laboratory for Genome Stability and Disease Prevention Department of Biochemistry and Molecular Biology School of Basic Medical Sciences Health Science Center Shenzhen University Shenzhen 518061 China

**Keywords:** KRAS mutation, ferroptosis, pancreatic ductal adenocarcinoma, ALOX15B, ABHD17C

## Abstract

Understanding the mechanisms underlying Kirsten rat sarcoma (KRAS) mutation‐driven development and progression of pancreatic ductal adenocarcinoma (PDAC) may facilitate the discovery of novel strategies for KRAS‐mutant PDAC (KRAS^mut^‐PDAC) treatment. Here, it is reported that downregulation of arachidonate 15‐lipoxygenase (ALOX15B) significantly correlated with poor outcomes in patients with KRAS^mut^‐PDAC. Mechanistically, KRAS^mut^/ERK1‐elicited phosphorylation of ABHD17C promotes depalmitoylation and membrane‐to–cytoplasm translocation of ALOX15B, facilitating proteasome‐dependent degradation of ALOX15B via interaction with the E3 ligase complex CUL4/DDB1/DCAF10. Notably, treatment with methyl protodioscin (MPD), a steroid saponin primarily purified from *polygonatum sibiricum* rhizome, restored the S‐palmitoylation and membrane location of ALOX15B via disruption of the ABHD17C/ALOX15B interaction, consequently resulting in significant inhibition of growth rate of patient‐derived KRAS^mut^‐PDAC organoids in vitro and KRAS^mut^‐PDAC‐formed tumor in vivo via induction of ferroptosis. Therefore, these findings unveil a prominent role of ferroptosis evasion in KRAS^mut^‐PDAC progression and highlight the potential of targeting KRAS/ERK1/ABHD17C/ALOX15B axis in KRAS^mut^‐PDAC treatment.

## Graphical abstract

Phosphorylation of ABHD17C induced by KRAS^mut^/ERK signaling facilitates ALOX15B depalmitoylation and degradation via the CUL4/DDB1/DCAF10 complex, thereby enabling KRAS^mut^‐PDAC cell to evade ferroptosis. Methyl protodioscin inhibits KRAS^mut^‐PDAC progression by disrupting the ABHD17C/ALOX15B interaction and inducing ferroptosis, which represents a potential therapeutic strategy for KRAS^mut^‐PDAC.

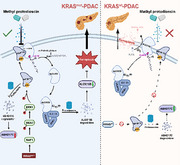

## 1 Introduction

Pancreatic ductal adenocarcinoma (PDAC), accounting for > 90% of all pancreatic malignancies, is a highly devastating gastrointestinal disorder with an extremely poor prognosis:^[^ ^1^, ^2^ ^]^ a 1‐year survival rate for all PDAC stages of ≈18% and a 5‐year overall survival (OS) rate of < 8%. In 86–90% of PDAC cases, tumors harbor activating mutations in the oncogene Kirsten rat sarcoma (*KRAS*) at codon 12, including G12D (45%), G12V (35%), G12R (17%), and G12C (1%–2%).^[^ ^2^, ^3^, ^4^ ^]^ Studies have reported that KRAS mutations lead to an inactive GDP‐binding state in cancer. Moreover, clinical trials have confirmed the anticancer efficacy of KRAS inhibitors, such as MRTX1133, sotorasib, and adagasib. However, rapid tumor resistance development has limited the long‐term effectiveness of these KRAS inhibitors in patients with cancer considerably.^[^ ^5^, ^6^ ^]^ KRAS mutations activate multiple carcinogenic signaling pathways and alter several intracellular metabolic pathways along with increased reactive oxygen species (ROS), exacerbating iron‐dependent lipid peroxidation (LPO), the central mediator of ferroptosis.^[^ ^7^, ^8^ ^]^ Numerous studies have recently highlighted targeting ferroptosis as a new potential strategy for cancer treatment.^[^ ^9^, ^10^, ^11^, ^12^ ^]^ Therefore, developing effective strategies targeting ferroptosis for KRAS‐mutant PDAC (KRAS^mut^‐PDAC) treatment is warranted.

Ferroptosis, a nonapoptotic form of cell death, occurs via iron‐dependent LPO‐induced membrane damage.^[^ ^10^, ^13^, ^14^ ^]^ LPO is driven by multiple iron‐containing enzymes, such as arachidonate lipoxygenases (ALOXs), that have been demonstrated to play a crucial role in ferroptosis through the introduction of hydroperoxy groups (─OOH) into polyunsaturated fatty acids (PUFAs) and membrane phospholipids (PLs).^[^ ^15^, ^16^, ^17^ ^]^ Six ALOX subtypes have been identified in the human genome: ALOX5, ALOX12, ALOX12B, ALOX15, ALOX15B, and ALOXE3. As a crucial arachidonate lipoxygenase, ALOX15B induces ferroptosis through the high‐efficiency insertion of oxygen at carbon atom 15 (C15) of arachidonic acid (AA) to generate pro‐ferroptotic 15S‐hydroperoxyeicosatetraenoic acid (15‐HPETE) death signals.^[^ ^18^, ^19^, ^20^ ^]^ As such, ALOX15B expression has been found to be downregulated in multiple types of cancer, such as oesophageal, breast, prostate, bladder cancer, and non–non‐small‐cell lung cancer (NSCLC),^[^ ^21^, ^22^, ^23^, ^24^, ^25^ ^]^ and restoration of ALOX15B expression has been noted to significantly inhibit cancer progression.^[^ ^21^, ^22^, ^23^, ^24^, ^25^ ^]^ Therefore, understanding precise molecular mechanisms underlying ALOX15B downregulation in cancers may aid in developing novel therapeutic strategies for cancer.

In the present study, we found that ALOX15B overexpression significantly inhibited the growth rate of patient‐derived KRAS^mut^‐PDAC organoids in vitro and KRAS^mut^‐PDAC tumors in vivo. Moreover, we demonstrated that KRAS^mut^/ERK1/ABHD17C‐mediated ALOX15B depalmitoylation elicited the proteasomal degradation of ALOX15B via the CUL4/DDB1/DCAF10 E3 ligase complex. Treatment with methyl protodioscin (MPD) was noted to disrupt ABHD17C–ALOX15B interactions, upregulated ALOX15B expression, and inhibited KRAS^mut^‐PDAC progression in a ferroptosis‐dependent manner. Taken together, our results indicate the plausible mechanism underlying KRAS^mut^/ABHD17C/ALOX15B axis–mediated PDAC progression and ALOX15B restoration, targeting which may be a novel strategy for KRAS^mut^‐PDAC treatment.

## 2 Results

### 2.1 ALOX15B Downregulation Promotes KRAS^mut^‐PDAC Tumour Growth

**Figure 1:**
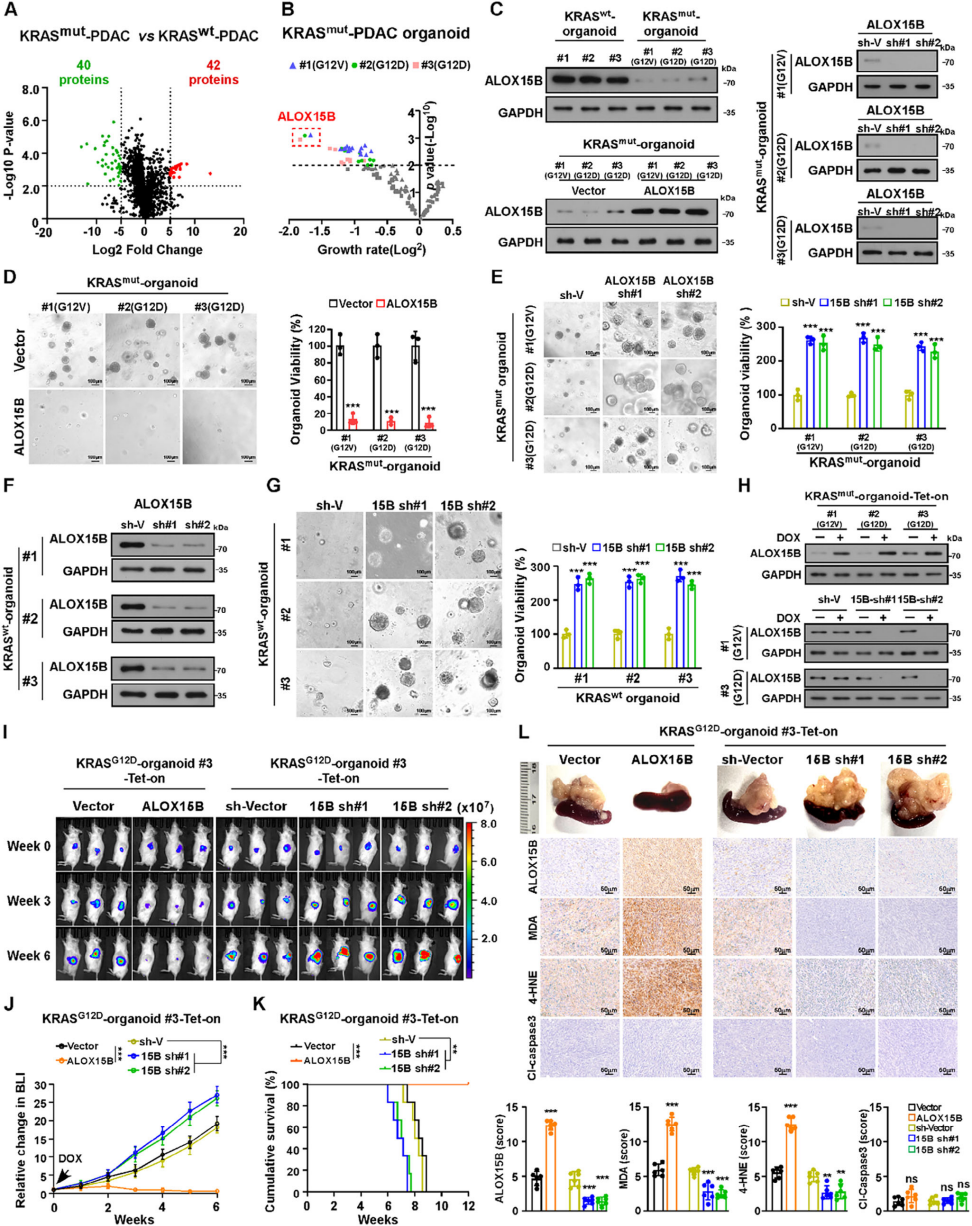
ALOX15B downregulation promotes KRAS^mut^‐PDAC tumour growth. A) Volcano plot of dysregulated proteins comparing KRAS^mut^‐PDAC and KRAS^wt^‐PDAC. B) Organoid viability analysis of inhibitory effects of individually overexpressed 42 downregulated proteins in Figure 1A on growth in 3 KRAS^mut^‐PDAC organoids. C) IB analysis of ALOX15B expression in KRAS^mut^‐PDAC and KRAS^wt^‐PDAC organoids (top left) or in ALOX15B‐overexpressing (bottom left) or ALOX15B‐silenced (right) KRAS^mut^‐PDAC organoids. GAPDH was used as the loading control. D) Representative images (left) and quantification (right) of vector‐ or ALOX15B‐transduced KRAS^mut^‐PDAC organoids. Scale bar: 100 µm. E) Representative images (left) and quantification (right) of sh‐vector‐ or *ALOX15B* shRNA‐transduced KRAS^mut^‐PDAC organoids. Scale bar: 100 µm. F) IB analysis of ALOX15B expression in the indicated KRAS^wt^‐PDAC organoids. GAPDH was used as the loading control. G) Representative images (left) and quantification (right) of sh‐vector‐ or *ALOX15B* shRNA‐transduced KRAS^wt^‐PDAC organoids. Scale bar: 100 µm. H) IB analysis of ALOX15B expression in indicated KRAS^mut^‐PDAC organoids treated with or without DOX. GAPDH was used as the loading control. I) Representative images of tumour‐bearing NOG mice orthotopically inoculated with indicated organoids and treated with DOX at indicated timepoints. J) Relative changes in BLI signal intensities of pancreatic tumours formed by KRAS^G12D^‐PDAC#3 organoids in NOG mice in response to DOX treatment. n = 6 mice per group. K) Kaplan–Meier survival curves of indicated mice. n = 6 mice per group. L) Representative IHC staining images (top) and quantification (bottom) of ALOX15B, MDA, 4‐HNE, and cleaved caspase‐3 staining in KRAS^G12D^‐PDAC#3 organoid xenograft tumours with indicated treatments (top). n = 6 mice per group. Data are the mean ± SD of n=3(A‐B, D‐E, G) and n = 6 (J‐L) biologically independent samples (^**^ *p* < 0.01, ^***^ *p* < 0.001, ns, not significant.). Statistical analysis was performed using an unpaired two‐tailed Student's t‐test (A, B), a two‐way ANOVA, followed by Tukey's test (D, E, G, L), or using a one‐way analysis of variance (ANOVA), followed by Dunnett's test (J, K).

To identify the crucial factors involved in KRAS^mut^‐PDAC progression, the label‐free quantitative proteomics (LFQP) was performed to analyze the differential protein abundance in three KRAS^mut^ tissue types: 2 KRAS^G12D^‐PDAC, 1 KRAS^G12V^‐PDAC, and 3 KRAS wild‐type (KRAS^wt^)‐PDAC tissues. LFQP analysis revealed that a total of 82 proteins were significantly dysregulated, including 40 downregulated and 42 upregulated proteins (*p* < 0.01, |log_2_fold change| > 5), in KRAS^mut^‐PDAC tissues compared with KRAS^wt^‐PDAC tissues (**Figure** **1**A). We then examined the effects of the 40 downregulated proteins on the growth of PDAC organoids generated from 3 KRAS^mut^‐PDAC and 3 KRAS^wt^‐PDAC tissues. As shown in Figure 1B–D, among all 40 downregulated proteins, ALOX15B overexpression had the most significant inhibitory effect on the growth of all three KRAS^mut^‐PDAC organoids. In contrast, silencing ALOX15B significantly increased KRAS^mut^‐PDAC and KRAS^wt^‐PDAC organoid growth rates (Figure 1E–G). Thus, the downregulation of ALOX15B protein may be linked with KRAS^mut^‐PDAC progression.

Furthermore, RNA‐sequencing (RNA‐seq) analysis was performed on 3 KRAS^mut^‐PDAC and KRAS^wt^‐PDAC tissues, which revealed that a total of 281 genes, including 123 downregulated genes and 158 upregulated genes (*p* < 0.05, |log_2_fold change| > 1), were dysregulated in the KRAS^mut^‐PDAC tissues compared to the KRAS^wt^‐PDAC tissues (Figure S1A, Supporting Information). Interestingly, KRAS^mut^‐PDAC tissues exhibited higher mRNA levels of ALOX15B compared to KRAS^wt^‐PDAC tissues (Figure S1A, Supporting Information). This result was further confirmed by Real‐time PCR analysis, in which the mRNA level of ALOX15B in KRAS^mut^‐PDAC cells was significantly higher than that in KRAS^wt^‐PDAC cells (Figure S1B, Supporting Information). Therefore, these results suggested that the downregulation of ALOX15B protein in KRAS^mut^‐PDAC might occur through post‐transcriptional regulation.

Next, we established doxycycline (DOX)‐inducible ALOX15B‐overexpressing and silenced KRAS^mut^‐PDAC organoids and orthotopically xenografted them into the pancreas of NOG mice (Figure 1H). DOX was intraperitoneally injected when the bioluminescence imaging (BLI) signal intensity became 3.0 × 10^6^ p/s/cm^2^/sr. As illustrated in Figure 1G–I and Figure S1C (Supporting Information), the burden of tumors formed by ALOX15B transduced‐KRAS^mut^‐PDAC organoids was significantly lower than that of tumors formed by control organoids, leading to longer ALOX15B/KRAS^mut^‐PDAC organoid‐bearing mice. However, mice implanted with ALOX15B‐silenced‐KRAS^mut^‐PDAC organoids had significantly higher tumor growth rates and shorter survival (Figure 1K,J and Figure S1D, Supporting Information). Therefore, these results further support the notion that ALOX15B downregulation is involved in KRAS^mut^‐PDAC progression.

As an arachidonate 15‐lipoxygenase, ALOX15B specifically catalyzes the dioxygenation of arachidonic acid (AA) at carbon 15 (C15) when esterified to membrane phospholipids or cholesterol esters, generating the pro‐ferroptotic lipid mediator 15S‐hydroperoxyeicosatetraenoic acid (15‐HPETE).^[^ ^18^, ^19^, ^20^ ^]^ Consistent with its metabolic function, pharmacological inhibition of ALOX15B confers significant protection against ferroptosis, while its genetic restoration potently triggers ferroptotic cell death.^[^ ^15^, ^26^, ^27^ ^]^ Based on these mechanistic insights into ALOX15B‐dependent ferroptosis regulation, we propose that ALOX15B downregulation facilitates KRAS^mut^‐PDAC progression by attenuating ferroptotic signaling. Compared with tumors formed by control organoids, the levels of 4‐hydroxynonenal (4‐HNE) and malondialdehyde (MDA), two widely used indicators of LPO,^[^ ^28^ ^]^ were significantly lower in tumors formed by ALOX15B silenced‐KRAS^mut^‐PDAC organoids but significantly higher in tumors formed by ALOX15B/KRAS^mut^‐PDAC organoids. However, no alterations were noted in the levels of the apoptotic marker cleaved caspase‐3 in these tumors (Figure 1L; Figure S1E, Supporting Information). Therefore, ALOX15B downregulation–mediated KRAS^mut^‐PDAC progression might occur through ferroptosis inhibition.

### 2.2 ALOX15B Downregulation is Correlated with Poor KRAS^mut^‐PDAC Prognosis

**Figure 2:**
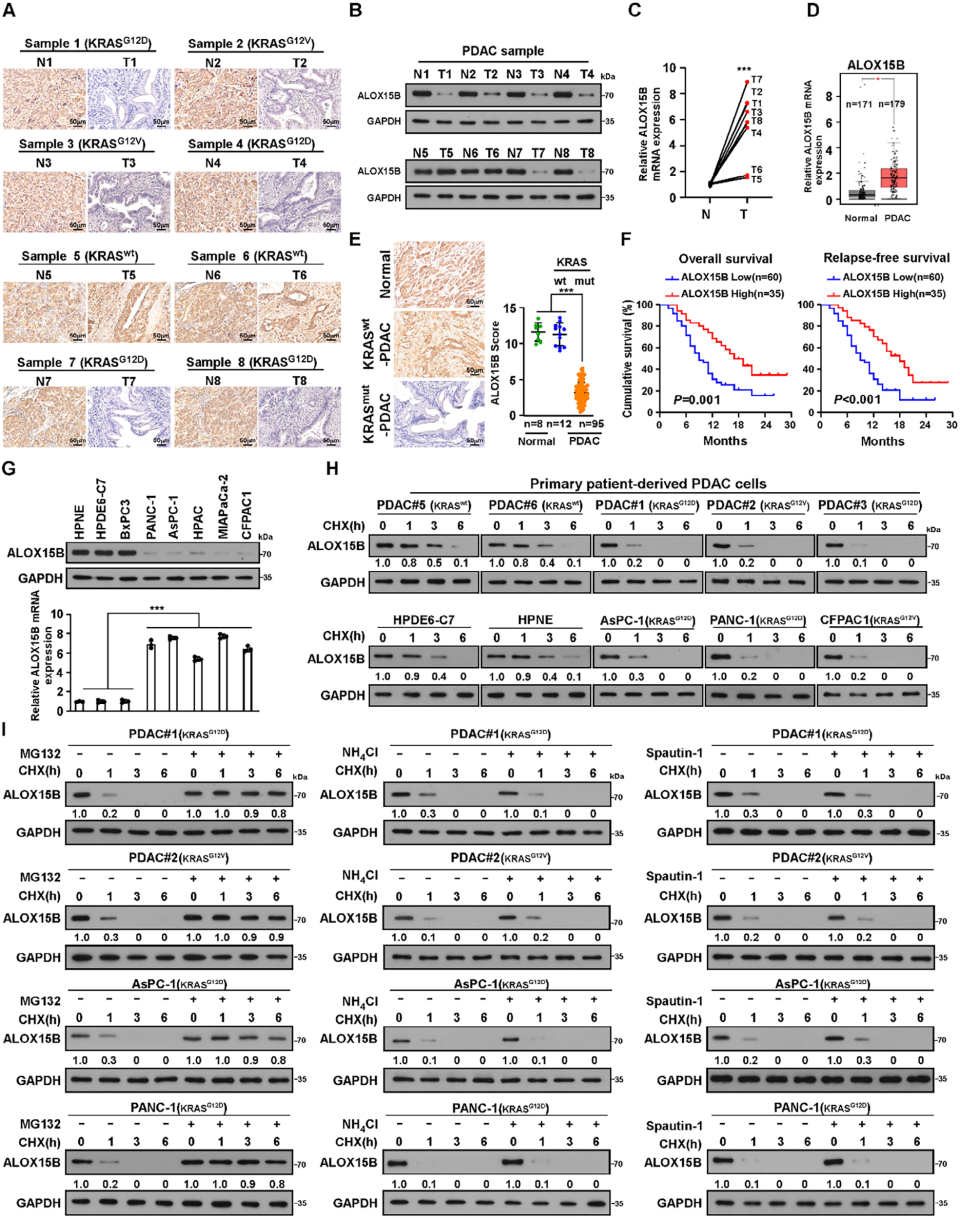
ALOX15B is downregulated in KRAS^mut^‐PDAC via proteasome‐mediated degradation. A,B) IHC staining and IB analyses of ALOX15B expression in 8 PDAC tissue samples, including 4 KRAS^G12D^‐PDAC, 2 KRAS^G12V^‐PDAC, and 2 KRAS^wt^‐PDAC tissue samples, and their paired adjacent normal pancreatic tissue samples. GAPDH was used as the loading control. C) Quantitative PCR analysis of *ALOX15B* mRNA level in the 8 paired PDAC and adjacent normal pancreatic tissue samples. *GAPDH* was used as the loading control. D) Analysis of The Cancer Genome Atlas dataset showing that *ALOX15B* mRNA expression in PDAC tissues (n = 179) was significantly higher than that in normal pancreatic tissues (n = 171). E) Representative IHC staining images (left) and quantification (right) of ALOX15B expression in normal pancreatic (n = 8), KRAS^wt^‐PDAC (n = 12) and KRAS^mut^‐PDAC (n = 95) tissues. Scale bar: 50 µm. F) Kaplan–Meier analysis of OS (left) and RFS (right) curves in patients with KRAS^mut^‐PDAC with low versus high ALOX15B expression. G) IB (top) and quantitative PCR (bottom) analyses of ALOX15B expression in the indicated cells. GAPDH was used as the loading control. H) CHX (cycloheximide, 100 µg mL^−1^) chase analysis of ALOX15B half‐life at indicated timepoints. GAPDH was used as the loading control. Protein quantified against controls, set as 1.0. I) CHX chase analysis of ALOX15B half‐life in indicated cells treated with CHX (100 µg mL^−1^) plus MG132 (10 µm), or NH_4_Cl (20 mm), or spautin‐1(5 µm), at the indicated timepoints. GAPDH was used as the loading control. Protein quantified against controls, set as 1.0. Each error bar in e and g represents the mean ± SD of 3 independent experiments (^***^ *p* < 0.001). Statistical analysis was performed using one‐tail Mann‐Whitney U test (C), an unpaired two‐tailed Student's t‐test (D,E), a one‐way ANOVA, followed by Dunnett's test (F), or a two‐way ANOVA, followed by Tukey's test (G).

Consistent with the LFQP results (Figure 1A), immunohistochemistry (IHC) and immunoblotting (IB) analyses revealed that compared with their paired adjacent normal pancreatic tissues, the expression of ALOX15B protein was much lower in KRAS^mut^‐PDAC tissues, including 4 KRAS^G12D^‐ and 2 KRAS^G12V^‐PDAC, but was nearly same in 2 KRAS^wt^‐PDAC tissues (**Figure** **2**A,B; Figure S2A–H, Supporting Information). However, *ALOX15B* mRNA expression was significantly higher in KRAS^mut^‐PDAC tissues than in their paired adjacent normal pancreatic tissues and KRAS^wt^‐PDAC tissues (Figure 2C), which was further confirmed based on the data from the public datasets GEPIA and GSE234927 (Figure 2D; Figure S3A, Supporting Information). Similar results were observed in KRAS^mut^‐PDAC cell lines (Figure 2G).

To determine the clinical relevance of reduced ALOX15B expression in KRAS^mut^‐PDAC, the ALOX15B level was examined in 107 paraffin‐embedded, archived PDAC tissues, including 12 KRAS^wt^‐ and 95 KRAS^mut^‐PDAC using IHC analysis. Compared to 8 tumor‐adjacent normal pancreatic tissues, ALOX15B expression remained higher in KRAS^wt^‐PDAC tissues but was considerably reduced in KRAS^mut^‐PDAC tissues (Figure 2E). Moreover, reduced ALOX15B expression was strongly correlated with the TNM stage and relapse (Tables S1 and S2, Supporting Information). Importantly, patients with ALOX15B‐low expressing KRAS^mut^‐PDAC had shorter overall survival (OS) and relapse‐free survival (RFS) (P = 0.001, *p* < 0.001, respectively; Figure 2F and Tables S1 and S2, Supporting Information). Taken together, these results suggested that ALOX15B downregulation is linked to the clinical progression of KRAS^mut^‐PDAC.

### 2.3 ALOX15B is Downregulated in KRAS^mut^‐PDAC via Proteasome‐Mediated Degradation

To further confirm that the reduced ALOX15B protein in KRAS^mut^‐PDAC is through post‐transcriptional regulation, cycloheximide (CHX) chase assay was performed and showed that the half‐life of ALOX15B was much shorter in patient‐derived primary KRAS^mut^‐PDAC cells than in patient‐derived KRAS^wt^‐PDAC cells, and in KRAS^mut^‐PDAC cell lines than in KRAS^wt^‐PDAC cell lines and normal pancreatic duct epithelial cells (Figure 2H). Moreover, treatment with proteasome inhibitor MG132, but not autophagy inhibitor Spautin‐1 or lysosome inhibitor NH_4_Cl, abolished CHX‐mediated downregulation of ALOX15B in KRAS^mut^‐PDAC cells (Figure 2I). Therefore, ALOX15B downregulation in KRAS^mut^‐PDAC is attributable to proteasome‐dependent degradation.

### 2.4 CUL4/DDB1/DCAF10 E3 Ligase Complex Promotes ALOX15B Degradation

**Figure 3:**
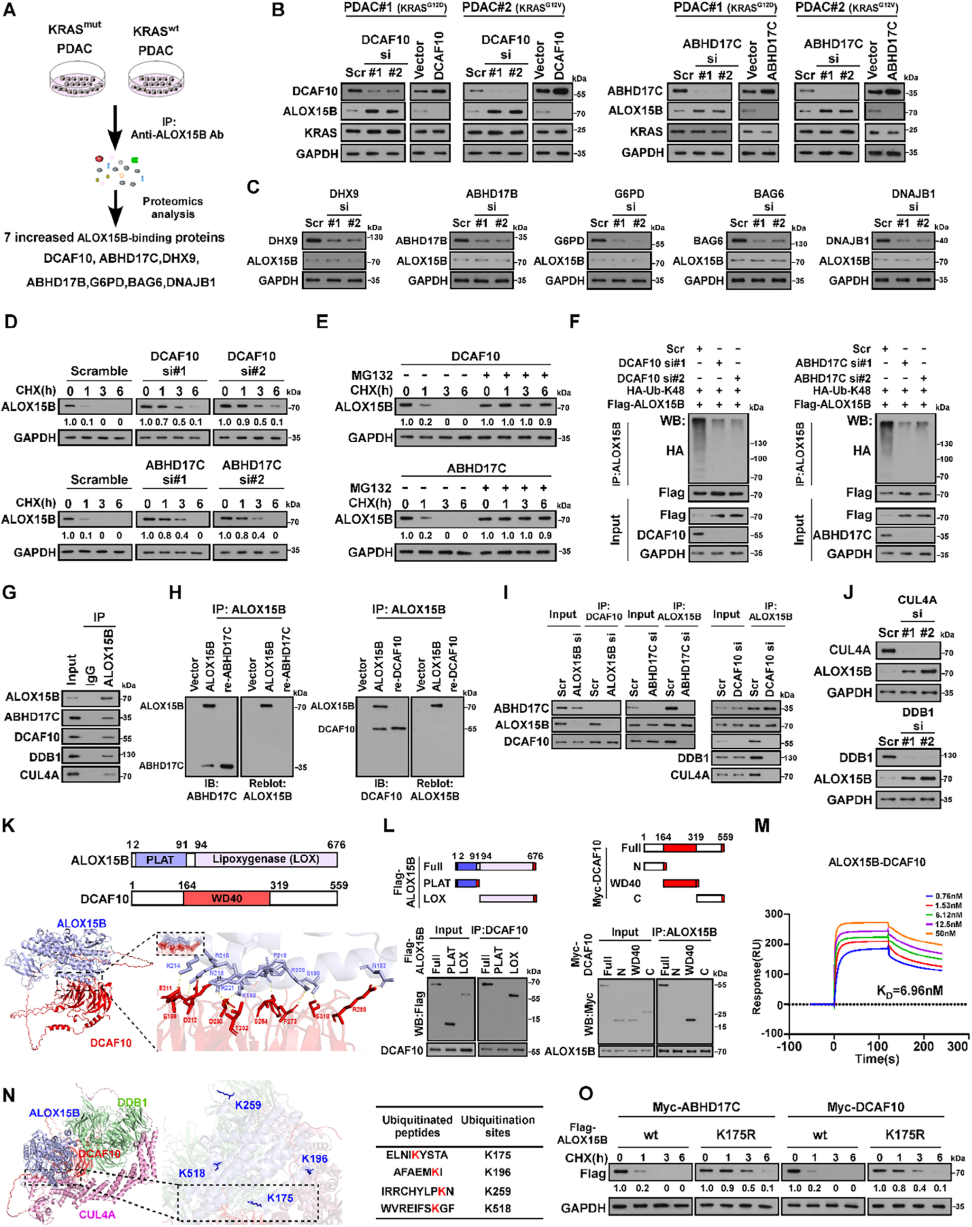
E3 ligase complex CUL4/DDB1/DCAF10 promotes ALOX15B degradation. A) Schematic of experimental design to explore the potential increased ALOX15B‐binding proteins in KRAS^mut^‐PDAC cells compared to KRAS^wt^‐PDAC cells analysed through IP‐MS. B) IB analysis of ALOX15B, DCAF10, ABHD17C, and KRAS expression in the indicated cells. GAPDH was used as the loading control. C) IB analysis of ALOX15B, DHX9, ABHD17B, G6PD, BAG6, and DNAJB1 expression in PDAC#1 (KRAS^G12D^) cells transfected with the corresponding siRNAs. GAPDH was used as the loading control. D) CHX chase analysis of ALOX15B half‐life in DCAF10 or ABHD17C siRNA‐transfected PDAC#1 (KRAS^G12D^) cells at the indicated timepoints. GAPDH was used as the loading control. Protein quantified against controls, set as 1.0. E) CHX chase analysis of ALOX15B half‐life in the DCAF10‐ or ABHD17C‐overexpressing PDAC#5 (KRAS^wt^) cells treated with CHX (100 µg/mL) plus vehicle or MG132 (10 µM) at the indicated timepoints. GAPDH was used as the loading control. Protein quantified against controls, set as 1.0. F) IB analysis of ALOX15B, DCAF10, and HA‐Ub expression and IP/IB analysis of ALOX15B and HA‐Ub‐linked ALOX15B expression in the indicated PDAC#1 (KRAS^G12D^) cells transfected with *DCAF10*‐siRNA (left) or *ABHD17C*‐siRNA (right). **G)** IP/IB analysis of ALOX15B, CUL4A, DDB1, DCAF10, and ABHD17C expression in PDAC#1 (KRAS^G12D^) cells. H) Far‐western blotting analysis was performed using anti‐Flag antibody‐immunoprecipitated proteins and detected using anti‐DCAF10 (left) or anti‐ABHD17C (right) and then reblotted with anti‐ALOX15B. Recombinant DCAF10 and ABHD17C were used as controls. I) IP assay results showing no interaction between ABHD17C and DCAF10. Silencing ABHD17C reduced ALOX15B–DCAF10 interactions (left), whereas downregulating DCAF10 did not affect ALOX15B–ABHD17C interactions (right). J) IB analysis of CUL4A, ALOX15B, and DDB1 expression in *CUL4A* or *DDB1* siRNA‐transfected PDAC#1 (KRAS^G12D^) cells. GAPDH was used as the loading control. K) AlphaFold3 analysis of 3D structure of DCAF10/ALOX15B interaction region. L) Left: Schematic of full‐length and truncated ALOX15B protein structure (top), and IP analysis of interactions of DCAF10 with full‐length and truncated ALOX15B fragments (bottom). Right: Schematic of full‐length and truncated DCAF10 protein structure (top), and IP/IB analysis of interactions of ALOX15B with full‐length or truncated DCAF10 fragments (bottom). M) SPR analysis of direct ALOX15B–DCAF10 interaction ALOX15B was immobilised on a Series S Sensor Chip. The K_D_ value of ALOX15B–DCAF10 interactions was calculated as the raw response (RU). N) AlphaFold3 analysis of the 3D structure of the ALOX15B/DCAF10/DDB1/CUL4A complex (left), GPS‐Uber analysis of the predicted potential ubiquitination sites in ALOX15B (right). O) CHX chase analysis of ALOX15B‐wt and ALOX15B‐K175R half‐lives in ABHD17C‐ or DCAF10‐transfected cells at indicated timepoints. GAPDH was used as the loading control. Protein quantified against controls, set as 1.0.

To clarify the mechanism underlying ALOX15B downregulation in KRAS^mut^‐PDAC, affinity purification following the quantitative proteomics analysis was performed. As shown in **Figure** **3**A, the interaction of ALOX15B with 7 proteins was significantly increased in KRAS^mut^‐PDAC cells compared to KRAS^wt^‐PDAC cells (*p* < 0.01; log_2_fold change > 4). However, only silencing ABHD17C or DCAF10, rather than other proteins, resulted in drastic increased ALOX15B expression and the half‐life of ALOX15B in KRAS^mut^‐PDAC cells; this effect was not observed in KRAS^wt^‐PDAC cells (Figure 3B,C; Figure S3B, Supporting Information). Dysregulation of ABHD17C or DCAF10 did not result in obvious alterations of mRNA levels of ALOX15B and KRAS (Figure S3C, Supporting Information). Meanwhile, IB analysis revealed that the ABHD17C‐ or DCAF10‐transduced cells and ABHD17C‐ or DCAF10‐silenced cells exhibited nearly the same expression of KRAS protein compared with parental cells (Figure 3B; Figure S3B, Supporting Information). However, we found that overexpressing ABHD17C or DCAF10 resulted in dramatically decreased ALOX15B expression and faster ALOX15B degradation, which was drastically abrogated by MG132 treatment (Figure 3B–E). These results suggested that DCAF10‐mediated ALOX15B reduction was through post‐transcriptional mechanism. Consistent with this hypothesis, silencing either DCAF10 or ABHD17C drastically reduced level of K48‐polyubiquitinated ALOX15B, but not the level of K6‐, K11‐, K27‐, K29‐, K33‐, or K63‐polyubiquitinated ALOX15B, in KRAS^mut^‐PDAC cells (Figure 3F; Figure S3D, Supporting Information). Therefore, these results indicated that ABHD17C and DCAF10 contribute to proteasome‐mediated ALOX15B degradation in KRAS^mut^‐PDAC.

Consistent with the proteomics results (Figure 3A), reciprocal co‐immunoprecipitation (co‐IP) and far‐western blotting demonstrated that ALOX15B directly interacted with ABHD17C or DCAF10 (Figure 3G,H). However, silencing ABHD17C dramatically abrogated ALOX15B–DCAF10 interactions, whereas downregulating DCAF10 had no impact on ABHD17C‐binding affinity of ALOX15B (Figure 3I). Therefore, these results suggested that ABHD17C‐mediated ALOX15B modification might be required for ALOX15B/DCAF10 interaction. Previously, it has been reported DCAF10 interacted with DDB1 and CUL4A to form E3 ubiquitin ligase machinery.^[^ ^29^, ^30^ ^]^ Consistently, we found that ALOX15B could form a complex with DDB1 and CUL4A (Figure 3I). However, the downregulation of DCAF10 substantially reduced ALOX15B–DDB1 and ALOX15B–CUL4A binding (Figure 3I). Thus, the ubiquitin ligase complex CUL4/DDB1/DCAF10 may be involved in ALOX15B degradation in KRAS^mut^‐PDAC. In agreement with this hypothesis, silencing either CUL4A or DDB1 resulted in considerable ALOX15B upregulation (Figure 3J).

Analysis using the AlphaFold3 server (https://golgi.sandbox.google.com/) revealed that the residues N183‐R221 at the N‐terminus of ALOX15B automatically dock with the residues E188‐S318 in the central WD40 repeat region of DCAF10 (Figure 3K). This automated prediction of ALOX15B/DCAF10 interaction was further confirmed via co‐IP assays using serially truncated ALOX15B and DCAF10 fragments (Figure 3L). Surface plasmon resonance (SPR) analysis revealed that the K_D_ of ALOX15B/DCAF10 binding was 6.96 nM (Figure 3M). We then analysed the 3D structure of the ALOX15B/DCAF10/DDB1/CUL4A complex using AlphaFold3, which showed that the E3 ligase CUL4A is very close to K175, one of the four potential ubiquitination sites (i.e. K175, K196, K259, and K518), in ALOX15B, as predicted by the GPS‐Uber web server (http://gpsuber.biocuckoo.cn/online.php; Figure 3N). However, only mutation at K175 but not K196, K259, and K518 in ALOX15B abrogated the ALOX15B expression–reducing effects of ABHD17C or DCAF10 overexpression (Figure 3O; Figure S3E, Supporting Information). Therefore, the E3 ligase complex CUL4/DDB1/DCAF10 might be involved in the ubiquitin degradation of ALOX15B.

### 2.5 ABHD17C‐Mediated Depalmitoylation Promotes ALOX15B Degradation

**Figure 4:**
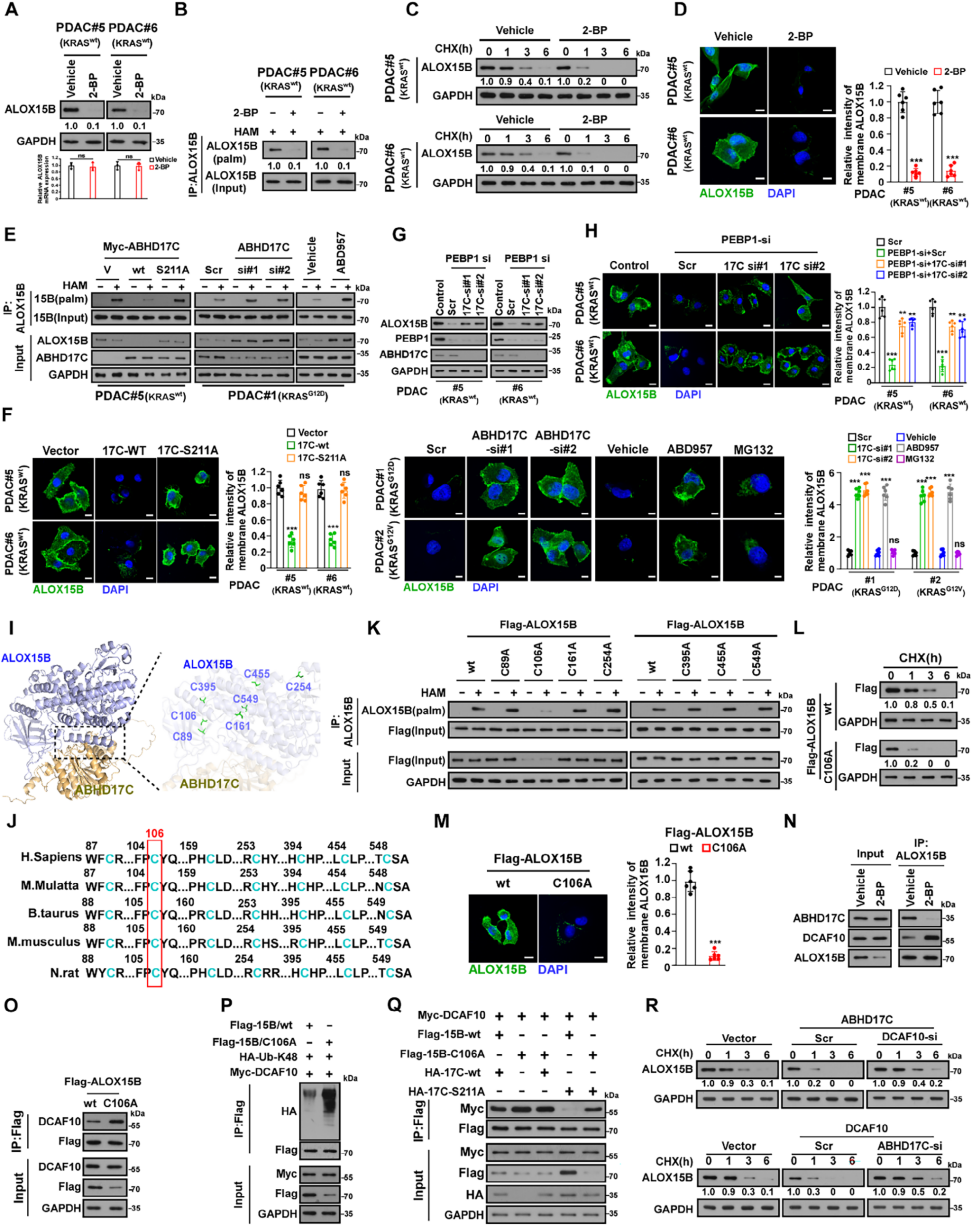
ABHD17C‐mediated depalmitoylation promotes DCAF10‐induced ALOX15B degradation. A) IB analysis of ALOX15B protein expression (upper) and quantitative PCR analysis of ALOX15B mRNA level (lower) in the indicated cells treated with vehicle or 2‐bromopalmitate (2‐BP, 50 µm) for 24 h. B) PDAC#5 (KRAS^wt^) and PDAC#6 (KRAS^wt^) cells were pre‐treated with vehicle or 2‐BP (50 µm) for 24 h, followed by MG132 (10 µm) for 6 h. The precipitated proteins were treated with hydroxylamine (HAM) to remove palmitic acid from palmitoylated cysteine residues. Free cysteines were labeled with HPDP‐Biotin. The immunoprecipitated samples were analysed by immunoblot using anti‐ALOX15B antibody and HRP‐streptavidin. C) IB analysis of ALOX15B expression in the indicated cells pretreated with 2‐BP (50 µm), followed by CHX (100 µg mL^−1^) treatment at indicated timepoints. GAPDH was used as the loading control. Protein quantified against controls, set as 1.0. D) Representative IF images (left) and quantification (right) of membrane ALOX15B in the indicated cells treated with vehicle or 2‐BP (50 µm). Scale bar: 10 µm. E) ABE/IB analysis of expression of palmitoylated ALOX15B in indicated cells. F) Representative IF images (left) and quantification (right) of membrane ALOX15B in the indicated cells. Scale bar: 10 µm. G) IB analysis of ALOX15B, PEBP1, and ABHD17C expression in the indicated cells. GAPDH was used as the loading control. H) Representative IF images (left) and quantification (right) of membrane ALOX15B in the indicated cells. Scale bar: 10 µm. I) AlphaFold3 analysis of 3D structure of ALOX15B/DCAF10 interaction region. J) Alignment of ALOX15B sequences containing potential palmitoylation sites in different species. K) ABE/IB analysis of expression of palmitoylated ALOX15B in cells transfected with indicated ALOX15B mutants. L) CHX chase analysis of half‐life of ALOX15B‐wt or ALOX15B‐C106A mutant at indicated timepoints. GAPDH was used as the loading control. Protein quantified against controls, set as 1.0. M) Representative IF images (left) and quantification (right) of membrane ALOX15B‐wt or ALOX15B‐C106A mutant in indicated cells. Scale bar: 10 µm. N) IP/IB analysis of interactions of ALOX15B with ABHD17C or DCAF10 in the cells treated with vehicle or 2‐BP (50 µm). O) IP/IB analysis of interaction of DCAF10 with ALOX15B‐wt or ALOX15B‐C106A mutant. P) IP/IB analysis of the K48‐linked polyubiquitination of ALOX15B‐wt or ALOX15B‐C106A mutant in the DCAF10‐transfected cells. Q) IP/IB analysis of interactions of DCAF10 with ALOX15B‐wt or ALOX15B‐C106A mutant in ABHD17C‐wt or ABHD17C‐S211A‐transfected cells. R) CHX chase analysis of ALOX15B half‐life in indicated cells. GAPDH was used as the loading control. Protein quantified against controls, set as 1.0. Data are the mean ± SD of n = 6 (D, F, H, M) biologically independent samples (^**^ *p* < 0.01, ^***^ *p* < 0.001, ns, not significant.). Statistical analysis was performed using a two‐way ANOVA, followed by Tukey's test (D, F, H), or an unpaired two‐tailed Student's t‐test (M).

Since silencing the depalmitoylase ABHD17C drastically abrogated the ALOX15B/DCAF10 interactions while downregulating DCAF10 had not affect the ALOX15B/ABHD17C complex formation (Figure 3I), this suggested that ABHD17C‐mediated ALOX15B depalmitoylation might be required for CUL4/DDB1/DCAF10‐induced ALOX15B degradation. To test this hypothesis, we first examined whether palmitoyl modification contributed to ALOX15B stability. As shown in **Figure** **4**A–D, treatment with 2‐bromopalmitate (2‐BP), a widely used palmitoylation inhibitor, in ALOX15B‐high expressing KRAS^wt^‐cells resulted in dramatically reduced total and palmitoylated levels, shorter half‐life, and decreased membrane localization of ALOX15B, but had no obvious impact on the mRNA level of ALOX15B, compared to vehicle‐treated cells. Meanwhile, the 2‐BP‐treated cells exhibited less ALOX15B. Consistent with the effect of 2‐BP treatment on ALOX15B downregulation, overexpressing depalmitoylase ABHD17C, but not depalmitoylase‐dead ABHD17C‐S211A mutant, resulted in drastic ALOX15B reduction in ALOX15B‐high expressing cells. In contrast, silencing ABHD17C or inhibiting ABHD17C activity by ABD957 dramatically increased ALOX15B protein expression in ALOX15B‐low expressing cells (Figure 4E,F). Moreover, IF staining assay showed that ALOX15B expression drastically increased and localized in the cytoplasm in the MG132‐treated cells (Figure 4F). Therefore, these results further confirmed that ABHD17C‐mediated depalmitoylation was involved in ALOX15B degradation. It has been reported that phosphatidylethanolamine‐binding protein 1 (PEBP1) contributes to membrane translocation of ALOX15B via forming complexes with ALOX15B.^[^ ^27^ ^]^ However, we found that further downregulating ABHD17C restored, at least partially, PEBP1 silencing‐reduced membrane localization of ALOX15B (Figure 4G,H). These results provided a new mechanism underlying ALOX15B membrane localization.

AlphaFold3 analysis revealed that the 3D structures of ABHD17C and ALOX15B automatically docked with each other, with the residues D96‐Q147 in the central region of ABHD17C associating with the residues N183‐K612 at the C‐terminus of ALOX15B (Figure 4I). Furthermore, SwissPalm analysis (https://swisspalm.org/) indicated that 7 cysteine residues of ALOX15B, including C89, C106, C161, C254, C395, C455, and C549, are potential palmitoylation sites, which are mostly conserved across different species (Figure 4J). However, only mutation C106 in ALOX15B (ALOX15B‐C106A) drastically reduced the levels of palmitoylated and total ALOX15B (Figure 4K). Similarly, ALOX15B‐C106A mutation‐overexpressing cells exhibited a shorter half‐life and less ALOX15B membrane localization (Figure 4L,M). Notably, co‐IP assays demonstrated that inhibition of ALOX15B palmitoylation with 2‐BP or introducing C106 mutation in ALOX15B reduced ALOX15B/ABHD17C interaction but increased ALOX15B/DCAF10 association (Figure 4N,O), suggesting that ABHD17C‐mediated ALOX15B depalmitoylation promoted DCAF10‐induced ALOX15B degradation. Consistently, ALOX15B‐C106A mutant exhibited increased K48‐linked polyubiquitinated level (Figure 4P), and overexpressing ABHD17C drastically increased the DCAF10/ALOX15B interaction and ALOX15B/26S proteasome co‐localization (Figure 4Q; Figure S4A, Supporting Information). Importantly, silencing DCAF10 abrogated the inductive effect of ABHD17C overexpression on ALOX15B degradation but DCAF10 overexpression did not recover ABHD17C silencing‐induced ALOX15B degradation in the cells (Figure 4R). Taken together, these results indicate that ABHD17C‐mediated ALOX15B depalmitoylation promotes DCAF10‐induced ALOX15B degradation.

### 2.6 Upregulated ABHD17C is Associated with PDAC Progression

**Figure 5:**
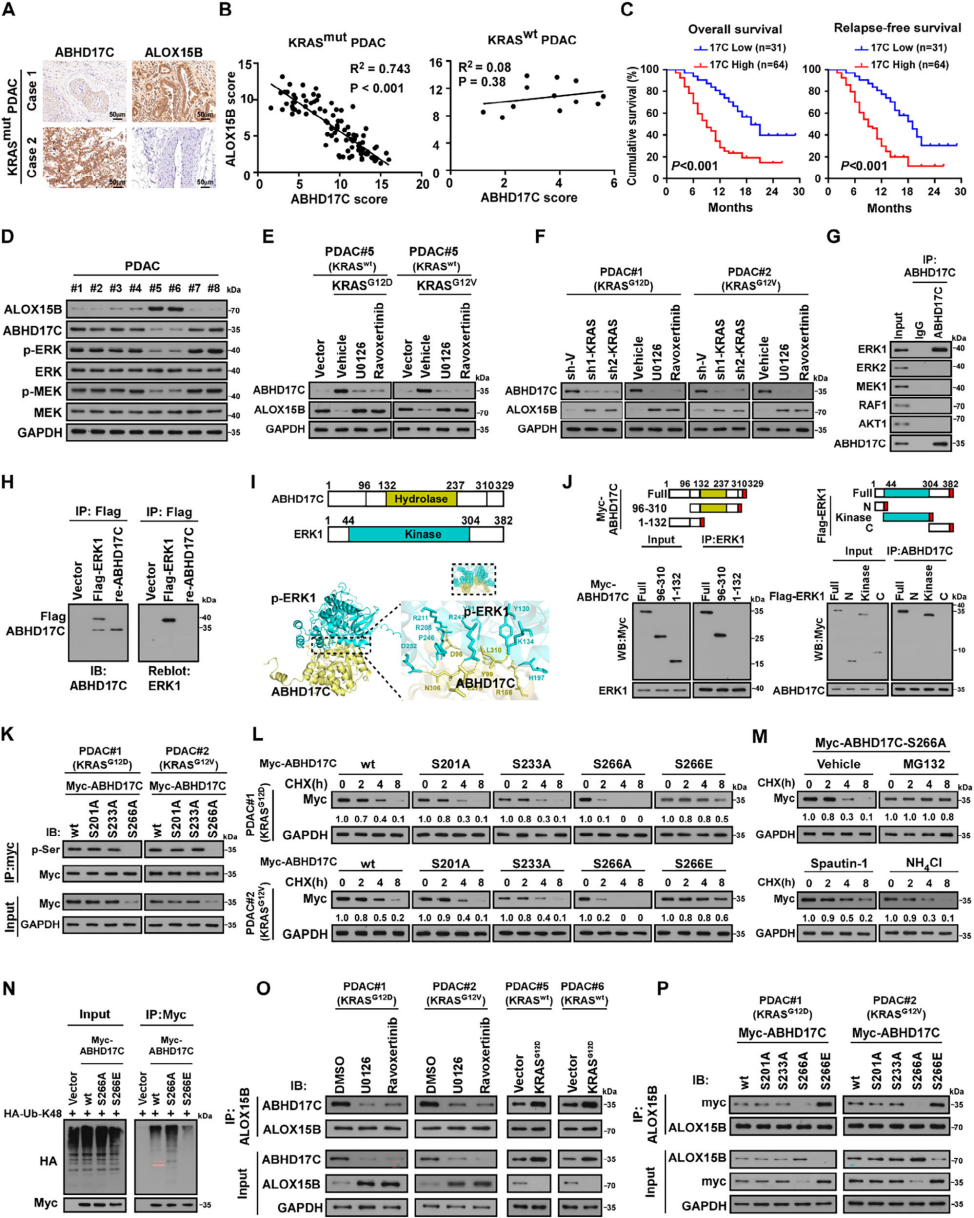
ERK1 phosphorylates and stabilizes ABHD17C. A) Representative IHC staining images of ABHD17C and ALOX15B in KRAS^mut^‐PDAC tissues. Scale bar: 50 µm. B) Statistical analysis of IHC results showing that ALOX15B expression was inversely correlated with ABHD17C level in KRAS^mut^‐PDAC tissues (n = 95; P < 0.001, R^2^ = 0.743) but not in KRAS^wt^‐PDAC tissues (n = 12; P = 0.38, R^2^ = 0.08). C) Kaplan–Meier analysis of OS (left) and RFS (right) curves in patients with KRAS^mut^‐PDAC with low versus high ABHD17C expression. D) IB analysis of ALOX15B, ABHD17C, p‐ERK, total ERK, p‐MEK, and total MEK expression in 8 PDAC tissue samples. GAPDH was used as the loading control. E) IB analysis of ABHD17C and ALOX15B expression in indicated cells transfected with KRAS^G12D^ or KRAS^G12V^ mutant or treated with the MEK inhibitor U0126 (10 µm) or the ERK inhibitor ravoxertinib (0.1 µm). GAPDH was used as the loading control. F) IB analysis of ABHD17C and ALOX15B expression in the KRAS^G12D^ or KRAS^G12V^ ‐mutant‐transduced cells transfected with *KRAS* siRNA or treated with U0126 (10 µM) or ravoxertinib (0.1 µm). GAPDH was used as the loading control. G) IP/IB analysis of interactions of ABHD17C with ERK1, ERK2, MEK1, RAF1 and AKT1. H) Far‐western blotting analysis performed using anti‐Flag antibody‐immunoprecipitated proteins, followed by detection using anti‐ERK1 and then reblotting with anti‐ABHD17C. Recombinant ERK1 was used as the control. I) AlphaFold3 analysis of 3D structure of ABHD17C‐p‐ERK1 interaction region. J) Left: Schematic of full‐length and truncated ABHD17C protein structure (top), and co‐IP assay performed using anti‐ERK1 in cells transfected with full‐length and truncated ABHD17C fragments (bottom). Right: Schematic of full‐length and truncated ERK1 protein structure (top), and co‐IP assay performed using anti‐ABHD17C in cells transfected with full‐length and truncated ERK1 fragments (bottom). K) IP/IB analysis of phosphorylated ABHD17C in ABHD17C‐wt‐ or ABHD17C‐mutant‐transfected cells. L) CHX chase analysis of half‐life of ABHD17C‐wt‐ or ABHD17C‐mutant‐transfected cells at indicated timepoints. GAPDH was used as the loading control. Protein quantified against controls, set as 1.0. M) CHX chase analysis of ABHD17C‐S266A half‐life in cells treated with CHX (100 µg mL^−1^) plus vehicle, or MG132 (10 µM;), or spautin‐1 (5 µm), or NH_4_Cl (20 mm) at indicated timepoints. GAPDH was used as the loading control. Protein quantified against controls, set as 1.0. N) IB analysis of the K48‐linked polyubiquitinated level of ABHD17C‐wt, ABHD17C‐S266A mutant, or ABHD17C‐S266E mutant. O) IP/IB analysis of ALOX15B/ABHD17C interaction in the indicated cells. P) IP/IB analysis of interaction of ALOX15B with ABHD17C‐wt or indicated ABHD17C mutants. Statistical analysis was performed using two‐tailed Spearman`s test (B), or a one‐way ANOVA, followed by Dunnett's test (C).

IHC analyses revealed that ABHD17C expression was significantly higher in KRAS^mut^‐PDAC tissues than that in KRAS^wt^‐PDAC tissues (**Figure** **5**A; Figure S5A, Supporting Information), and ABHD17C levels were inversely correlated with ALOX15B expression in KRAS^mut^‐PDAC (*p* < 0.001, R^2^ = 0.743; n = 95) but not in KRAS^wt^‐PDAC (*p* = 0.38, R^2^ = 0.08; n = 12) (Figure 5B). These results provided clinical evidence for ABHD17C upregulation on ALOX15B reduction in KRAS^mut^‐PDAC. Meanwhile, we found that ABHD17C upregulation was positively correlated with the TNM stage and relapse in KRAS^mut^‐PDAC (Tables S3 and S4, Supporting Information) and that KRAS^mut^‐PDAC patients with higher ABHD17C expression had shorter OS and RFS (*p* < 0.001, *p* < 0.001; Figure 5C). Therefore, these results indicate that ABHD17C upregulation is correlated with the clinical progression of KRAS^mut^‐PDAC.

### 2.7 ERK1 Phosphorylates and Stabilizes ABHD17C

We further investigated whether KRAS mutation contributed to ABHD17C upregulation. As shown in Figure 5D,E, overexpressing KRAS^G12D^ or KRAS^G12V^ mutant in KRAS^wt^‐PDAC cells led to obvious ABHD17C upregulation and ALOX15B downregulation, which was abolished by treatment with the MEK inhibitor U0126 or the ERK inhibitor ravoxertinib. Similarly, silencing KRAS or treatment with U0126 or ravoxertinib reduced ABHD17C expression and increased ALOX15B expression in KRAS^mut^‐PDAC cells (Figure 5F). However, no significant alterations occurred in the mRNA levels of *ABHD17C* and *ALOX15B* in these cells (Figure S5B, Supporting Information). We also found that inhibiting the KRAS/MAPK pathway accelerated ABHD17C degradation rate (Figure S5C, Supporting Information). However, treatment with the proteasome inhibitor MG132, but not autophagy inhibitor spautin‐1 or lysosome inhibitor NH_4_Cl, abrogated CHX‐mediated ABHD17C downregulation in KRAS^mut^‐PDAC cells (Figure S5D, Supporting Information). Taken together, these results suggest that the KRAS/MAPK pathway induces ABHD17C upregulation through posttranscriptional regulation.

Reciprocal co‐IP assay and far‐western blotting revealed that ABHD17C was directly bound to ERK1 but not ERK2, MEK, RAF1, or AKT1 (Figure 5G,H). AlphaFold3 analysis and co‐IP assay of serially truncated ABHD17C fragments further demonstrated that the kinase domain in the central region of ERK1 interacted with the hydrolase domain in the central region of ABHD17C (Figure 5I,J). Analysis of ABHD17C protein revealed 3 putative phosphorylation target sites, including S201, S233 and S266, for ERK1.^[^ ^31^ ^]^ Notably, only mutating S266 but not S201 and S233 to A in ABHD17C substantially reduced phosphorylated ABHD17C level in KRAS^mut^‐PDAC cells (Figure 5K). We then examined whether ERK1‐mediated phosphorylation modification was involved in ABHD17C stability. As shown in Figure 5L,M, ABHD17C‐S266A mutant, which could not be phosphorylated by ERK1, exhibited a shorter half‐life compared to ABHD17C‐wt protein and ABHD17C‐S201A or ‐S233A mutants, which was recovered by the proteasome inhibitor MG132 treatment. Importantly, mutation of S266 to E, which mimicked phosphorylation of ABHD17C, resulted in a drastically longer half‐life of ABHD17C (Figure 5L). These results indicated that the ABHD17C S266 residue was the ERK1 phosphorylation site, and ERK1‐mediated phosphorylation contributed to ABHD17C upregulation. Consistently, the K48‐linked polyubiquitination levels dramatically increased in the ABHD17C‐S266A mutant but decreased in the ABHD17C‐S266E mutant compared to the wild‐type ABHD17C (Figure 5N). Taken together, these results demonstrate that KRAS‐activating mutations contribute to phosphorylation and stability of ABHD17C protein.

Interestingly, treatment by MEK/ERK inhibitors dramatically decreased, but overexpression of KRAS^G12D^ mutant increased, the ABHD17C/ALOX15B interaction in KRAS^mut^‐PDAC and KRAS^wt^‐PDAC (Figure 5O). Moreover, co‐IP assays demonstrated that ALOX15B was associated more with the phosphomimic ABHD17C‐S266E mutant but less with ABHD17C‐S266A mutant (Figure 5P). Thus, these results suggest that KRAS/MAPK‐mediated phosphorylation increases the ABHD17C‐binding affinity of ALOX15B.

### 2.8 ABHD17C Inhibits Ferroptosis by Downregulating ALOX15B in KRAS^mut^‐PDAC

**Figure 6:**
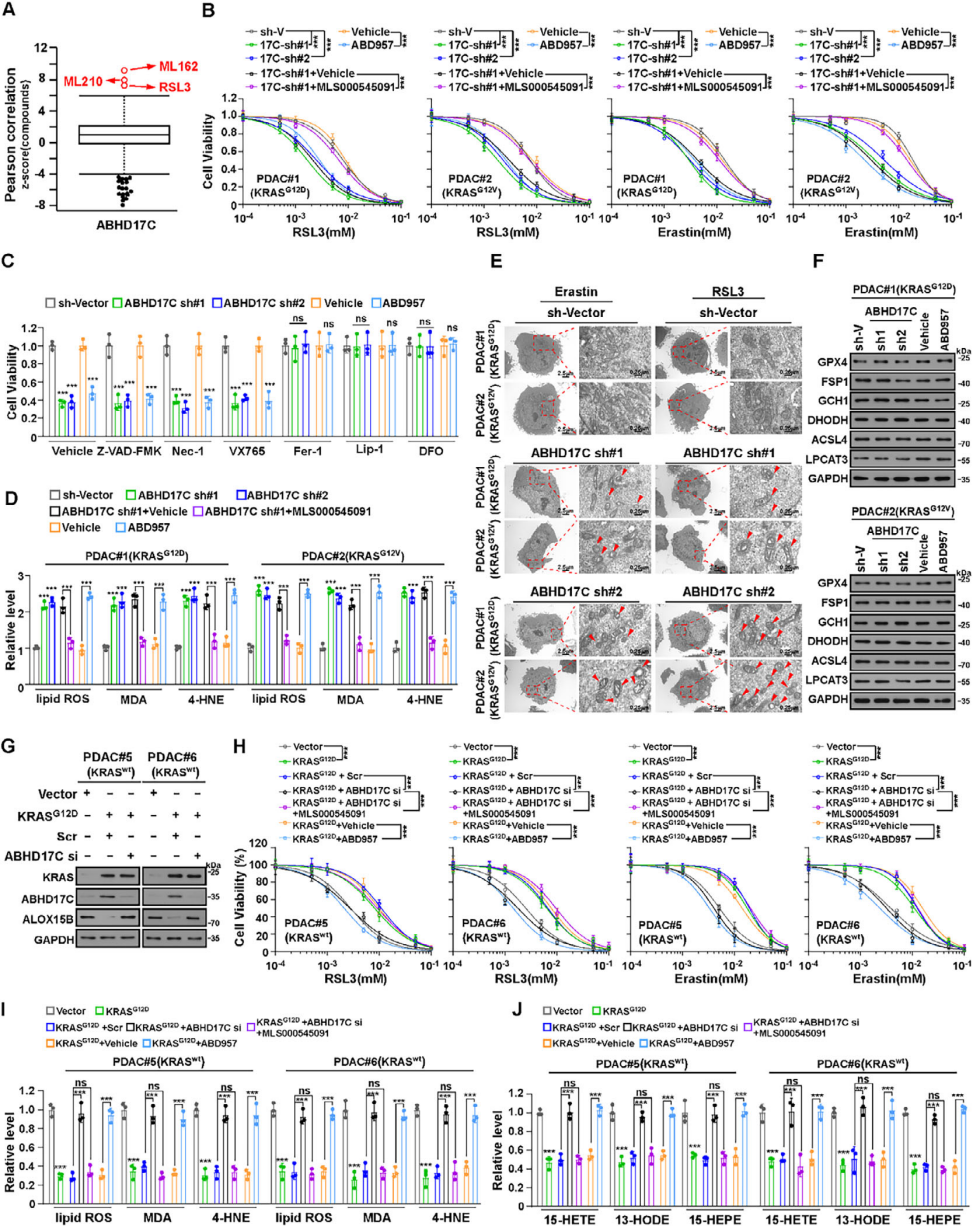
ABHD17C inhibits ferroptosis by downregulating ALOX15B in KRAS^mut^‐PDAC. A) High ABHD17C expression correlated with cancer cells’ resistance to ferroptosis inducers (RSL3, ML162, or ML210). The plotted data were mined from the CTRP database. The plotted values are Pearson's correlation coefficients. B) Cell viability analysis of indicated PDAC#1 (KRAS^G12D^) and PDAC#2 (KRAS^G12V^) cells treated with different concentrations of RSL3 and erastin for 8 and 16 h, respectively. C) Cell viability analysis of indicated cells treated with RSL3 (2 µm) plus vehicle, Z‐VAD‐FMK (10 µm), Nec‐1 (10 µm), VX765 (10 µm), Fer‐1 (2 µm), Lip‐1 (0.2 µm), or DFO (100 µm). D) Relative lipid ROS, MDA, and 4‐HNE levels in RSL3 (2 µM)‐treated indicated PDAC#1 (KRAS^G12D^) and PDAC#2 (KRAS^G12V^) cells. E) Transmission electron microscopy images showing mitochondrial crests in indicated PDAC#1 (KRAS^G12D^) and PDAC#2 (KRAS^G12V^) cells treated with erastin (5 µM) and RSL3 (10 µM) for 10 h. Scale bars: 2.5 µm (left) and 0.25 µm (right). F) IB analysis of GPX4, FSP1, GCH1, DHODH, ACSL4, and LPCAT3 expression in indicated cells. GAPDH was used as the loading control. G) IB analysis of KRAS, ABHD17C, and ALOX15B expression in the indicated cells. GAPDH was used as the loading control. H) Cell viability analysis of indicated PDAC#5 (KRAS^wt^) and PDAC#6 (KRAS^wt^) cells treated with different concentrations of RSL3 and Erastin for 8h and 16h, respectively. I) Relative lipid ROS, MDA, and 4‐HNE levels in indicated PDAC#5 (KRAS^wt^) and PDAC#6 (KRAS^wt^) cells treated with RSL3 (5 µm) for 10 h. J) Arachidonic acid metabolism assay analysis of relative levels of 15‐HETE, 13‐HODE, and 15‐HEPE in indicated PDAC#5 (KRAS^wt^) and PDAC#6 (KRAS^wt^) cells treated with RSL3 (5 µm) for 10 h. Each error bar in B, C, D, I, J represents the mean ± SD of 3 independent experiments (***P < 0.001, ns, not significant). Statistical analysis was performed using a two‐way ANOVA, followed by Tukey's test (B, C, D, I, J).

Consistent with the previously reported finding that ALOX15B converts AA to the proferroptotic 15‐HpETE and thus induces ferroptosis,^[^ ^18^, ^19^, ^20^ ^]^ overexpressing ALOX15B‐wt rather than ALOX15B‐C106A mutant in KRAS^mut^‐PDAC cells, increased RLS3‐ or erastin‐induced ferroptosis, the levels of 15‐HpETE metabolites (i.e. 15‐HETE, 15‐HODE, and 15‐HEPE), and the levels of lipid ROS, 4‐HNE, and MDA (Figure S6A–D, Supporting Information). We then examined whether KRAS^mut^/ABHD17C‐mediated ALOX15B reduction inhibited ferroptosis in KRAS^mut^‐PDAC. Analysis of the Cancer Therapeutics Response Portal (CTRP) revealed that ABHD17C expression was significantly correlated with cellular resistance against ferroptosis inducers, including RSL3, ML210, and ML162 (**Figure** **6**A), suggesting that ABHD17C might function as a ferroptosis suppressor. In line with this hypothesis, either silencing ABHD17C or treatment with the ABHD17C inhibitor ABD957 increased the RSL3‐ or erastin‐induced KRAS^mut^‐PDAC cell death (Figure 6B). However, only the ferroptosis inhibitors, including ferrostatin‐1 (Fer‐1), liproxstatin‐1 (Lip‐1), and deferoxamine (DFO) but not the apoptosis inhibitor Z‐VAD‐FMK, the necroptosis inhibitor necrostatin‐1(Nec‐1), or the pyroptosis inhibitor VX765, counteracted the ferroptosis induction in ABHD17C‐silenced cells (Figure 6C), suggesting that targeting ABHD17C had a specific inductive effect on ferroptosis sensitization. Furthermore, upon RSL3 treatment, ABHD17C‐silenced or ABD957‐treated KRAS^mut^‐PDAC cells exhibited significant increased level of AA metabolites, including 15‐HETE, 15‐HODE, and 15‐HEPE, elevated levels of lipid ROS and lipid peroxidation products 4‐HNE and MDA (Figure 6D; Figure S6E, Supporting Information). Meanwhile, we found that the RSL3‐ or erastin‐treated ABHD17C‐silenced cells displayed typical morphological characteristics of ferroptosis, specifically the disappearance of mitochondrial crestae (Figure 6E). Silencing ABHD17C or inhibiting ABHD17C activity did not affect GPX4, FSP1, GCH1, DHODH, ASCL4, and LPCAT3 expression (Figure 6F). Treatment with the ALOX15B inhibitor MLS000545091 abrogated the effects of ABHD17C inhibition‐induced ferroptosis, as indicated by cell viability assay, and AA metabolites, as shown by arachidonic acid metabolism assay, in KRAS^mut^‐PDAC cells (Figure 6B–D; Figure S6E, Supporting Information). Consistently, inhibition of ABHD17C resulted in significant ferroptosis and more AA metabolism in KRAS^G12D^‐transduced KRAS^wt^‐PDAC cells, which was significantly abolished by treatment with ALOX15B inhibitor MLS000545091 (Figure 6G–J). These results provided further evidence that KRAS^mut^/ABHD17C‐mediated ALOX15B downregulation may be vital in ferroptosis inhibition in KRAS^mut^‐PDACs.

### 2.9 Targeting ABHD17C Suppresses KRAS^mut^‐PDAC Tumor Growth by Inducing Ferroptosis

**Figure 7:**
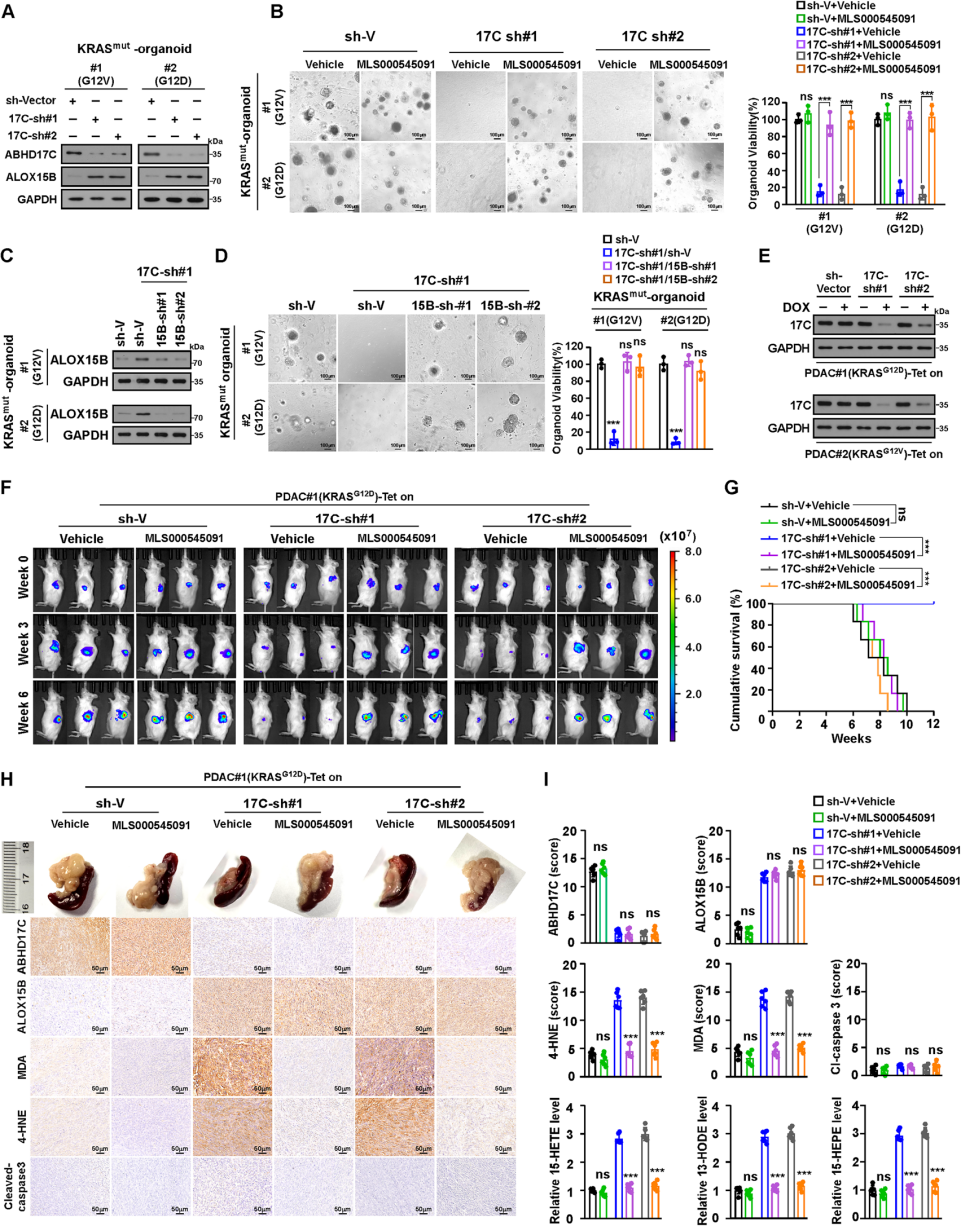
Targeting ABHD17C suppresses KRAS^mut^‐PDAC tumour growth via ferroptosis induction. A) IB analysis of ABHD17C and ALOX15B expression in indicated KRAS^mut^‐PDAC organoids. GAPDH was used as the loading control. B) Representative images (left) and quantification (right) of indicated KRAS^mut^‐PDAC organoids treated with vehicle or the ALOX15B inhibitor MLS000545091(1 µM). Scale bar: 100 µm. C) IB analysis of ALOX15B expression in indicated KRAS^mut^‐PDAC organoids. GAPDH was used as the loading control. D) Representative images (left) and viability quantification (right) of indicated KRAS^mut^‐PDAC organoids. Scale bar: 100 µm. E) IB analysis of ABHD17C expression in indicated DOX‐inducible Tet‐on PDAC#1 (KRAS^G12D^) and PDAC#2 (KRAS^G12V^) cells. GAPDH was used as the loading control. F) Representative images of indicated tumour‐bearing NOG mice treated with DOX and MLS000545091(50 mg kg^−1^) at indicated timepoints. G) Kaplan–Meier survival curves of mice orthotopically inoculated with indicated tumours. n = 6 mice/group. H) Representative IHC staining images from PDAC#1 (KRAS^G12D^) xenograft tumours with indicated treatments. I) IHC staining scores of ABHD17C, ALOX15B, 4‐HNE, MDA, and cleaved caspase‐3, and arachidonic acid metabolism assay analysis of relative levels of 15‐HETE, 13‐HODE, and 15‐HEPE (right). n = 6 mice per group. Data are the mean ± SD of n=3 (B, D) or n=6 (G, I) biologically independent samples (^***^ *p* < 0.001, ns, not significant). Statistical analysis was performed using a two‐way ANOVA, followed by Tukey's test (B, D, I), or a one‐way ANOVA, followed by Dunnett's test (G).

We then examined whether ABHD17C could serve as a potential therapeutic target for KRAS^mut^‐PDAC in vitro and in vivo. As shown in Figure S7A,B (Supporting Information) and **Figure** **7**A–D, silencing or inhibiting ABHD17C with ABD957 significantly reduced the growth rate of KRAS^mut^‐PDAC organoids, which was substantially rescued by ALOX15B inhibition or ALOX15B knockdown. These results provided further evidence that downregulation of ALOX15B was required for ABHD17C‐mediated KRAS^mut^‐PDAC progression.

We further established an in vivo mouse model by orthotopically xenografting DOX‐inducible ABHD17C‐silenced KRAS^mut^‐PDAC cells into the pancreas of NOG mice (Figure 7E,F). DOX was intraperitoneally injected when the BLI signal intensity reached 3.0 × 10^6^ p/s/cm^2^/sr. As shown in Figure 7F,G and Figure S7C,D (Supporting Information) DOX administration considerably suppressed ABHD17C expression and induced ALOX15B expression, but it significantly inhibited orthotopic growth of tumors formed by ABHD17C‐silenced KRAS^mut^‐PDAC cells, consequently resulting in longer mice survival. We also found that compared with control tumors, the levels of 3 ALOX15B metabolites, including 15‐HETE, 13‐HODE, and 15‐HEPE, and two LPO products, 4‐HNE and MDA, significantly increased in tumors formed by ABHD17C‐silenced‐KRAS^mut^‐PDAC cells (Figure 7H,I; Figure S7E,F, Supporting Information). However, no alterations were found in cleaved caspase‐3 levels in these tumors (Figure 7H,I; Figure S7E, Supporting Information). Taken together, these results indicated that targeting ABHD17C suppresses KRAS^mut^‐PDAC progression through ferroptosis induced by ALOX15B upregulation.

### 2.10 Methyl Protodioscin (MPD) Disrupts ABHD17C/ALOX15B Interaction

**Figure 8:**
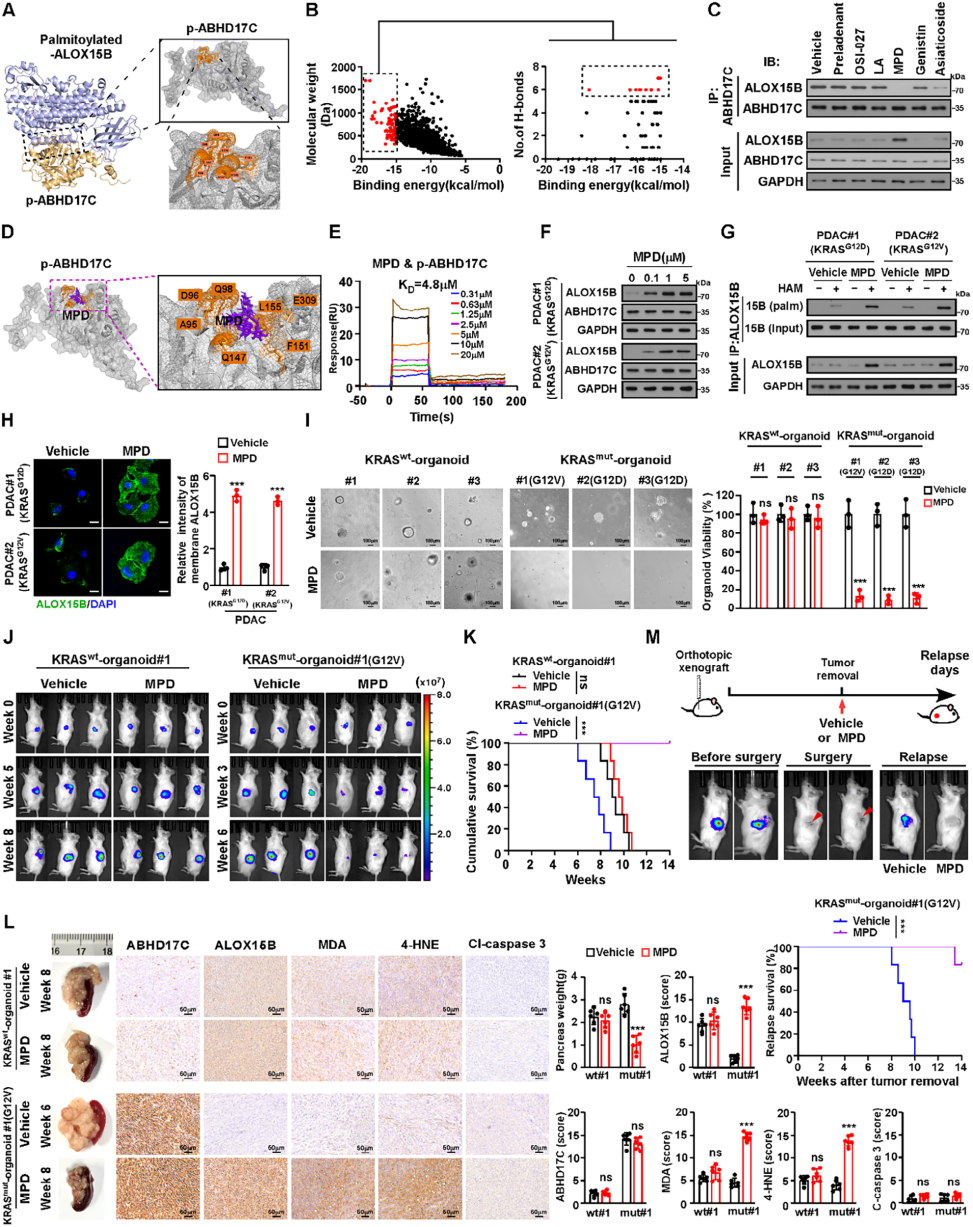
MPD inhibits KRAS^mut^‐PDAC progression. A) AlphaFold3 analysis of 3D structure of p‐ABHD17C–palmitoylated‐ALOX15B interaction region (left), and MOE‐Site Finder analysis of potential inhibitor binding pocket in p‐ABHD17C (right). B) Left: MOE‐Virtual screening of potential p‐ABHD17C‐binding small molecules in a bioactive compound library containing 22 478 small molecules. Selected molecules with binding energy of more than −15.00 kcal mol^−1^ are in red. Right: Affinity results of 77 small molecules with binding energy of more than 15.00 kcal mol^−1^. Selected molecules with more than 6 hydrogen bonds are in red. C) IP/IB analysis of inhibitory effect of 6 small molecules on ALOX15B/ABHD17C interaction. D) Left: Computational model analysis of MPD/p‐ABHD17C interaction. p‐ABHD17C is displayed as a grey wireframe, and MPD is shown as purple sticks. Architecture of p‐ABHD17C and MPD with interacting amino acids of p‐ABHD17C are denoted in orange. Right: 2D ligand interaction maps of cocrystals of p‐ABHD17C bound with MPD, generated using MOE. E) SPR assay with Biacore diagram and saturation curve of MPD binding to p‐ABHD17C. F) IB analysis of ALOX15B expression in MPD (1.0 µm)‐treated PDAC#1 (KRAS^G12D^) and PDAC#2 (KRAS^G12V^) cells. GAPDH was used as the loading control. G) ABE/IB analysis of palmitoylated‐ALOX15B expression in vehicle‐ or MPD (1.0 µm)‐treated cells. GAPDH was used as the loading control. H) Representative IF staining images (left) and quantification (right) of membrane ALOX15B in vehicle‐ or MPD (1.0 µMm)‐treated cells. Scale bar: 10 µm. I) Representative images (left) and quantification (right) of vehicle‐ or MPD (1.0 µm)‐treated KRAS^wt^‐PDAC and KRAS^mut^‐PDAC organoids. Scale bar: 100 µm. J) Representative images of vehicle‐ and MPD (1mg kg^−1^) ‐treated tumour‐bearing NOG mice, orthotopically inoculated with indicated organoids at indicated timepoints. K) Kaplan–Meier survival curves of NOG mice orthotopically inoculated with the indicated tumours; n = 6 mice per group. L) Representative IHC staining images (left), pancreas weight and scores of ABHD17C, ALOX15B, 4‐HNE, MDA, and cleaved caspase‐3 (right) in indicated tumour tissues. n = 6 mice/group. M) Top: Schematic of experimentation on relapse model. Middle: Orthotopic tumours formed by KRAS^mut^‐PDAC organoids were removed when the tumour volume reached ≈150 mm^3^. Then, the mice were treated with the vehicle or MPD (0.5 mg kg^−1^). Bottom: Kaplan–Meier of relapse survival of indicated tumour‐bearing mice. n = 6 mice per group. Data are the mean ± SD of n=3 (H, I) or n=6 (K, L, M) biologically independent samples (^***^ *p* < 0.001, ns, not significant.). Statistical analysis was performed using a two‐way ANOVA, followed by Tukey's test (H, I, L), or a one‐way ANOVA, followed by Dunnett's test (K, M).

Since our abovementioned results demonstrated that ABHD17C‐mediated ALOX15B reduction played a vital role in KRAS^mut^‐PDAC progression, which prompted us to screen for the inhibitor that may disrupt the ABHD17C/ALOX15B interaction. Analysis using PyMOL and Molecular Operating Environment (MOE) software‐Site finder module revealed that 8 core amino acids in p‐ABHD17C, including R94, A95, D96, Q98, Q147, M148, S150, and F151 that were required for the p‐ABHD17C/palmitoylated‐ALOX15B interaction, could form a pocket for inhibitor screening (**Figure** **8**A). Through structure‐based virtual screening from a bioactive compound library containing 22478 small molecules, 77 small molecule compounds were identified to bind to the pocket of p‐ABHD17C, which 6 small molecules showed high binding scores to the p‐ABHD17C pocket with at least interacting 5 amino acids and forming more than 6 hydrogen‐bond (Figure 8B).

We then tested the inhibitory effect of these 6 small molecules on ABHD17C/ALOX15B interaction. As shown in Figure 8C–H and Figure S8A, Supporting Information, treatment with methyl protodioscin (MPD) led to the highest disrupting effects on ABHD17C–ALOX15B interactions with a binding K_D_ of 4.8 µm, consequently resulting in elevation of total and palmitoylated ALOX15B expression and ALOX15B membrane localization. MPD treatment also inhibited the association of ALOX15B with the DCAF10/DDB1/CUL4 complex and reduced K48‐polyubiquitinated ALOX15B levels (Figure S8B,C, Supporting Information). Therefore, these results indicated that MPD‐mediated disruption of ABHD17C–ALOX15B interaction leads to ALOX15B upregulation and membrane localization.

### 2.11 MPD Inhibits KRAS^mut^‐PDAC Progression by Inducing Ferroptosis

In line with the promoting effects of MPD on upregulation and membrane localization of ALOX15B in KRAS^mut^‐PDAC cells, MPD significantly inhibited KRAS^mut^‐PDAC organoid growth rate; this effect was significantly reversed by the ferroptosis inhibitors Fer‐1, Lip‐1, and DFO but not by the apoptosis inhibitor Z‐VAD‐FMK, the necroptosis inhibitor necrostatin‐1 (Nec‐1), or the pyroptosis inhibitor VX765 (Figure 8I; Figure S8D, Supporting Information), suggesting that MPD has an inductive effect on ferroptosis in KRAS^mut^‐PDACs. Similarly, the levels of the cell death marker lactate dehydrogenase (LDH) and 15‐HpETE metabolites (15‐HETE, 15‐HODE, and 15‐HEPE) were significantly elevated in MPD‐treated KRAS^mut^‐PDAC cells but not in MPD‐treated KRAS^wt^‐PDAC cells (Figure S8E,F, Supporting Information). These results indicated that MPD induced ferroptosis in KRAS^mut^‐PDAC cells.

We then validated the therapeutic potential of MPD in vivo by orthotopic xenografting the luciferase reporter‐transduced KRAS^wt^‐ or KRAS^mut^‐PDAC organoids into the pancreas of NOG mice. When the BLI signal intensity of the pancreatic tumors reached 5 × 10^5^ p/s/cm^2^/sr, the mice were treated with vehicle or MPD. As shown in Figure 8J,K, and Figure S8G–I,Supporting Information, MPD significantly inhibited the growth of tumors formed by KRAS^mut^‐PDAC organoids, as indicated by decreased BLI signal intensity and size of the tumors, which resulted in prolonged survival of tumor‐bearing mice. However, the anti‐cancer effect of MPD was not significantly observed in tumors formed by KRAS^wt^‐PDAC organoids. In addition, compared with vehicle‐treated tumors, MPD‐treated tumors displayed elevated ALOX15B, 4‐HNE, and MDA levels. However, no alterations were found in cleaved caspase‐3 levels in these tumors. (Figure 8L; Figure S8J, Supporting Information). Thus, these results demonstrate the in vivo anti‐tumor effect of MPD on KRAS^mut^‐PDAC through inducing ferroptosis.

Furthermore, a recurrent PDAC mouse model was established in which pancreatic tumors were induced through orthotopic xenografting of KRAS^mut^‐PDAC organoids. These tumors were surgically excised when their BLI signal intensity reached 5 × 10^5^ p/s/cm^2^/sr; then, mice were administered with MPD or the vehicle (Figure 8M). Tumorectomy was considered successful when no luciferase signal was observed after tumor removal. Eight weeks after tumor removal, all 6 of 6 (100%) vehicle‐treated mice exhibited tumor recurrence, whereas 1 of 6 (16.6%) MPD‐treated mice displayed tumor recurrence (Figure 8M), indicating that MPD treatment inhibited KRAS^mut^‐PDAC recurrence.

**Figure 9:**
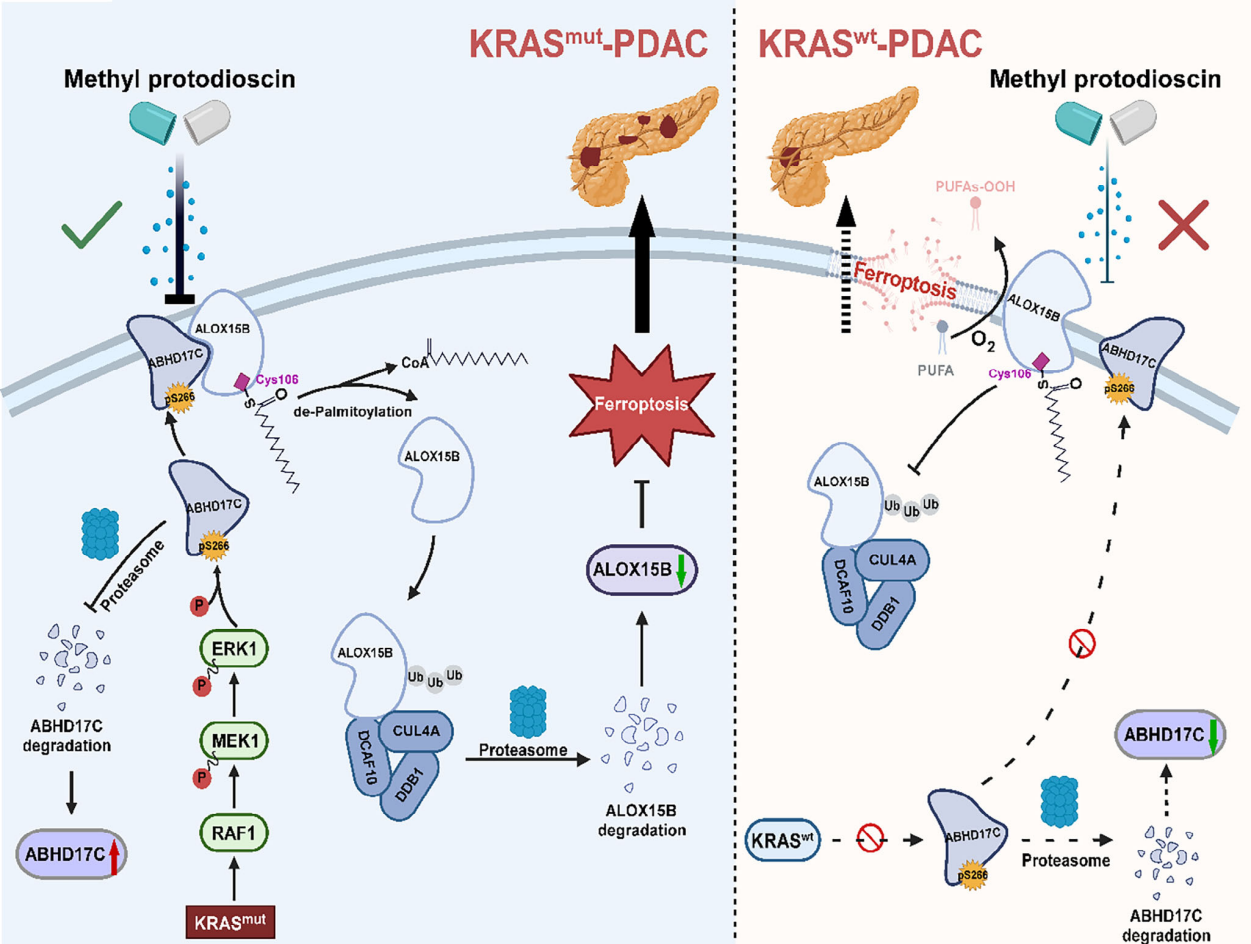
Hypothetical model. Phosphorylation of ABHD17C induced by KRAS^mut^/ERK signaling facilitates the depalmitoylation and subsequent translocation of ALOX15B from the membrane to the cytoplasm. This promotes proteasome‐dependent degradation of ALOX15B through its interaction with the E3 ligase complex CUL4/DDB1/DCAF10, thereby enabling KRAS^mut^‐PDAC cells to evade ferroptosis. Significantly, methyl protodioscin potently inhibites KRAS^mut^‐PDAC progression by disrupting the ABHD17C/ALOX15B interaction and inducing ferroptosis, thus representing a potential therapeutic strategy for KRAS^mut^‐PDAC.

## 3 Discussion

Pancreatic ductal adenocarcinoma (PDAC), with KRAS mutation frequency of more than 85%, is characterized by one of the highest mortality rates among all malignant diseases.^[^ ^32^, ^33^ ^]^ As a major oncogenic driver of PDAC, KRAS mutations are attractive targets for PDAC treatment. However, mutant KRAS has been considered undruggable due to the picomolar affinity of RAS for GTP, high intracellular levels of GTP, and the lack of deep binding pockets within RAS proteins to design small molecule inhibitors.^[^ ^34^, ^35^ ^]^ Although two KRAS^G12C^ inhibitors, Sotorasib and Adagrasib, have been FDA‐approved to treat NSCLC with KRAS^G12C^ mutations,^[^ ^36^, ^37^, ^38^, ^39^ ^]^ which is detectable only in 1–3% of all PDACs. The most common KRAS mutations in PDAC are G12D (45%), G12V (35%), and G12R (17%).^[^ ^3^, ^4^, ^32^ ^]^ Recent preclinical and clinical evidence also shows that the greatest barrier to KRAS^G12C^ inhibition therapy is the emergence of drug resistance due to various cellular, molecular, and genetic mechanisms.^[^ ^40^, ^41^, ^42^ ^]^ Thus, elucidating PDAC pathogenesis further in the context of activating KRAS signaling is essential for identifying promising therapeutic targets for PDAC. Herein, we demonstrated that phosphorylated ABHD17C mediated by KRAS^mut^‐activated ERK1 directly interacted with and depalmitoylated ALOX15B, leading to membrane‐to–cytoplasm translocation and proteasomal degradation of ALOX15B. Importantly, disrupting KRAS^mut^/ABHD17C/ALOX15B axis dramatically inhibited KRAS^mut^‐PDAC progression in vitro and in vivo via induction of ferroptosis. Therefore, our results shed light on the critical role of ferroptosis evasion induced by KRAS^mut^/ABHD17C/ALOX15B axis on PDAC progression and represent that restoration of ALOX15B might be a promising strategy against KRAS^mut^‐PDAC.

Moreover, we identified methyl protodioscin (MPD), a steroid saponin primarily purified from the rhizome of *Polygonatum sibiricum*, could restore membrane translocation and expression of ALOX15B via disruption of the ABHD17C/ALOX15B, consequently leading to inhibition of growth of KRAS^mut^‐PDAC organoids and KRAS^mut^‐PDAC‐formed tumors via ferroptosis induction. It has been previously reported that treatment with high concentration (15–20 µm) of MPD exhibited proapoptotic effects on pancreatic cancer cells.^[^ ^43^ ^]^ Herein, we found that treatment with a low concentration (1 µm) of MPD had no inductive effect on apoptosis in pancreatic cancer cells but significantly induced ferroptosis in KRAS^mut^‐PDAC cells, consequently resulting in significant improvement of the overall and relapse‐free survival of KRAS^mut^‐PDAC‐injected mice. Thus, MPD might be a promising medicine for the prevention and treatment of KRAS^mut^‐PDAC in future clinical trials.

ALOX15B, arachidonate 15‐lipoxygenase type II (15‐LOX‐2), is a crucial ferroptosis‐related lipid‐peroxidising enzyme, which catalyzes stereo‐specific peroxidation of PUFAs to generate a spectrum of bioactive lipid mediators.^[^ ^44^, ^45^, ^46^ ^]^ In line with the effect of ALOX15B on ferroptosis induction, overexpression of ALOX15B exhibits significant anti‐tumor activity in several in vitro and in vivo tumor models, which indicates that ALOX15B may act as a tumor suppressor. Consistently, ALOX15B has been found to be downregulated in various human cancer types through transcriptional or translational mechanisms.^[^ ^47^, ^48^ ^]^ For instance, glucocorticoid receptor (GR) transcriptionally represses *ALOX15B* mRNA expression in prostate cancer cells by directly binding to GR‐responsive element (GRE) on its promoter, and p53 loss results in *ALOX15B* mRNA suppression in bladder cancer.^[^ ^22^, ^48^ ^]^ Tumour‐associated macrophage‐derived exosomal miR‐660‐5p prevented ferroptosis in cervical cancer cells through *ALOX15* translation attenuation, and cancer‐associated fibroblast–secreted miR‐522 inhibited ferroptosis in gastric cancer via *ALOX15* translation suppression.^[^ ^49^, ^50^ ^]^ Intriguingly, we found that the KRAS^mut^‐PDAC cells exhibited higher *ALOX15B* mRNA levels but lower expression and shorter half‐life of ALOX15B than KRAS^wt^‐PDAC cells, suggesting that ALOX15B downregulation in KRAS^mut^‐PDAC might be regulated via unknown degradation mechanisms. We further found that KRAS^mut^/ABHD17C‐mediated depalmitoylation promoted the interaction of ALOX15B with the E3 ligase complex CUL4/DDB1/DCAF10, resulting in proteasome‐dependent ALOX15B degradation. Therefore, our results present here uncovered a novel mechanism underlying ALOX15B downregulation in cancer.

Another key finding of the current study is palmitoylation modification‐mediated membrane translocation of ALOX15B. Cytosolic ALOX15B exerts higher enzymatic activity for free AA oxidation but lower enzymatic activity for esterified AA‐phosphatidylethanolamine (AA‐PE) oxidation,^[^ ^51^, ^52^ ^]^ indicating that membrane translocation is essential for ALOX15B‐mediated oxygenated PLs. ALOX15B membrane translocation has been reported to involve multiple mechanisms.^[^ ^27^, ^53^, ^54^ ^]^ For instance, the amino‐terminal PLAT domain in ALOX15B contains a putative membrane insertion loop, which is flanked by two Ca^2+^‐binding sites, confers Ca^2+^‐dependent ALOX15B membrane localization.^[^ ^51^, ^55^ ^]^ Furthermore, Wenzel et al. demonstrated that PEBP1 contributes to ALOX15B membrane translocation by forming complexes with ALOX15B, predisposing ALOX15B to catalyze PUFA‐Pes hydroperoxidation.^[^ ^19^, ^27^ ^]^ However, PEBP1 expression is significantly downregulated in PDAC,^[^ ^56^, ^57^ ^]^ indicating that other mechanisms might be involved in ALOX15B membrane translocation in PDAC. In the current study, we found that treatment of PDAC cells with palmitoylation inhibitor 2‐BP or mutated ALOX15B C106 residue substantially reduced palmitoylation and membrane translocation of ALOX15B, whereas the ABHD17C inhibitor ABD957 increased them. Therefore, these results revealed a plausible mechanism underlying depalmitoylation modification–mediated membrane‐to‐cytoplasm translocation and degradation of ALOX15B. Currently, we are investigating the precise palmitoyltransferase responsible for ALOX15B palmitoylation, which remains unknown so far.

Oncogenic mutations in *KRAS* are detectable in nearly one‐quarter of a wide variety of cancers, predominantly in PDAC (86–90%), colorectal cancer (41%), and NSCLC (32%).^[^ ^34^, ^35^ ^]^ Due to a lack of pharmacologically targetable pockets within the mutant isoforms, KRAS has historically been considered ‘undruggable’. Therefore, improvements in the current understanding of KRAS signaling in cancer cells may aid in developing novel therapeutic strategies for KRAS‐mutant cancer. Our present results proved that KRAS^mut^/ABHD17C axis–mediated ALOX15B downregulation was functionally and clinically relevant to KRAS‐mutant cancer pathogenesis. Disruption of ABHD17C/ALOX15B interaction resulted in ALOX15B upregulation and ferroptosis in PDAC cells, consequently hindering KRAS^mut^‐PDAC progression (**Figure** **9**). Therefore, understanding the precise mechanisms underlying ALOX15B downregulation in KRAS‐mutant cancers may not only enhance the knowledge regarding the biological basis of KRAS mutation–mediated tumor initiation and progression but also enable the development of novel therapeutic strategies for patients with KRAS‐mutant cancers via pharmacological restoration of ALOX15B.

## 4 Experimental Section

### Cell Lines and Cell Culture

The following cell lines were obtained from the American Type Culture Collection (ATCC): HPNE (CRL‐4023), HPDE6‐C7 (HTX1979C), AsPC‐1 (CRL‐1682), BxPC‐3 (CRL‐1687), HPAC (CRL‐2119), MIA PaCa‐2 (CRM‐CRL‐1420), PANC‐1 (CRL‐1469MET), and CFPAC1 (CRL‐1918). The PANC‐1 and CFPAC1 cells were maintained in DMEM (Invitrogen) containing 10% FBS. The HPAC, AsPC‐1, BxPC‐3, MIA PaCa‐2, HPNE, HPDE6‐C7 cells were maintained in RPMI 1640 medium (Invitrogen) containing 10% FBS. All the cell lines were free of mycoplasma contamination and were authenticated by short tandem repeat (STR) fingerprinting at the Medicine Lab of Forensic Medicine Department of Sun Yat‐Sen University (China).

### Patient Information and Tissue Specimens

A total of 107 paraffin‐embedded, archived clinical PDAC specimens, including 12 KRAS^mut^‐PDAC and 95 KRAS^wt^‐PDAC, and 8 freshly collected PDAC and paired tumor‐adjacent normal tissues used in the current study were obtained from the patients diagnosed at the Sun Yat‐sen University Cancer Center, Sun Yat‐sen University from 2008 to 2012 according to the Institutional Research Ethics Committee Ethical Standards. The clinical information of those patients with PDAC was collected and exhibited in Tables S1–S4 (Supporting Information). The clinical tissues of fresh pancreatic cancer used in this study complied with all relevant ethical regulations for work with human participants. The study protocols were approved by the Institutional Research Ethics Committee of Sun Yat‐sen University for the use of these clinical materials for research purposes. All patients’ samples were obtained according to the Declaration of Helsinki and each patient signed written informed consent for all the procedures.

### Ethics Statement

This study complied with all of the relevant ethical regulations. All animal experiments were conducted under the approved protocol (L102012021040T) by the Institutional Animal Care and Use Committee of Sun Yat‐sen University. The use of human specimens and informed consent were approved by the ethics board of the Sun Yat‐sen University Cancer Center, Sun Yat‐sen University (Approved protocols B2022–639–01). Informed consent was obtained from the patients. No compensation was offered to any participant. Specimens were processed in the research laboratories.

### Statistical Analysis

The results were presented as the mean ± SD of three or six biologically independent experiments or samples. The results were analyzed using an unpaired Student's t‐test, one‐way ANOVA with Dunnett's multiple comparisons test, or two‐way ANOVA with Tukey's multiple comparisons test using GraphPad Prism. P values of 0.05 or less were considered statistically significant. No mice or data points were excluded from the analysis. All western blots, FACS data, and microscopy images were representative of at least three independent biological replicates. All biologically independent replicates have been explicitly stated in the respective figure legends. All samples and organisms were randomly allocated to the experimental groups. The animals were age‐ and sex‐matched. The investigators were blinded to the group allocation during collection and/or analysis. No statistical methods were used to pre‐determine sample sizes, but the sample sizes were similar to those reported in previous publications.^[^ ^9^, ^58^, ^59^ ^]^ The sample size and number of biological replicates were provided in the relevant figure legends.

## Conflict of Interest

The authors declare no conflict of interest.

## Author Contributions

Project planning was done by J.L. and M.L.; M.L. performed most experiments, analyzed data, and wrote the paper; X.Y. and Y.L. performed the animal experiments; S.O., L.W., and X.C. performed the cell biology experiments; H.Y., H.C, S.L., and Z.L. performed staining, immunohistochemical and pathological analysis, western blot and real‐time PCR; L.G., L.S., and J.L. conceived the idea, designed and discussed experiments, supervised progress and extensively edited and communicated regarding the manuscript.

## Supporting information



Supporting Information

## Data Availability

All datasets have been deposited and made publicly available: the quantitative proteomics massspectrometry data on KRAS^mut^‐PDAC and KRAS^wt^‐PDAC tissues (2 KRAS^G12D^‐PDAC, 1 KRAS^G12V^‐PDAC, and 3 KRAS^wt^‐PDAC tissues), KRAS^mut^‐PDAC and KRAS^wt^‐PDAC cells that support the findings of this study have been deposited to the ProteomeXchange Consortium (https://proteomecentral.proteomexchange.org) via the iProX partner repository with the dataset identifiers PXD058402 and PXD058462. RNA‐seq data on KRAS^mut^‐PDAC and KRASwt‐PDAC tissues (2 KRAS^G12D^‐PDAC, 1 KRAS^G12V^‐PDAC, and 3 KRAS^wt^‐PDAC tissues) that support the findings of this study have been deposited in the National Center for Biotechnology Information Sequence Read Archive with the accession code PRJNA1273307. All other data supporting the findings of this study are available from the corresponding authors upon reasonable request.
