# Supplementary material for: KRAS/ABHD17C/ALOX15B Axis Promotes Pancreatic Cancer Progression via Ferroptosis Evasion
Source: Adv Sci (Weinh). 2025 Jun 26;12(35):e04470. doi: 10.1002/advs.202504470 (PMC12463027; doi:10.1002/advs.202504470)

**Supporting Information**

**KRAS/ABHD17C/ALOX15B axis promotes pancreatic cancer progression via ferroptosis evasion**

*Man Li, Xuexin Yu, Yuanji Liu, Shuqin Ouyang, Long Wu, Xiaohong Chen, Huiqi Yu, Haoming Chen, Senmao Lian, Ziwen Li, Liyun Gong*, Libing Song*, Jun Li**

1. Supplementary Figures and Figure Legends……………………………………...Page 2-15

2. Supplementary Materials and Methods…………………………………….........Page 16-27

3. Supplementary Tables…………………………………………………………...Page 28-33

4. Source Data……………………………………………………………………...Page 34-47

**Supplementary Figures and Figure Legends**


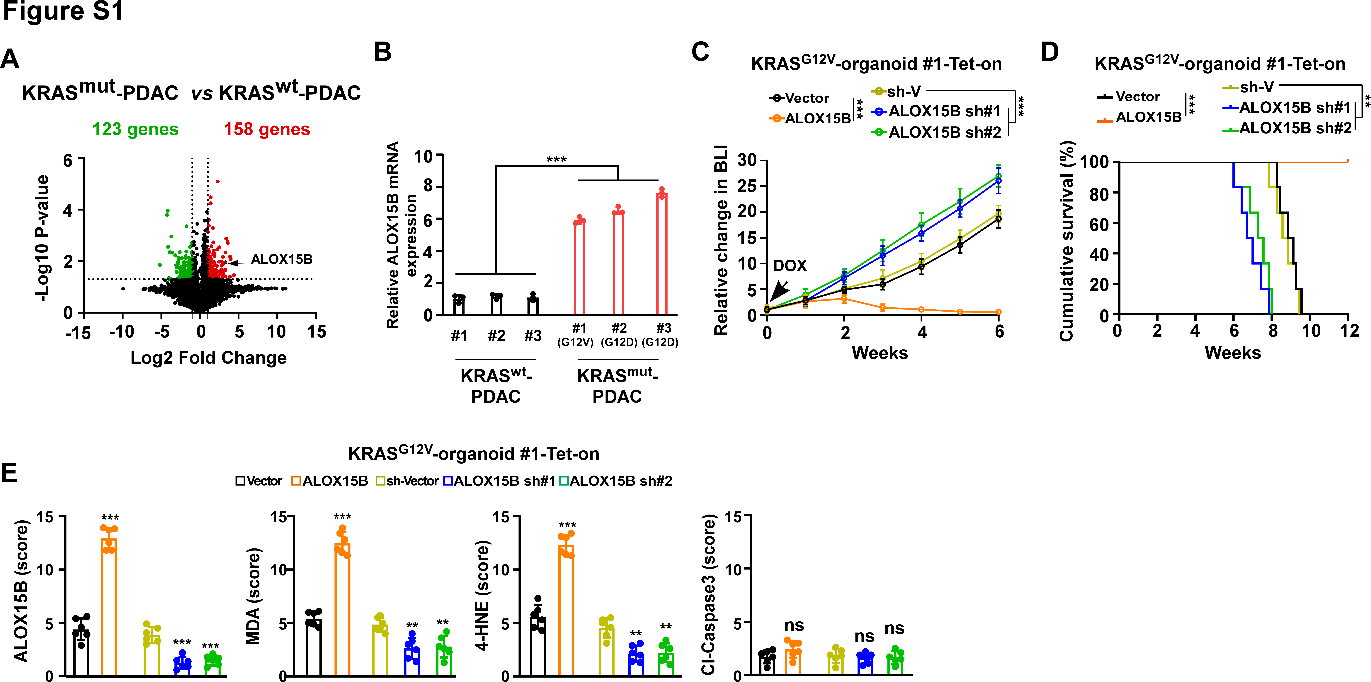


**Figure S1. ALOX15B downregulation promotes KRAS**^mut^**-PDAC tumour growth. A)** Volcano plot of dysregulated genes comparing KRAS^mut^-PDAC and KRAS^wt^-PDAC. **B)** Quantitative PCR of *ALOX15B* mRNA expression in indicated PDAC cells. *GAPDH* was used as the loading control. **C)** Relative changes in BLI signal intensities of pancreatic tumours formed by KRAS^G12V^-PDAC#1 organoid in NOG mice in response to DOX treatment. n = 6 mice/group. **D)** Kaplan–Meier survival curves of indicated mice. n = 6 mice/group. **E)** Quantification of ALOX15B, MDA, 4-HNE, and cleaved caspase-3 staining in KRAS^G12V^-PDAC#1 organoid xenograft tumours with indicated treatments. n = 6 mice/group. Each error bar represents the mean ± SD of 6 independent experiments (**P < 0.01, ***P < 0.001, ns, not significant.). Statistical analysis was performed using an unpaired two-tailed Student’s t-test (A), or a two-way ANOVA, followed by Tukey’s test (B, E), or one-way analysis of variance (ANOVA), followed by Dunnett’s test (C, D).


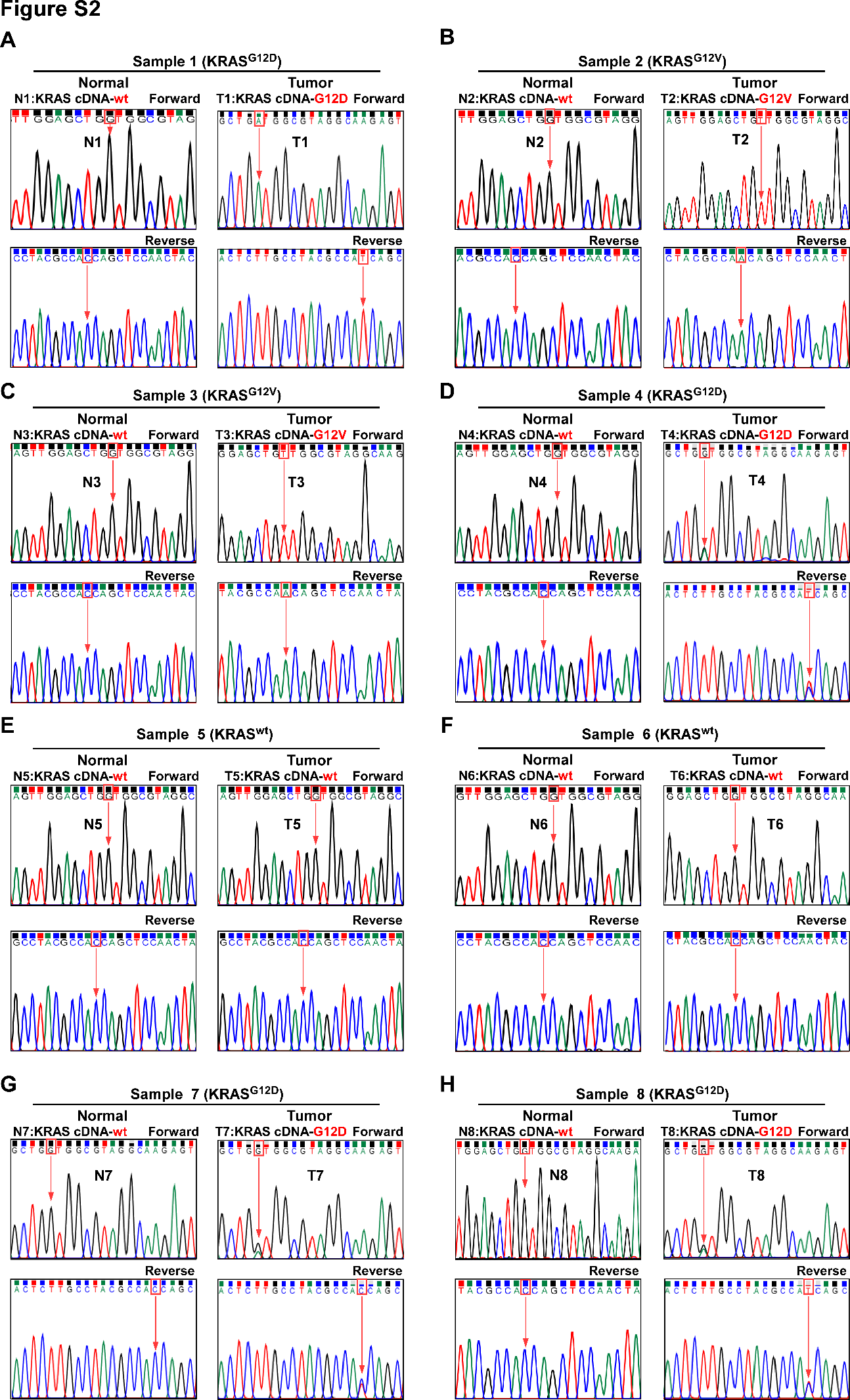


**Figure S2. DNA sequencing for KRAS^G12D^ and KRAS^G12V^ in 8 paired PDAC tissue samples. A–H)** DNA sequencing analysis showing that PDAC#1, PDAC#4, PDAC#7, and PDAC#8 tumours were KRAS^G12D^ mutants (A, D, G, and H), PDAC#2 and PDAC#3 tumours were KRAS^G12V^ mutants (B and C), and PDAC#5 and PDAC#6 tumours were KRAS^wt^ (E and F).

**
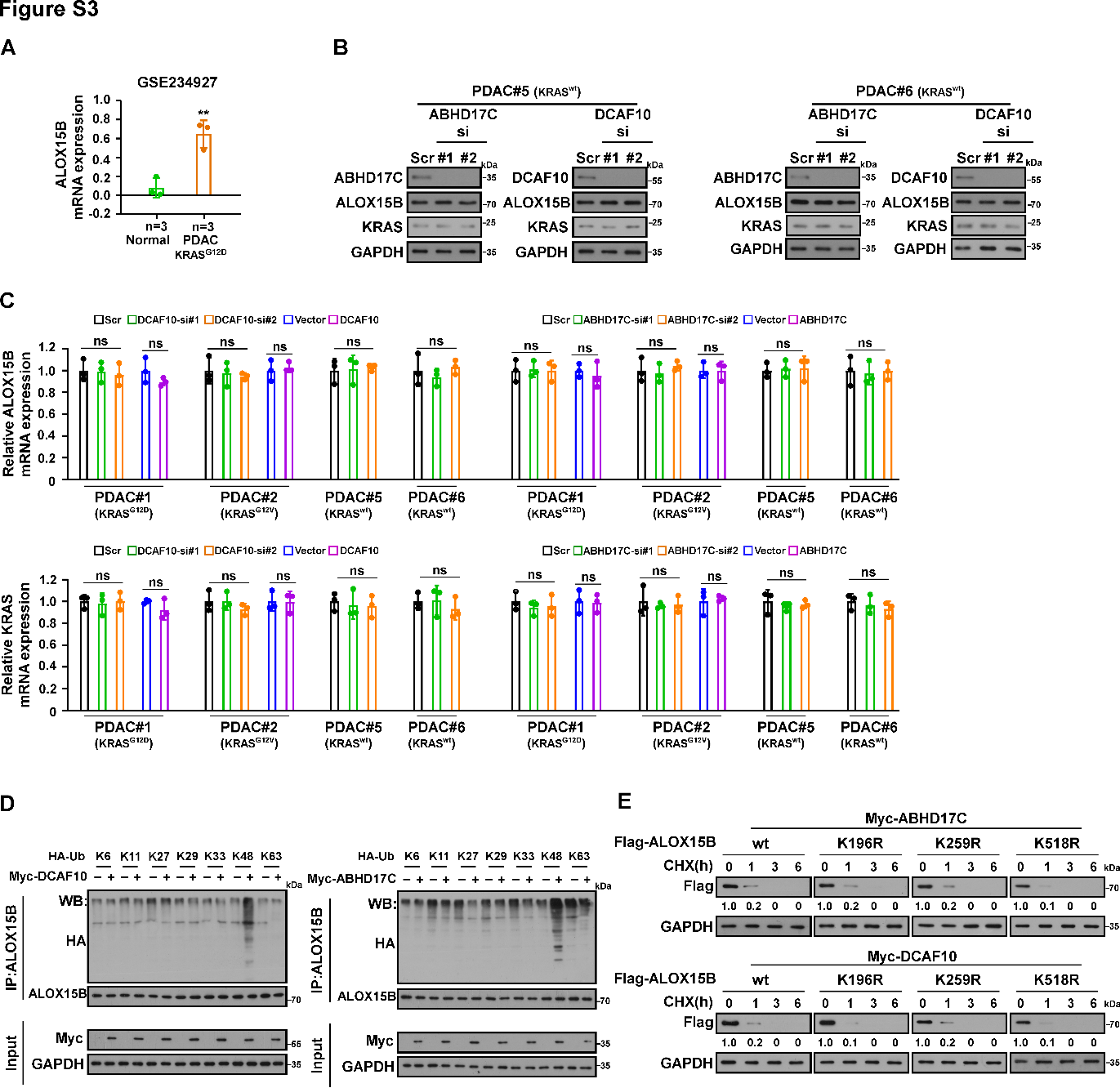
**

**Figure S3. E3 ligase complex CUL4/DDB1/DCAF10 promotes ALOX15B degradation. A)** GSE234927 dataset analysis of *ALOX15B* mRNA expression in three paired KRAS^G12D^-PDAC and normal pancreatic tissues. **B)** IB analysis of ALOX15B, DCAF10, ABHD17C, and KRAS expression in indicated cells. GAPDH was used as the loading control. scr: scramble siRNA. **C)** Quantitative PCR analysis of *ALOX15B* and *KRAS* mRNA expression in indicated cells. *GAPDH* was used as the loading control. **D)** IB analysis of expression of polyubiquitinated-ALOX15B in the cells transfected with different HA-tagged ubiquitin (HA-Ub) mutants with or without myc-DCAF10 and ABHD17C. **E)** CHX chase analysis of half-life of the indicated ALOX15B mutants in myc-ABHD17C-(upper) or myc-DCAF10-(lower) transduced cells at indicated timepoints. GAPDH was used as the loading control. Protein quantified against controls, set as 1.0. Each error bar represents the mean ± SD of three independent experiments (**P < 0.01, ns, not significant.). Statistical analysis was performed using an unpaired two-tailed Student’s t-test (A), or a two-way ANOVA, followed by Tukey’s test (C).


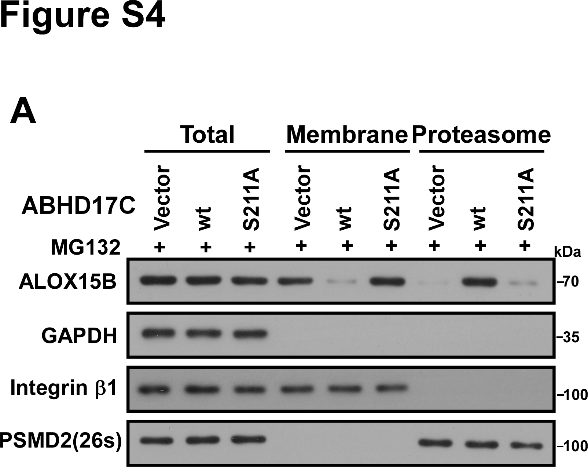


**Figure S4. ABHD17C overexpression promotes ALOX15B degradation. A)** IB analysis of expression of ABHD17C, ALOX15B in total, membrane fractions and proteasome fractions in vector-, ABHD17C/wt-, or ABHD17C/S211A-trasfected cells treated with MG132.


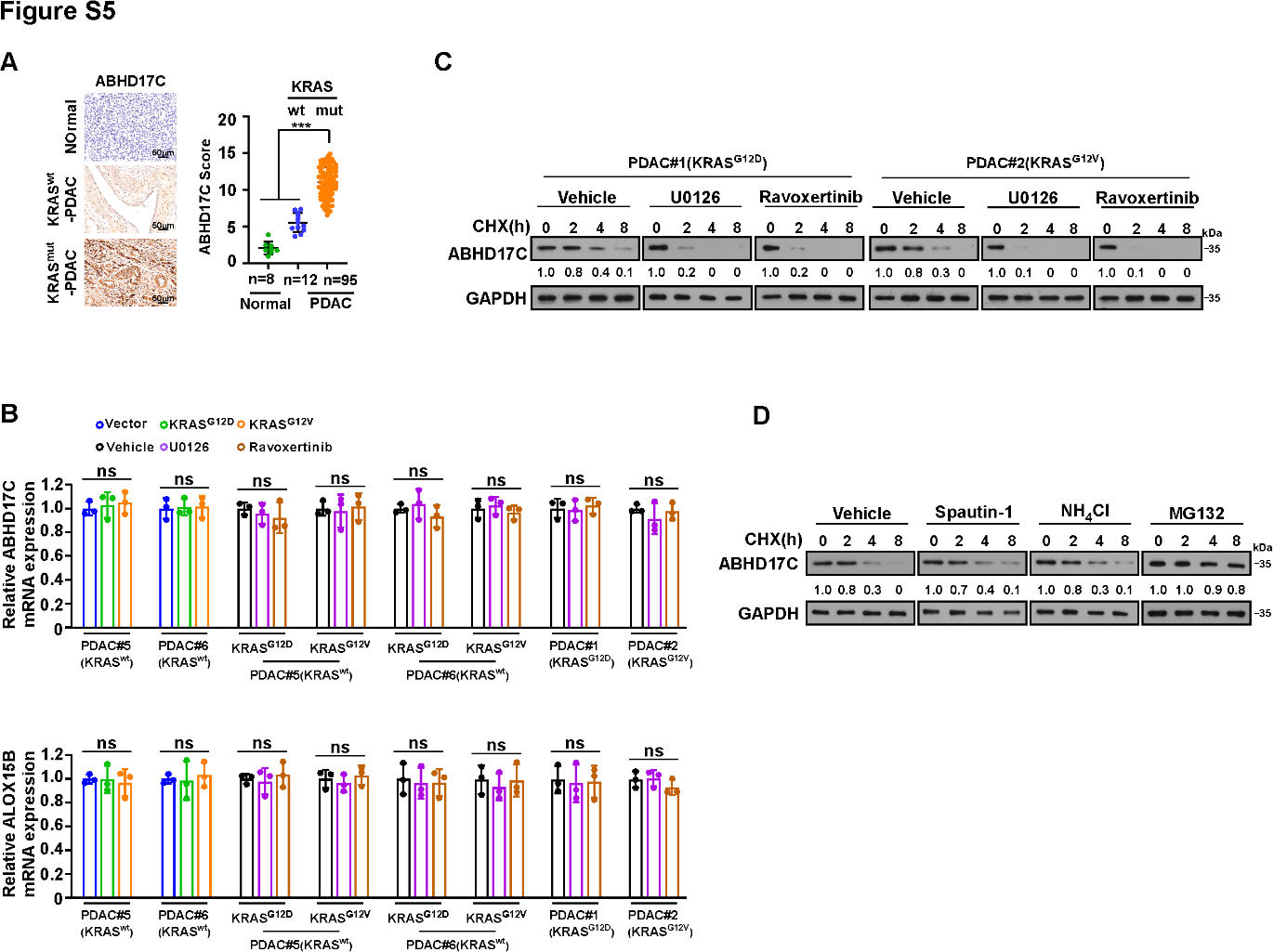


**Figure S5. ERK1 phosphorylates and stabilises ABHD17C. A)** Representative IHC staining images (left) and quantification (right) of ABHD17C expression in normal pancreatic tissues (n = 8), KRAS^wt^-PDAC tissues (n = 12), and KRAS^mut^-PDAC tissues (n = 95). Scale bar: 50 μm. **B)** Quantitative PCR of *ABHD17C* and *ALOX15B* mRNA expression in indicated cells. *GAPDH* was used as the loading control. **C)** CHX chase analysis of half-life of ABHD17C in vehicle-, U0126 (10 μM)-, or ravoxertinib (0.1 μM) -treated cells at indicated timepoints. GAPDH was used as the loading control. Protein quantified against controls, set as 1.0. **D)** CHX chase analysis of ABHD17C half-life in the vehicle-, spautin-1 (5 μM)-, NH_4_Cl (20 mM) -, or MG132 (10 μM)-treated cells at indicated timepoints. GAPDH was used as the loading control. Protein quantified against controls, set as 1.0. Each error bar represents the mean ± SD of three independent experiments (***P < 0.001 ns, not significant.). Statistical analysis was performed using an unpaired two-tailed Student’s t-test (A), or a two-way ANOVA, followed by Tukey’s test (B).


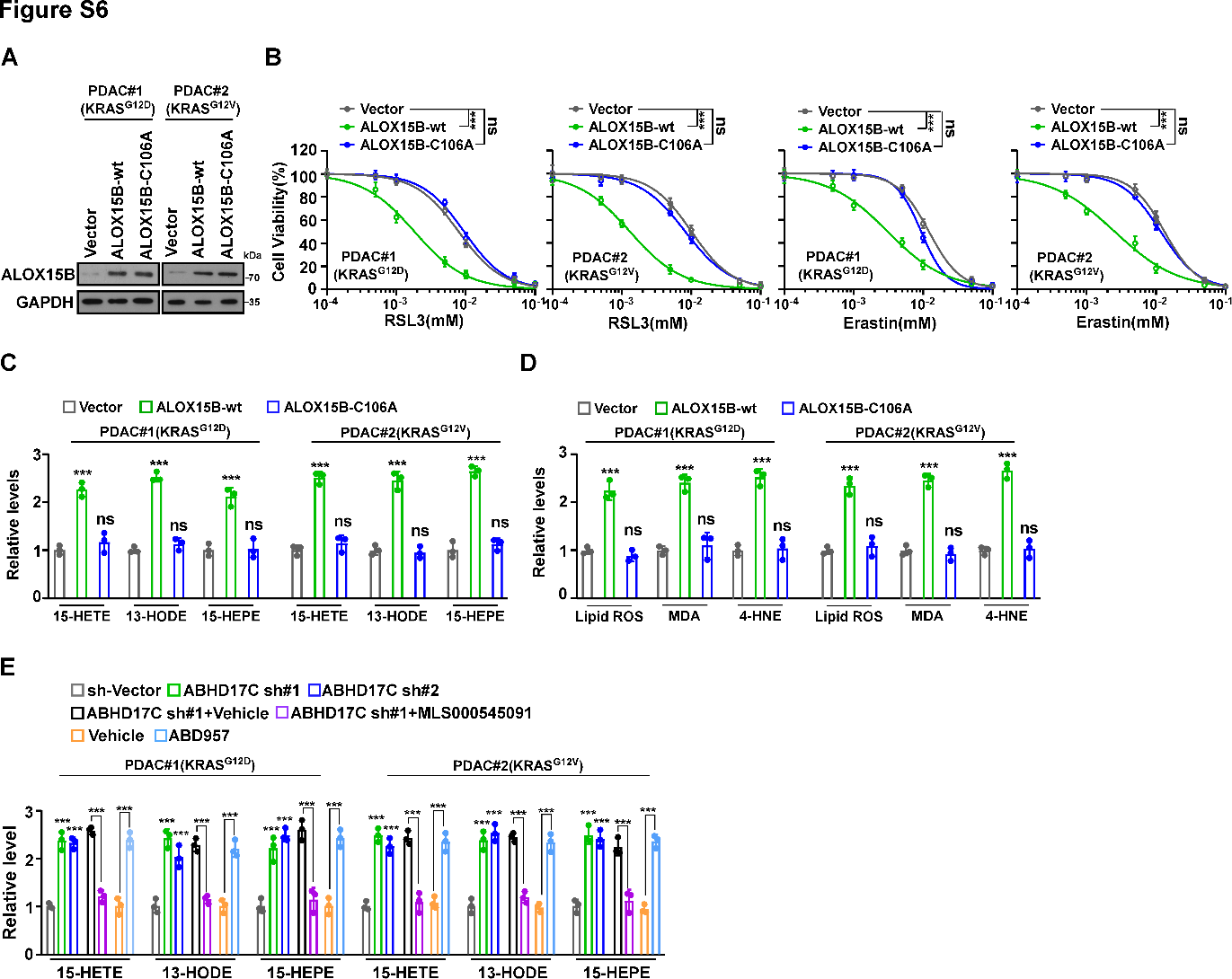


**Figure S6. ABHD17C inhibits ferroptosis by downregulating ALOX15B. A)** IB analysis of ALOX15B expression in the indicated cell transfected with vector (5 μg), ALOX15B (5 μg), or ALOX15B-C106A (15 μg). **B)** Cell viability analysis of vector-, ALOX15B-, or ALOX15B-C106A-transduced cells treated with different concentrations of RSL3 and Erastin for 8 h and 16 h, respectively. **C)** Arachidonic acid metabolism assay analysis of relative levels of 15-HETE, 13-HODE, and 15-HEPE in the vector-, ALOX15B-, or ALOX15B-C106A-transduced cells. **D)** Relative levels of lipid ROS, MDA, and 4-HNE in vector-, ALOX15B-, or ALOX15B-C106A-transduced cells. **E)** Arachidonic acid metabolism assay analysis of relative levels of 15-HETE, 13-HODE, and 15-HEPE in indicated PDAC#1 (KRAS^G12D^) and PDAC#2 (KRAS^G12V^) cells. Each error bar represents the mean ± SD of three independent experiments (***P < 0.001, ns, not significant.). Statistical analysis was performed using a two-way ANOVA, followed by Tukey’s test (B-E).


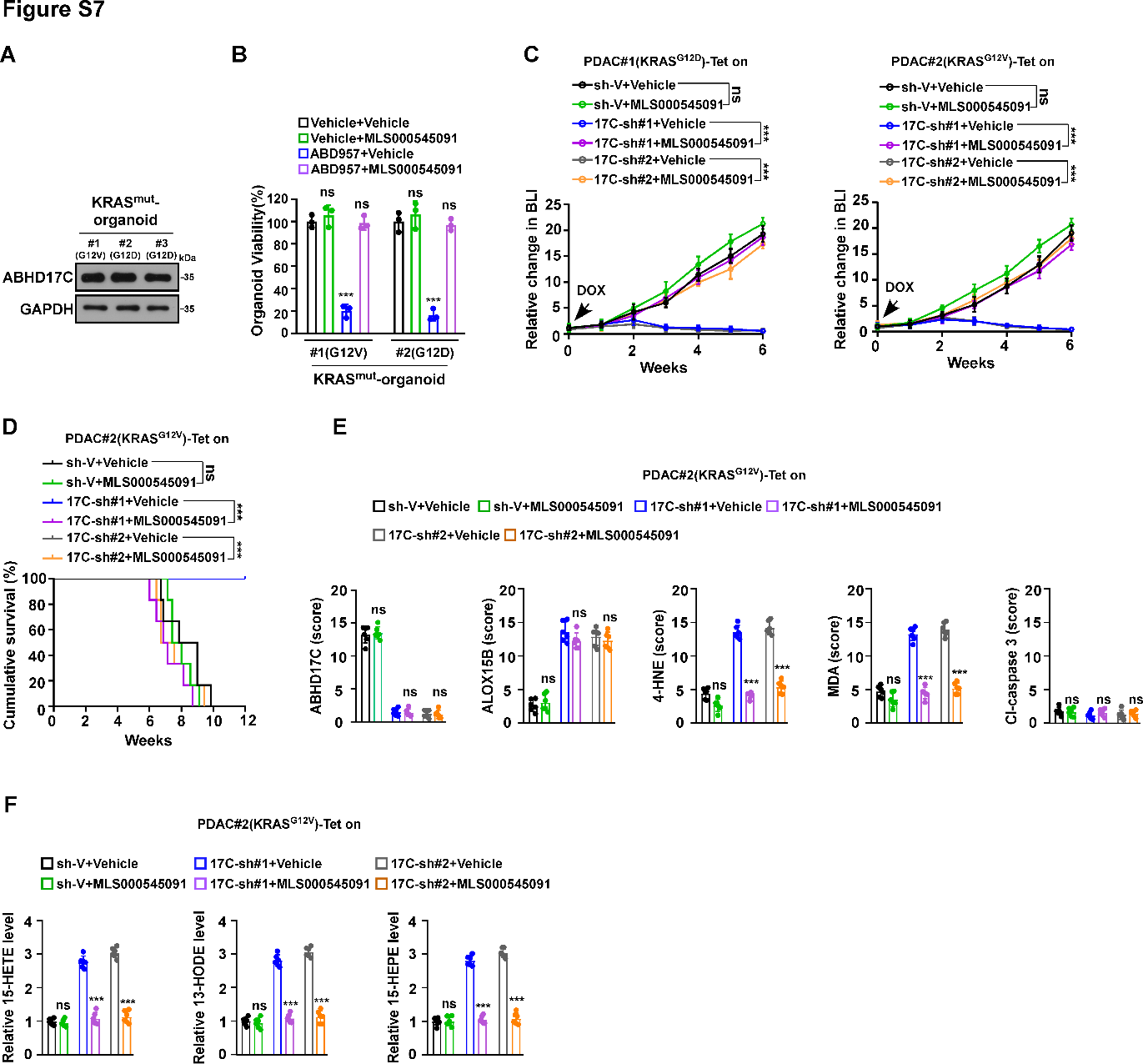


**Figure S7. Targeting ABHD17C suppresses KRAS^mut^-PDAC tumour growth via ferroptosis induction. A)** IB analysis of ABHD17C expression in indicated KRAS^mut^-PDAC organoids. GAPDH was used as the loading control. **B)** Quantification of indicated KRAS^mut^-PDAC organoids treated with vehicle or the ABHD17C inhibitor ABHD957(1 μM) plus vehicle or the ALOX15B inhibitor MLS000545091(1 μM). **C)** Relative change in BLI signal of indicated DOX-treated pancreatic tumours in NOG mice. n = 6 per group. **D)** Kaplan–Meier survival curves of NOG mice orthotopically inoculated with the indicated tumours. n = 6 mice/group. **E)** Staining scores of ABHD17C, ALOX15B, 4-HNE, MDA, and cleaved caspase-3 in tumour-bearing NOG mice orthotopically inoculated with indicated PDAC#2 (KRAS^G12V^) cells and treated with DOX and MLS000545091(50 mg/kg) at indicated timepoints. **F)** Arachidonic acid metabolism assay analysis of relative levels of 15-HETE, 13-HODE and 15-HEPE in tumour-bearing NOG mice orthotopically inoculated with the indicated PDAC#2 (KRAS^G12V^) cells and treated with DOX and MLS000545091(50 mg/kg) at indicated timepoints. n = 6 mice/group. Each error bar represents the mean ± SD of 6 independent experiments (***P < 0.001, ns, not significant.). Statistical analysis was performed using a two-way ANOVA, followed by Tukey’s test (B, E, F), or a one-way ANOVA, followed by Dunnett’s test (C, D).


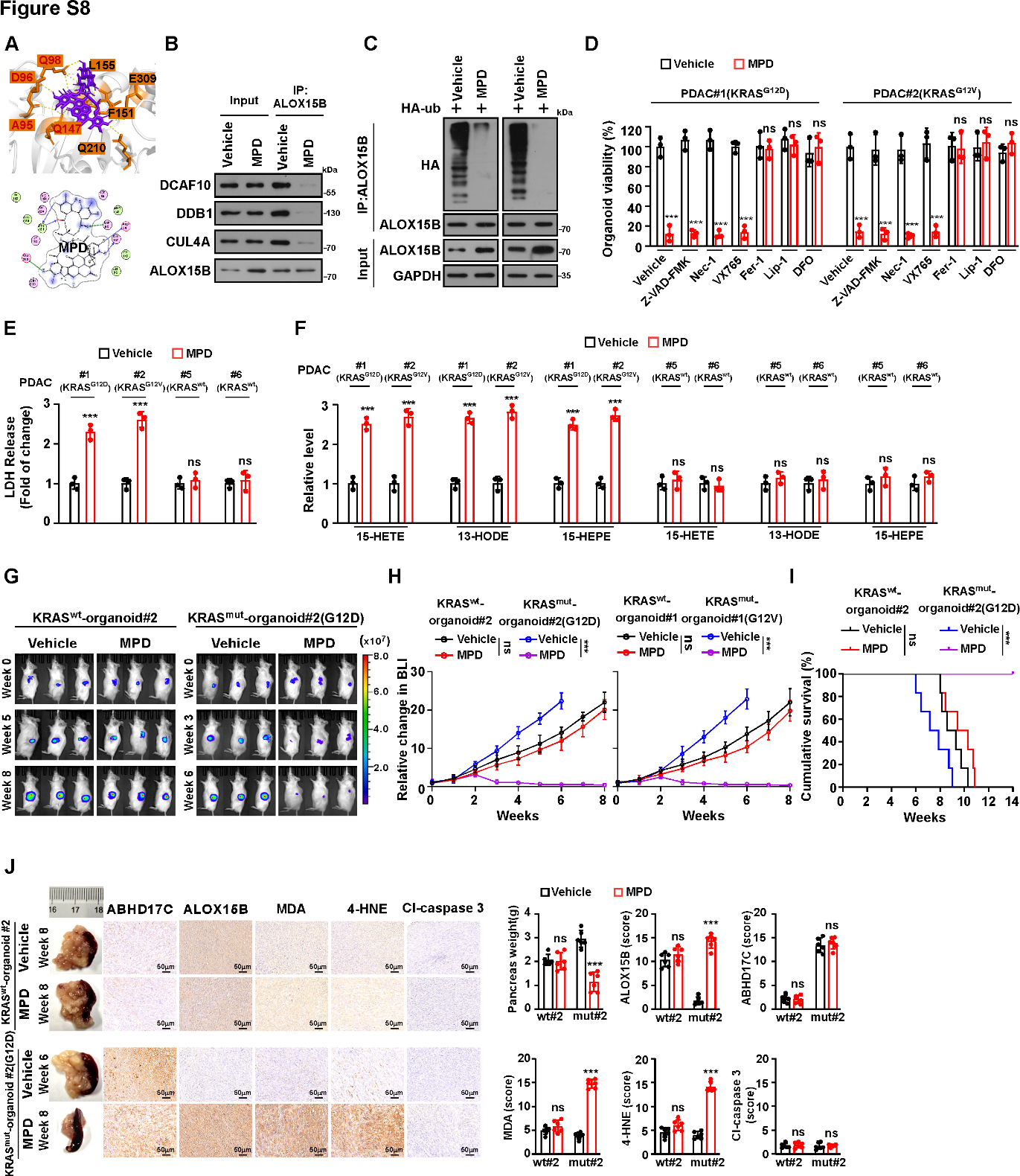


**Figure S8. MPD inhibits KRAS^mut^-PDAC progression. A)** Computational model analysis of MPD–p-ABHD17C interaction. **B)** IP/IB analysis of interactions of ALOX15B with DCAF10, DDB1, and CUL4A in vehicle- or MPD-treated cells. **C)** IP/IB analysis of polyubiquitinated-ALOX15B expression in vehicle- or MPD-treated cells. **D)** Organoid viability analysis of the indicated vehicle- or MPD-treated KRAS^mut^-PDAC organoids treated with vehicle, Z-VAD-FMK (10 μM), Nec-1 (10 μM), VX765 (10 μM), Fer-1 (2 μM), Lip-1 (0.2 μM), or DFO (100 μM). **E-F)** Arachidonic acid metabolism assay analysis of relative levels of LDH (**E**) and 15-HETE, 13-HODE, and 15-HEPE (**F**) in indicated vehicle- or MPD-treated KRAS^mut^-PDAC cells. **G)** Representative images of vehicle and MPD-treated tumour-bearing NOG mice, orthotopically inoculated with indicated organoids at indicated timepoints. **H)** Relative change in BLI signal intensity of indicated DOX-treated pancreatic tumours in NOG mice. n = 6 mice/group. **I)** Kaplan–Meier of survival of indicated tumour-bearing mice. n = 6 mice/group. **J)** Representative IHC staining images (left) and staining scores for ABHD17C, ALOX15B, 4-HNE, MDA, and cleaved caspase-3 (right) in the indicated tumour tissues. n = 6 mice/group. Each error bar represents the mean ± SD of n=3(D, E, F) or n=6 (H, I, J) independent experiments (***P < 0.001 ns, not significant.). Statistical analysis was performed using or a two-way ANOVA, followed by Tukey’s test (D, E ,F, J), or a one-way ANOVA, followed by Dunnett’s test (H,I).

**Supplementary Materials and Methods**

**Patient-Derived Organoids Culture and Treatments**

Fresh surgical PDAC tumor tissues were obtained from the Sun Yat-sen University Cancer Center. Before sampling, written informed consent was obtained from all patients. In brief, freshly excised tumor tissues were immediately digested using 2 mg/ml collagenase (Cat: C9407; Sigma Aldrich) at 37 °C for 12 h, and the dissociated cells were coated in growth factor-reduced Matrigel (Cat: 356231; Corning). This was followed by Matrigel–cell solution solidification in 48-well plates and then by immediate administration of PDAC organoid medium (Accurate International Biotech); this medium was refreshed every 2–3 days. Organoids were collected for IB analysis after 3-4 weeks of culture. Subsequently, organoids were subcultured approximately every 14-21 days. Organoid cell viability was measured using a CellTiter-Glo 3D cell viability assay (Cat: G9683; Promega), according to the manufacturer's instructions. To overexpress or knockdown genes in organoids, the dome was digested by collagenase to get a single-cell suspension. Then, the suspension was mixed with lentiviruses and polybrene. The mix was centrifuged at 300 RCF(g) for an hour at room temperature and then incubated at 37°C for 3 hours. The cells were then plated with Matrigel and grown in PDAC organoid medium for further culture. To construct the organoid xenograft model, the dome was digested by collagenase to get a single-cell suspension. The cells were then counted, and the protocol for constructing the orthotopic xenograft model was followed.

**Orthotopic cell line-derived xenograft (CDX) model**

All of the animal procedures were approved by the Sun Yat-sen University Animal Care Committee. NOD-SCID IL-2rγ^−/−^ (NOG) mice (4–6 weeks old) were obtained from Beijing Vital River (Beijing, China). In brief, mice were injected with 5 × 10^5^ indicated cells expressing luciferase in the pancreas of mice, respectively (n = 6 per group). Orthotopic xenograft bioluminescence was examined every 3 days. Doxycycline was dissolved and administered in mouse drinking water at concentrations of 0.5-8 mg/ml for 12 weeks. MLS000545091 (50 mg/kg) and Methyl protodioscin (MPD, 0.5 mg/kg) were intraperitoneally injected. The tumors were observed and assessed weekly in mice injected with D-luciferin (75 mg/kg) by bioluminescence imaging using the IVIS Spectrum In Vivo Imager. After 12 weeks, mice-bearing PDAC were executed. Experiments associated with animals comply with the Laboratory Animal Care and Use Guide and the Institutional Research Ethics Committee has approved these experiments.

**Relapse model**

Five-week-old male NOG mice were orthotopic implantation with 5×10^5^ indicated cells. When BLI signal of tumors reached 5 × 10^5^ p/s/cm^2^/sr, mice were randomly divided into four groups (n = 6 per group) and then received surgical resection to remove the pancreatic tumors. After surgery, mice were treated with vehicle (control), MPD (0.5 mg/kg, twice/week) for 14 weeks. The survival time from surgical resection to relapse was calculated and determined as relapse-free survival.

**Reagents**

The reagents used in cell experiments in the article include protein synthesis inhibitor cycloheximide (Cat: HY-12320, MCE); Proteasome inhibitor MG132 (Cat: M8699, Sigma-Aldrich); autophagy inhibitor Spautin-1 (Cat: HY-12990, MedChemExpress); lysosome inhibitor NH_4_Cl (Cat: A9434, Sigma-Aldrich); Palmitoylation inhibitor 2-bromopalmitate (2-BP, Cat: 21604, Sigma-Aldrich); Acyl protein thioesterase 1 palmostatin B (Cat: HY-120911, MCE), ML348(Cat: HY-100736, MCE); ABHD17C inhibitor ABD957(Cat: HY-142161, MCE); MEK/ERK inhibitors U0126 (Cat: HY-12031A, MCE) , Ravoxertinib (Cat: HY- HY-50706, MCE); Ferroptosis inducer RSL3 (Cat: S8155, Selleck), Erastin (Cat: S7242, Selleck); ferroptosis inhibitors Fer-1 (Selleck, S7243), Lip-1 (Selleck, S7699), DFO (Sigma-Aldrich, D9533); the apoptosis inhibitor Z-VAD-FMK (Selleck, S7023); the necroptosis inhibitor Nec-1 (Selleck, S8037); Pyroptosis inhibitor VX765 (Selleck, S2228); ALOX15B inhibitor MLS000545091 (Cat: HY-118110, MCE), Preladenant (Cat: HY-10889, MCE), OSI-027 (Cat: HY-10423, MCE), Lithospermic acid (Cat: HY-N0823, MCE), Methyl protodioscin(Cat: HY-N0863, MCE), Genistin (Cat: HY-N0595, MCE), Asiaticoside (Cat: HY-N0439, MCE)

**Plasmids, retroviral infection, and transfection**

The human ABHD17C (Full, S201A, S233A, S266A, S266E), DCAF10 (Full), KRAS^G12V^, KRAS^G12D^ , ALOX15B (Full, K175R, K196R, K259R, K318R, C89A, C106A,161A, C254A, C395A, C455A, C549A) and P4HA2, HBD, ARMCX3, RPL29, HGS, CUL1, F5, PZP, MGLL, VTN, LRP1, CCN1, CLIC4, STAT6, TPM4, GAPVD1, LTF, ALB, CALU, ACAD9, F2, P3H1, AHSG, PSAT1, DAB2, TPM2, GOLIM4, GNA11,TJP1, FNDC3B, TRIOBP, LACTB, GOPC, SH3BGRL, HSPG2, COPG2, SACM1L, IFITM2 and SERPINF2 were cloned into the pSin-EF2 vector and pLVX-TRE3G-IRES vector. shRNAs targeting ABHD17C, ALOX15B or DCAF10 were cloned into the pSuper retroviral vector and pLVX-TRE3G-IRES vector. Transfection of siRNAs or plasmids was performed using the Lipofectamine 3000 reagent (Thermo Fisher Scientific, Waltham) according to the manufacturer’s instruction. All primers and oligonucleotides are listed in Table S5, Supporting Information. Stable cell lines expressing ALOX15B, ABHD17C or DCAF10 or corresponding shRNAs were generated via retroviral infection using 293T cells and selected for 10 days with 0.5 µg/mL puromycin 48 h after infection.

**RNA extraction, reverse transcription, and real-time PCR**

The total RNA was extracted from an indicated cells or tissues using the Trizol (Life Technologies) reagent according to the manufacturer’s instructions. Real-time reverse transcription-polymerase chain reaction (PCR) primers and probes were designed with the assistance of the Primer Express v 2.0 software (Applied BioSystems, Foster, CA, USA). Expression data were normalized to the geometric mean of the housekeeping gene GAPDH to control the variability in expression levels and calculated as 2^− [(C^_t_ ^of the^ *^gene^*^) – (C^_t_ ^of^ *^GAPDH^*^)]^, where C_t_ represents the threshold cycle for each transcript. All primers are listed in Table S5, Supporting Information.

**Immunoblotting (IB) analysis**

IB analysis was performed according to a standard protocol with the following antibody: anti-ABHD17C (1:200, Cat: ab151040, Abcam), anti-ALOX15B (1:200, Cat: ab23691, Abcam), anti-DCAF10 (1:1000, Cat: 12108-1-AP, Proteintech), ABHD17B(H00051104-B01P, Thermo), DHX9(1:5000, Cat: 17721-1-AP, Proteintech), G6PD(1:1000, Cat:25413-1-AP, Proteintech), BAG6(1:2000, Cat: 26417-1-AP, Proteintech), DNAJB1(1:5000, Cat: 13174-1-AP, Proteintech), anti-PEBP1 (1:1000, SC-28837, Santa Cruz), anti- HA tag (1:5000, Cat: 66006-2-Ig, Proteintech), anti-Flag tag (1:5000, Cat: 66008-4-Ig, Proteintech), anti-Myc tag (1:2000, Cat: 16286-1-AP, Proteintech), anti-PSMD2(26S) antibody (1:10000, Cat:67770-1-Ig, Proteintech), anti-Integrin β1 antibody (1:5000, Cat: 12594-1-AP, Proteintech), anti-ERK1 (1:1,000; Abcam, ab32537)，anti-ERK2 (1:1,000; Abcam, ab32081)，anti-ERK1/2 (1:4000, Cat: 11257-1-AP, Proteintech), anti-p-ERK1/2 (1:1000, Cat: 9101,CST), anti-MEK (1:1000, Cat: 9122,CST), anti-p-MEK1/2 (1:1000, Cat: 9154,CST), anti-AKT1 (1:800, Cat:10176-2-AP, Proteintech), anti-FSP1 (1:1,000; Cat: ab155326, Abcam), anti-GPX4 (1:3,000, Cat: ab125066, Abcam), anti-DHODH (1:1,000, Cat: ab174288, Abcam), anti-GCH1 (1:1,000, Cat: ab307507, Abcam), anti-ACSL4 (1:10,000, Cat: ab155282, Abcam), anti-LPCAT3 (1:1,000, Cat: ab239585, Abcam), anti-GAPDH (1:5000, Cat: 60004-1-Ig, Proteintech). Uncropped IB images were provided in Supporting Information.

**Immunoprecipitation (IP)**

The indicated cells were lysed in lysis buffer (25 mM HEPES [pH 7.4], 1% NP-40, 1 mM EDTA, 150 mM NaCl, 2% glycerol, 1 mM PMSF) and maintained on ice for 30 minutes. After centrifugation at 15 000 RCF(g) for 10 min at 4°C, the supernatant was subjected to indicated antibody-conjugated G-agarose beads and rotated overnight at 4°C. The immunoprecipitates were washed six times with wash buffer (25 mM HEPES [pH 7.4], 0.5% NP-40, 1 mM EDTA, 150 mM NaCl, 2% glycerol, 1 mM PMSF) and samples were ready for IB or re-immunoprecipitation.

***In vivo* ubiquitination assays**

The indicated cells were co-transfected with either Flag-ALOX15B(wt), or Flag-ALOX15B(C106A), or Myc-ABHD17C(wt), or Myc-ABHD17C(S266A), or Myc-ABHD17C(S266E) plus HA-Ub-K6, K11, K27, K29, K33, K48, or K63 plasmids. After 48 h of transfection, IP was performed with anti-ALOX15B or anti-Flag antibody, followed by immunoblotting with indicated antibodies.

**Acyl-biotin exchange (ABE) assay**

ABE assay was performed according to the published procedure with slight modifications^[1, 2]^. In brief, cells were lysed in lysis buffer (50 mM Tris-HCl, 150 mM NaCl, 1 mM MgCl_2_, 1% NP-40, 10% glycerol, phosphatase inhibitor and protease inhibitor) for 1 h at 4 °C then centrifuged at 12000 RCF(g) /4 °C for 15 min. The cell lysates were incubated with solubilization buffer (1.7% Triton X-100, 5 mM EDTA, 20 mM methyl methanethiosulfonate (MMTS, Thermo Fisher Scientific), 50 mM Tris (pH 8.0), 4% SDS, and protease/phosphatase inhibitor cocktail) at 40 °C for 3 h, and endogenous ALOX15B or Flag- ALOX15B was purified by specific antibodies and beads (shown as “input” in figure 4). Then, the beads were washed five times with lysis buffer with pH 7.5 and then three times with lysis buffer with pH 7.2. Then, the beads were incubated with 1 M hydroxylamine (HAM, Sigma-Aldrich), protease inhibitor and phosphatase inhibitor containing lysis buffer with pH 7.2 at room temperature for 1 h. Each sample was divided into two parts, one omitting the HAM cleavage step (-HAM) and one including the HAM step (+HAM). After being washed four times with lysis buffer with pH 7.2 and once with lysis buffer with pH 6.2, beads were treated with a thiol-reactive biotin molecule, HPDP-Biotin (Thermo Fisher Scientific), in lysis buffer with pH 6.2 at 4 °C for 1 h. Gently wash all samples once in lysis buffer with pH 6.2 and three times in lysis buffer with pH 7.5. The immunoprecipitated samples were analyzed by immunoblot analysis using anti-ALOX15B antibody or anti-Flag antibody.

**Far-western analysis**

Far-western analysis was performed by using the proteins immunoprecipitated by anti-flag antibody, which were futher separated by SDS-PAGE and transferred onto a PVDF membrane. Membranes were then preincubated in 10% skimmed milk for 1h at 4 °C. As indicated, human recombinant DCAF10 protein (Cat CSB-YP719034HU, Cusabio), human recombinant ABHD17C protein (Cat: R16131h, EIAab) or human recombinant ERK1 protein (Cat: ab105904, Abcam) was added at 5 μg/ml and incubated at 4 °C for 18h. After extensive washing six times with TBST, the membrane was subjected to IB analysis by indicated antibody.

**Surface plasmon resonance (SPR) analysis**

The interaction of recombinant ALOX15B protein (Cat: HY-P74437, MCE) with DCAF10 (Cat: Ag14884, Proteintech), and immunoprecipitated-ABHD17C protein and MPD (Cat: HY-N0863, Proteintech) were detected using a BIAcore T200 instrument (GE Healthcare, UK) following the manufacturer’s instructions. SPR equilibrium binding data, consisting of Req values from several concentration series, were analyzed by fitting a simple 1:1 binding to yield Rmax and Kd values using BI Acore T200 Evaluation software.

**Immunofluorescence (IF) staining**

IF staining was carried out on cell chamber slide cultures (Thermo Fisher Scientific) and followed by the antibodies: anti-ALOX15B (1:100, Cat: ab23691, Abcam). The secondary antibody was anti-Rabbit Alexa Fluor 594 (1:500, Cat: 8889S, Cell Signaling). Then cells were mounted with antifade Mountant with DAPI (Thermo Fisher Scientific). Cells were imaged by confocal microscopy (Carl Zeiss, Jena, Germany), and images acquired for the experiments were processed and analyzed in ImageJ v1.48.

**Lipid peroxidation was assessed by BODIPY™ 581/591 C11 staining**

The day before the experiments, the indicated cells (5×10^5^) /well were seeded into a 24-well plate contained one 12 mm^2^ coverslip in each well. At the end of the treatment, the treatment medium was removed and cells were washed once with HBSS. Cells were then labeled in 1 mL HBSS containing 5 μM BODIPY 581/591 C11 (Cat: D3861 Thermo Fisher Scientific) and incubated at 37 °C for 20 min. Subsequently, cells were washed twice with HBBS. Then, cells were imaged by confocal microscopy (Carl Zeiss, Jena, Germany), and images acquired for the experiments were processed and analyzed in ImageJ v1.48.

**Lactate dehydrogenase (LDH) release assay**

The extent of cell death was quantitatively assessed by measuring the release of LDH into the culture media using the cytotoxicity LDH assay kit (Dojindo) according to the manufacturer’s protocol. In brief, cells were seeded onto 96-well plates at a density of 5×10^3^/well. The next day, cells were treated with the compounds indicated in the relevant figure captions. Subsequently, the culture medium from each condition was transferred to a 96-well plate for the quantification of LDH. The absorbance of each sample was read at 490 nm using a microplate reader (SpectraMax i3X, Molecular Devices).

**Cell viability assay**

Cells were measured using a Cell Counting Kit-8 (CCK-8, Cat: CK04, Dojindo). In brief, cells were seeded onto 96-well plates at a density of 5×10^3^/well. The next day, cells were treated with the indicated compounds. Subsequently, cells were exposed to 10 μl CCK-8 reagent (100 μl medium per well) for 1 h at 37 °C, 5% CO_2_ in an incubator. The absorbance at a wavelength of 450 nm was determined using a Spectro fluorimeter (SpectraMax i3X, Molecular Devices).

**Immunohistochemistry (IHC)**

IHC analysis was performed to determine altered protein expression in paraffin-embedded PDAC tissues with anti-ABHD17C (1:100, Cat: ab151040, Abcam), anti-ALOX15B (1:200, Cat: sc-271290, Santa Cruz) and anti-MDA (1:100, Cat: JAI-MMD-030N, Adipogenic), anti-4-HNE (1:200, Cat: ab46545, Abcam), anti-Cleaved caspase 3 (1:400, Cat: 9661S, Cell signaling technology) antibodies overnight at 4 °C. The degree of immunostaining of formalin-fixed, paraffin-embedded sections was reviewed and scored separately by two independent pathologists uninformed of the histopathological features and patient data of the samples. The scores were determined by combining the proportion of positively stained tumor cells and the intensity of staining. The scores given by the two independent pathologists were combined into a mean score for further comparative evaluation. Tumor cell proportions were scored as follows: 0, no positive tumor cells; 1, < 10% positive tumor cells; 2, 10–35% positive tumor cells; 3, 35–75% positive tumor cells; 4, > 75% positive tumor cells. Staining intensity was graded according to the following standard: 1, no staining; 2, weak staining (light yellow); 3, moderate staining (yellow-brown); 4, strong staining (brown). The staining index (SI) was calculated as the product of the staining intensity score and the proportion of positive tumor cells. Using this method of assessment, we evaluated protein expression in benign esophageal epithelial and malignant lesions by determining the SI, with possible scores of 0, 1, 2, 3, 4, 6, 8, 9, 12, and 16. Samples with a SI ≥ 8 were determined as high expression and samples with a SI < 8 were determined as low expression. Cutoff values were determined based on a measure of heterogeneity using the log-rank test concerning overall survival.

**RNA-seq analysis**

Total RNA was isolated and purified using TRIzol reagent (Life Technologies) in accordance with the manufacturer's guidelines. The concentration and integrity of the extracted RNA were assessed using the Bioanalyzer 2100 system (Agilent Technologies). Strand-specific RNA-seq libraries were then prepared for high-throughput sequencing on an Illumina platform, following the recommended protocol provided by the manufacturer. The RNA-seq reads were aligned to the human reference genome (GRCh37, Ensembl, Illumina) using HISAT2 (v2.2.9). Gene-level read counts were obtained with htseq-count (v0.11.2). Differential gene expression analysis was performed using the DEGseq package (v1.36.1), with statistical significance assessed by Student’s t-test for P value calculation.

**Transmission electron microscopy**

TEM analysis was performed by the High-Resolution Electron microscopy facility at the instrument center of Zhongshan School of Medical, Sun Yat-Sen University. Samples were fixed with a solution containing 3% glutaraldehyde and 2% paraformaldehyde in 0.1 M cacodylate buffer (pH 7.3), then washed in 0.1 M sodium cacodylate buffer and treated with 0.1% Millipore-filtered cacodylate-buffered tannic acid, postfixed with 1% buffered osmium and stained en bloc with 1% Millipore-filtered uranyl acetate. The samples were dehydrated in increasing concentrations of ethanol, infiltrated, and embedded in LX-112 medium. The samples were polymerized in a 60 °C oven for approximately 3 days. Ultrathin sections were cut in a Leica Ultracut microtome (Leica UC6), stained with uranyl acetate and lead citrate in a Leica EM Stainer, and examined in a Tecnai G2 SpiritTwin transmission electron microscope at an accelerating voltage of 80 kV. Digital images were obtained using the GATAN 832.10W System.

**Arachidonic acid metabolism assay**

Metabolomic analysis involved the LC–MS/MS analysis of metabolites, as previously described^[3]^. Briefly, the indicated cells or tumors was spiked with 1 μL of each internal standard mixture (5 ng of each of the following internal standards: LTB4-d4, PGE2-d4, 6-keto-PGF1a-d4, 11, 12-DHET-d11, 20-HETE-d6, 8, 9-EET-d11, 5-HETE-d8, ARA-d8, EPA-d5, DHA-d5, and 9-HODE-d9). Arachidonic acid metabolites were successively extracted using methanol and ethyl acetate (containing 0.01 mol /L butylated hydroxytoluene). The extraction process was performed under weak-light conditions. The extracted samples were evaporated to dryness using a gentle stream of nitrogen gas. The samples were reconstituted in 30% acetonitrile and then filtered. We used centrifuge tubes with low sorption properties during the extraction process. Chromatographic separations were conducted using ultra performance liquid chromatography (UPLC) BEH C18 columns (1.7 μm, 100 × 2.1 mm i.d.). Target profiling of arachidonic acid metabolites was performed using a 5500 QTRAP Hybrid Triple Quadruple–Linear Ion Trap Mass Spectrometer (AB Sciex, Framingham, MA, USA) equipped with a turbo ion-spray electrospray ionization source. MetaboAnalyst 3.0 (https://www.metaboanalyst.ca/) was used for subsequent data analysis. Missing values were input with half of the minimum positive value, and the data were log transformed and auto-scaled before analysis.

**References**

[1] L. Wang, J. Cai, X. Zhao, L. Ma, P. Zeng, L. Zhou, Y. Liu, S. Yang, Z. Cai, S. Zhang, L. Zhou, J. Yang, T. Liu, S. Jin,J. Cui, *Mol Cell* **2023**, *83*, 281-297.e10.

[2] L. Zhou, X. He, L. Wang, P. Wei, Z. Cai, S. Zhang, S. Jin, H. Zeng,J. Cui, *Cell Death Differ* **2022**, *29*, 1541-1551.

[3] W. Cai, L. Liu, X. Shi, Y. Liu, J. Wang, X. Fang, Z. Chen, D. Ai, Y. Zhu,X. Zhang, *Circulation* **2023**, *147*, 1444-1460.

**Supplementary Tables**

**Table S1. Correlation between ALOX15B expression and clinicopathologic characteristics of KRAS^mut^ PDAC patients.**

| **Characteristics** | **No. of cases** | **ALOX15B expression level** | | |
| --- | --- | --- | --- | --- |
|  |  | **Low** | **High** | **p-value** |
| **Total cases** | 95 | 60 | 35 |  |
| **Gender** |  |  |  |  |
| **Male** | 55 | 33 | 22 | 0.454 |
| **Female** | 40 | 27 | 13 |  |
| **Age** |  |  |  |  |
| **≤60** | 38 | 23 | 15 | 0.664 |
| **>60** | 57 | 37 | 20 |  |
| **Differentiation** |  |  |  |  |
| **Poor** | 20 | 12 | 8 |  |
| **Moderate** | 54 | 30 | 24 | 0.742 |
| **Well** | 31 | 18 | 3 |  |
| **T stage** |  |  |  |  |
| **T1-2** | 20 | 10 | 10 | 0.170 |
| **T3-4** | 75 | 50 | 25 |  |
| **Lymphatic metastasis** |  |  |  |  |
| **Negative** | 45 | 26 | 19 | 0.302 |
| **Positive** | 50 | 34 | 16 |  |
| **TNM stage** |  |  |  |  |
| **Stage Ⅰ** | 23 | 10 | 13 | 0.025 |
| **Stage Ⅱ** | 48 | 40 | 8 |  |
| **Stage Ⅲ** | 24 | 10 | 14 |  |
| **Vital Status** |  |  |  |  |
| **Alive** | 29 | 12 | 17 | 0.004 |
| **Death** | 66 | 48 | 18 |  |
| **Recurrence** |  |  |  |  |
| **NO** | 23 | 10 | 13 | 0.025 |
| **YES** | 72 | 50 | 22 |  |

**Table S2. Univariate and multivariate analysis of different prognostic parameters in KRAS^mut^ PDAC patients by Cox-regression analysis**

|  | **Univariate analysis** | | **Multivariate analysis** | |
| --- | --- | --- | --- | --- |
|  | **P** | Hazard ratio  (95% CI) | P | Hazard ratio  (95% CI) |
| **Age** | 0.164 | 1.460  (0.857-2.487) |  |  |
| **Gender** | 0.08 | 0.640  (0.388-1.054) |  |  |
| **Differentiation** | 0.084 | 1.688  (0.932-3.058) |  |  |
| **T stage** | 0.064 | 0.566  (0.310-1.034) |  |  |
| **Lymphatic metastasis** | 0.243 | 0.749  (0.461-1.217) |  |  |
| **TNM stage** | 0.032 | 0.543  (0.311-0.949) | < 0.001 | 0.203  (0.094-0.439) |
| **Recurrence** | < 0.001 | 5.62  (2.690-11.742) | < 0.001 | 5.095  (2.365-10.977) |
| **ALOX15B expression** | 0.002 | 0.428  (0.248-0.739) | < 0.001 | 0.237  (0.112-0.502) |

**Table S3. Correlation between ABHD17C expression and**

**clinicopathologic characteristics of KRAS^mut^-PDAC patients.**

| **Characteristics** | **No. of cases** | **ABHD17C expression level** | | |
| --- | --- | --- | --- | --- |
|  |  | **Low** | **High** | **p-value** |
| **Total cases** | 95 | 31 | 64 |  |
| **Gender** |  |  |  |  |
| **Male** | 55 | 18 | 37 | 0.981 |
| **Female** | 40 | 13 | 27 |  |
| **Age** |  |  |  |  |
| **≤60** | 38 | 11 | 27 | 0.532 |
| **>60** | 57 | 20 | 37 |  |
| **Differentiation** |  |  |  |  |
| **Poor** | 20 | 8 | 12 | 0.429 |
| **Moderate** | 54 | 15 | 39 |  |
| **Well** | 31 | 8 | 13 |  |
| **T stage** |  |  |  |  |
| **T1-2** | 20 | 10 | 10 | 0.062 |
| **T3-4** | 75 | 21 | 54 |  |
| **Lymphatic metastasis** |  |  |  |  |
| **Negative** | 45 | 19 | 26 | 0.243 |
| **Positive** | 50 | 12 | 38 |  |
| **TNM stage** |  |  |  |  |
| **Stage Ⅰ** | 23 | 12 | 11 | 0.022 |
| **Stage Ⅱ** | 48 | 16 | 32 |  |
| **Stage Ⅲ** | 24 | 3 | 21 |  |
| **Vital Status** |  |  |  |  |
| **Alive** | 29 | 17 | 12 | < 0.001 |
| **Death** | 66 | 14 | 52 |  |
| **Recurrence** |  |  |  |  |
| **NO** | 23 | 12 | 11 | 0.022 |
| **YES** | 72 | 19 | 53 |  |

**Table S4. Univariate and multivariate analysis of different prognostic parameters in KRAS^mut^ PDAC patients by Cox-regression analysis**

|  | **Univariate analysis** | | **Multivariate analysis** | |
| --- | --- | --- | --- | --- |
|  | **P** | Hazard ratio  (95% CI) | P | Hazard ratio  (95% CI) |
| **Age** | 0.164 | 1.460  (0.857-2.487) |  |  |
| **Gender** | 0.08 | 0.640  (0.388-1.054) |  |  |
| **Differentiation** | 0.084 | 1.688  (0.932-3.058) |  |  |
| **T stage** | 0.064 | 0.566  (0.310-1.034) |  |  |
| **Lymphatic metastasis** | 0.243 | 0.749  (0.461-1.217) |  |  |
| **TNM stage** | 0.032 | 0.543  (0.311-0.949) | < 0.001 | 0.168  (0.078-0.362) |
| **Recurrence** | < 0.001 | 6.472  (2.984-14.034) | < 0.001 | 5.73  (2.515-13.055) |
| **ALOX15B expression** | < 0.001 | 3.036  (1.673-5.511) | < 0.001 | 6.173  (2.758-13.815) |

**Table S5. Primers and Oligonucleotides used in this study**

| **Primer used for qPCR** | |
| --- | --- |
| ALOX15B-up | CAGTGGAAGGCTTACAACCCA |
| ALOX15B-dn | CAGTGCTCAAATGCGTGCT |
| ABHD17C-up | CTACTCGGGATACGGCGTCA |
| ABHD17C-dn  KRAS-up  KRAS-dn | AGAGGATAATGTTCTCGGGACTC  ACAGAGAGTGGAGGATGCTTT  TTTCACACAGCCAGGAGTCTT |
| **siRNA sequences** |  |
| si-DCAF10#1 | CUCUACGACUGACUCAUUA |
| si-DCAF10#2 | CGAATGAGGTTAACACCAGAT |
| si-ABHD17C#1 | GAGGAUGAGGUCAUCGAUUUC |
| si-ABHD17C#2 | UAUGAAUGCGCAGCGGUAAUU |
| si-DHX9#1 | GAAGGAUUACUACUCAAGAAA |
| si-DHX9#2 | UCGAGGAAUCAGUCAUGUAAU |
| si-ABHD17B#1 | GCTGCTGTTATTCTTCATT |
| si-ABHD17B#2 | GTGGAACTTTATGGACAGTAT |
| si-G6PD#1 | CAACAGAUACAAGAACGUGAA |
| si-G6PD#2 | CCCUATAUUUAUGGCAGCCGA |
| si-BAG6#1 | CACCGCGGTACTGGTACTATCATT |
| si-BAG6#2 | AAACCTGTCAGCATCCCATCTGAC |
| si-DNAJB1 #1 | GCAGUUCAGGUGAUACAUUUA |
| si-DNAJB1 #2 | CCCUGAAGUGACUGAGACAAU |
| si-PEBP1 #1 | CGAGCAGGACAGGCCGCTAAA |
| si-PEBP1 #2 | GTGGTCAACATGAAGGGCAAT |
| **shRNA target sequences** |  |
| ABHD17C-sh1 | CATCAACTGTAACCATATAAA |
| ABHD17C-sh2 | GCGTGAGTCCCGAGAACATTA |
| DCAF10-sh1 | GGTGAAGAACATCGAATATGA |
| DCAF10-sh2 | GGGTTACATCAAAGAACTTTG |

**Source Data**


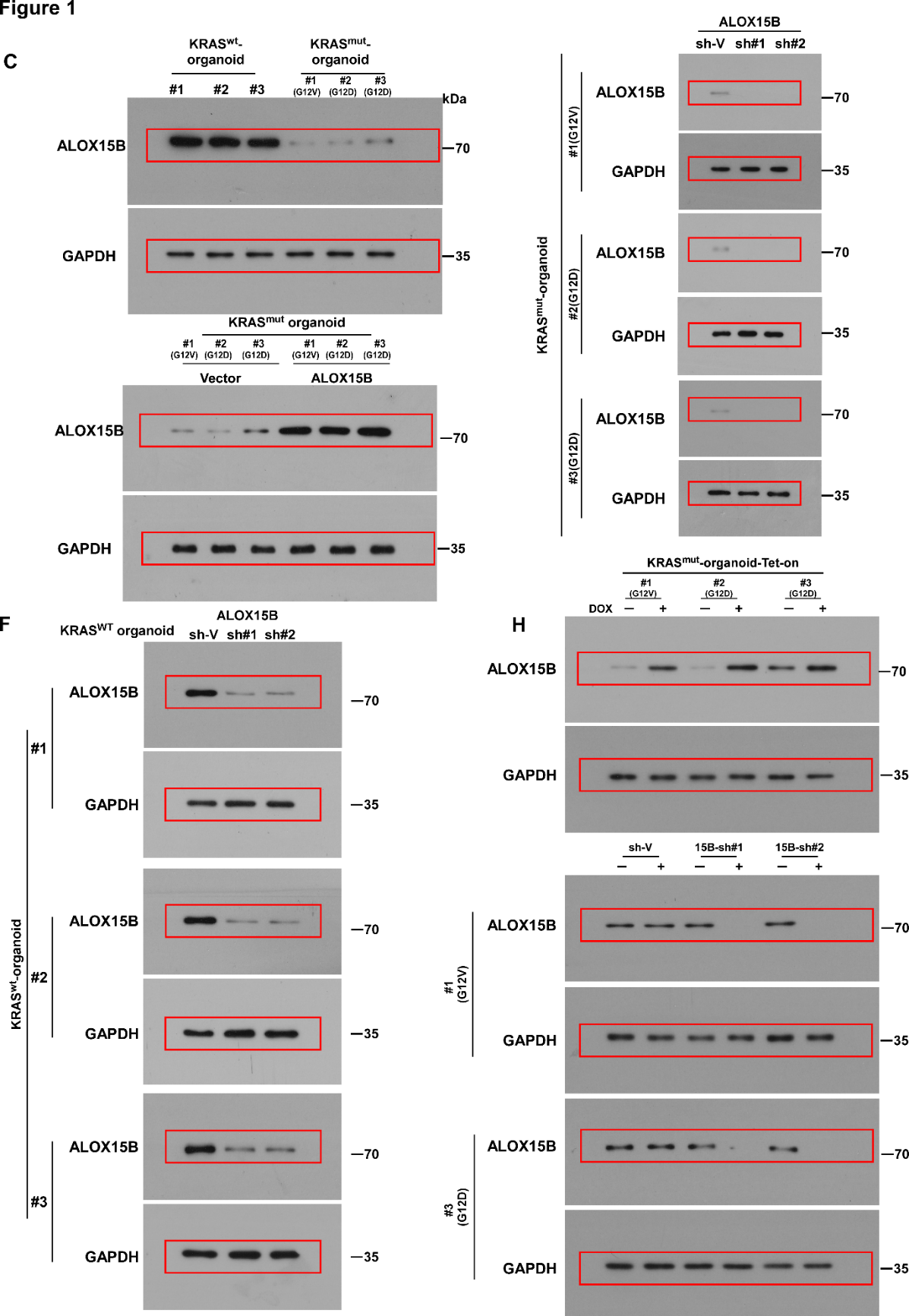


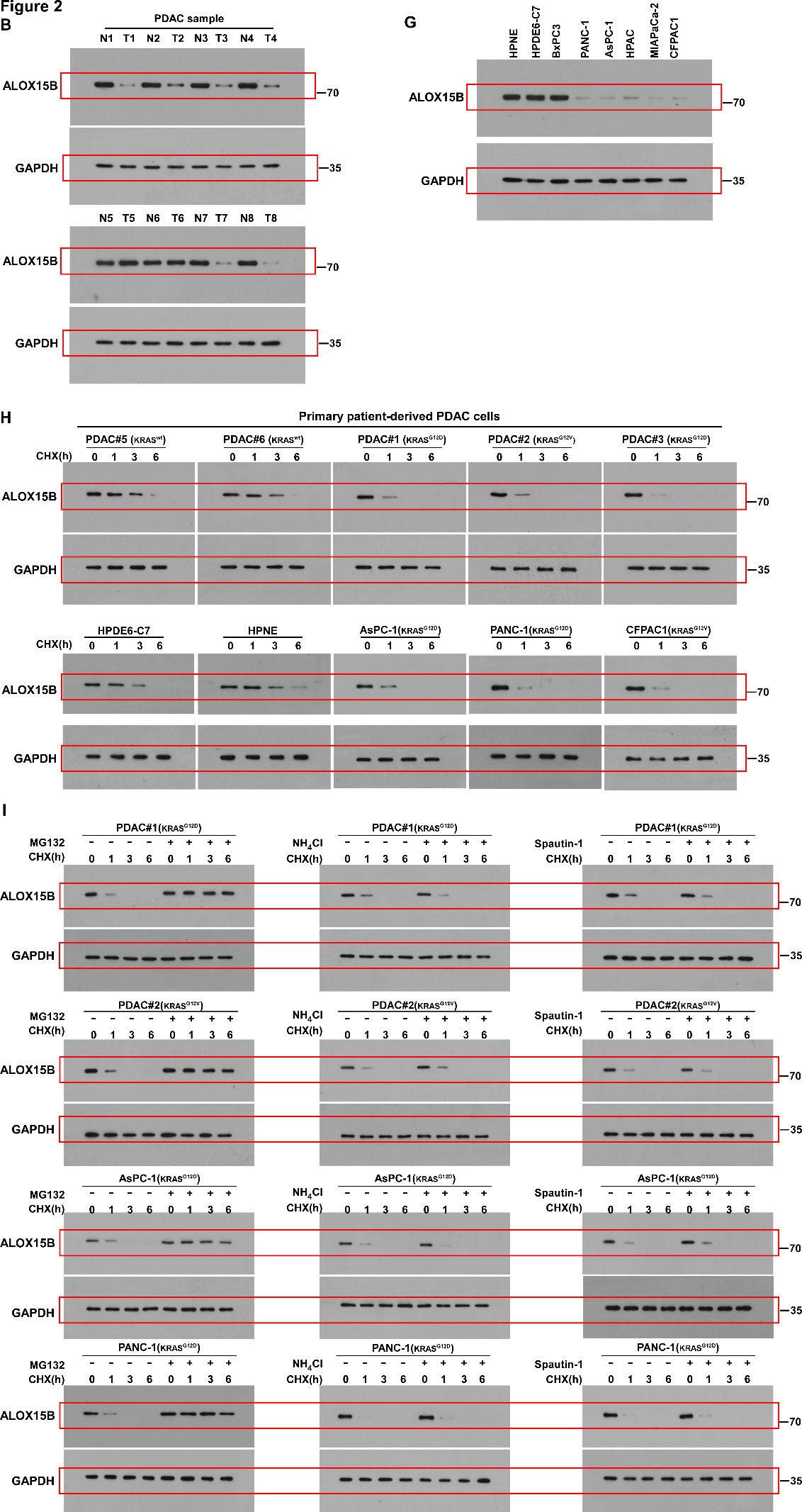


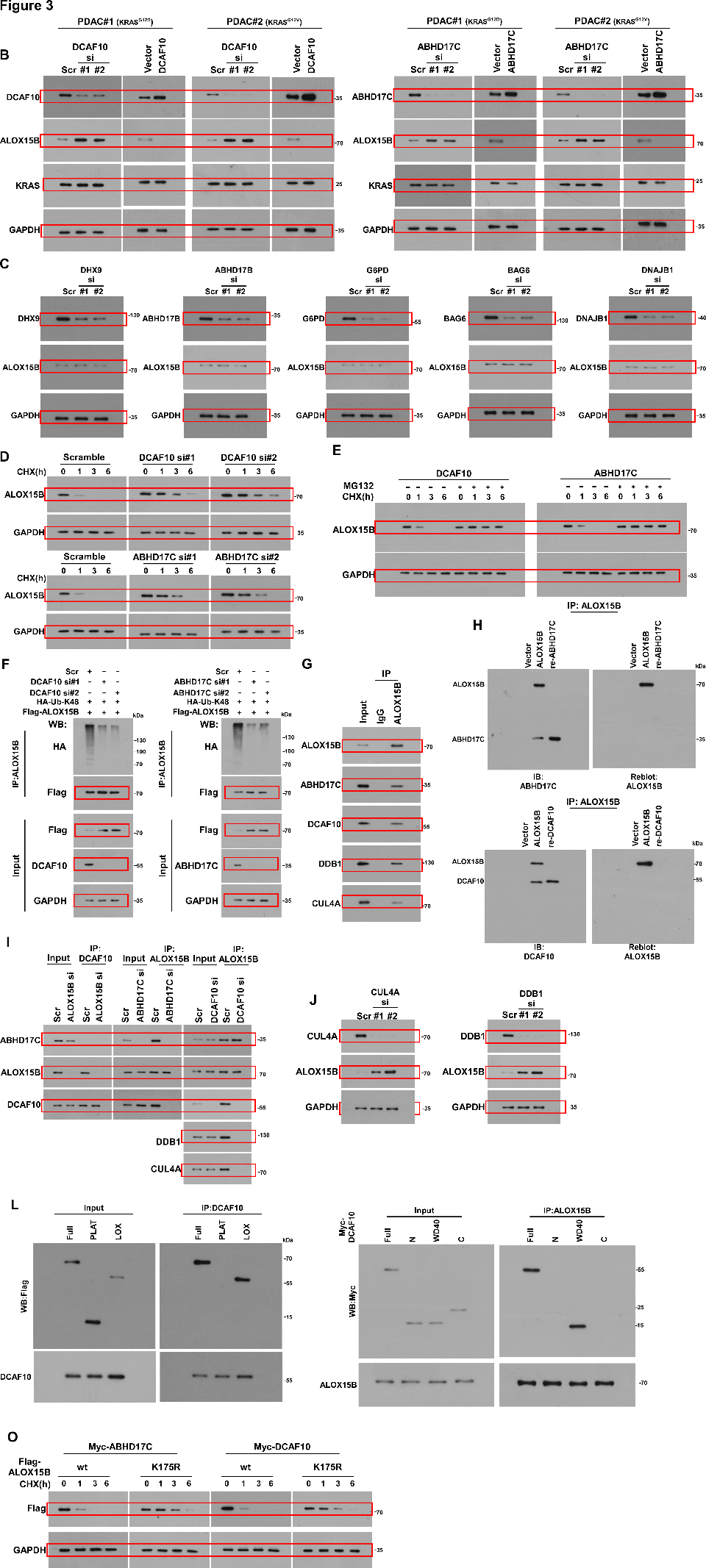


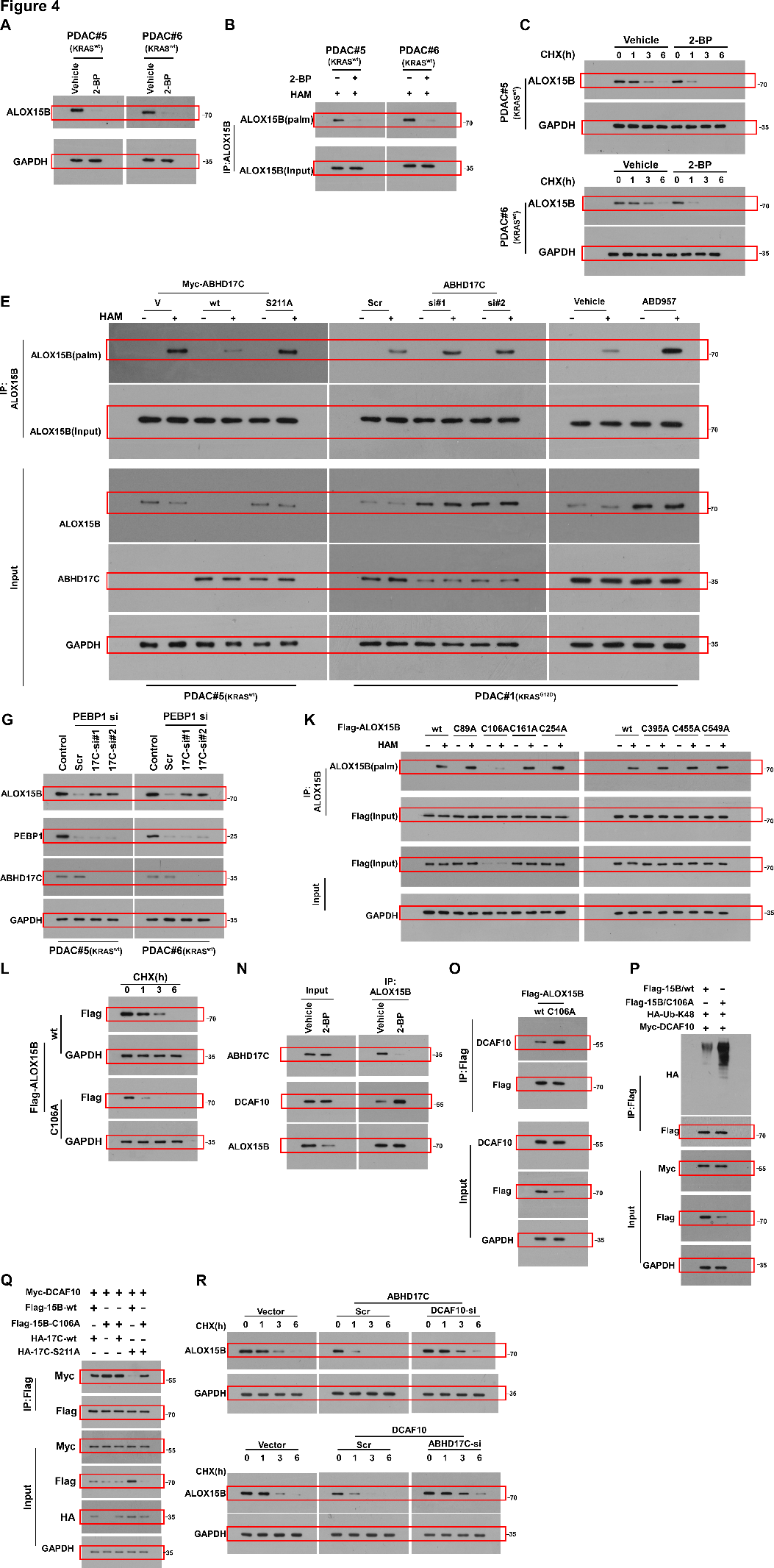


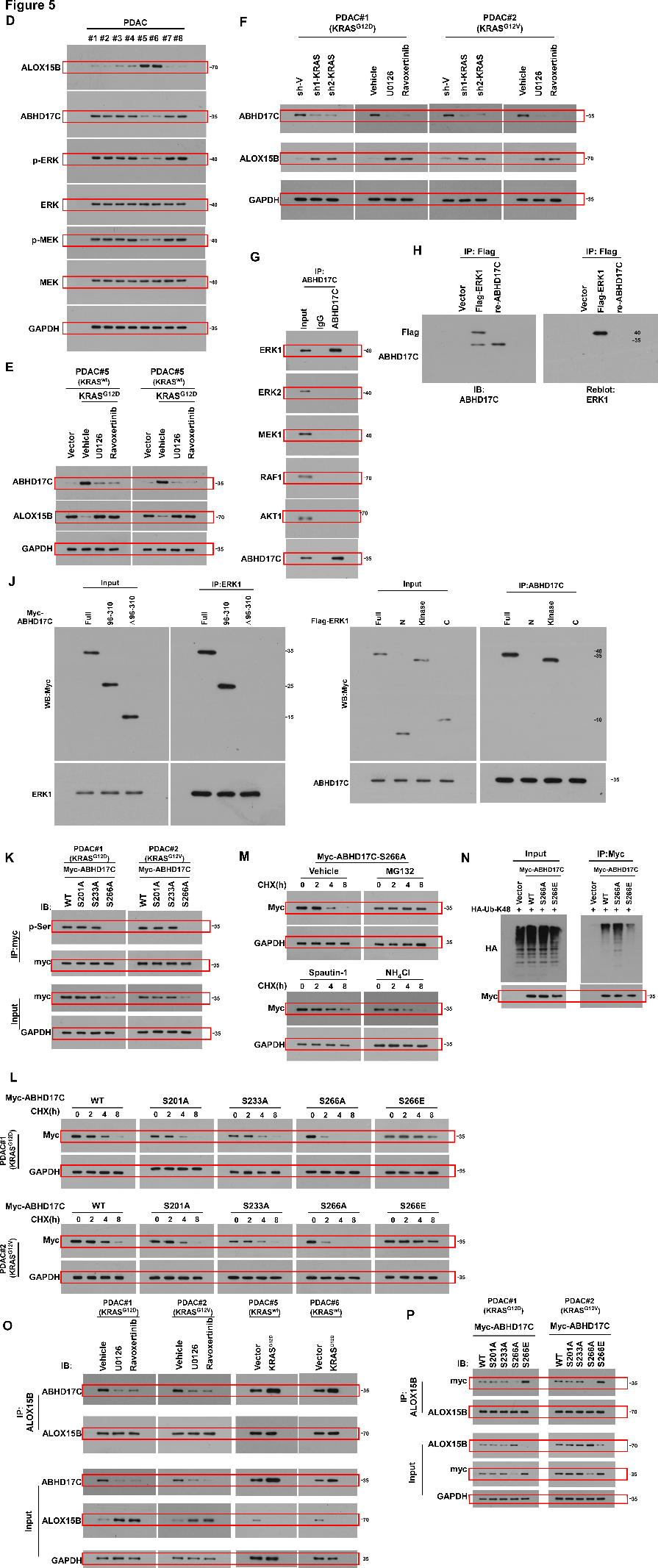


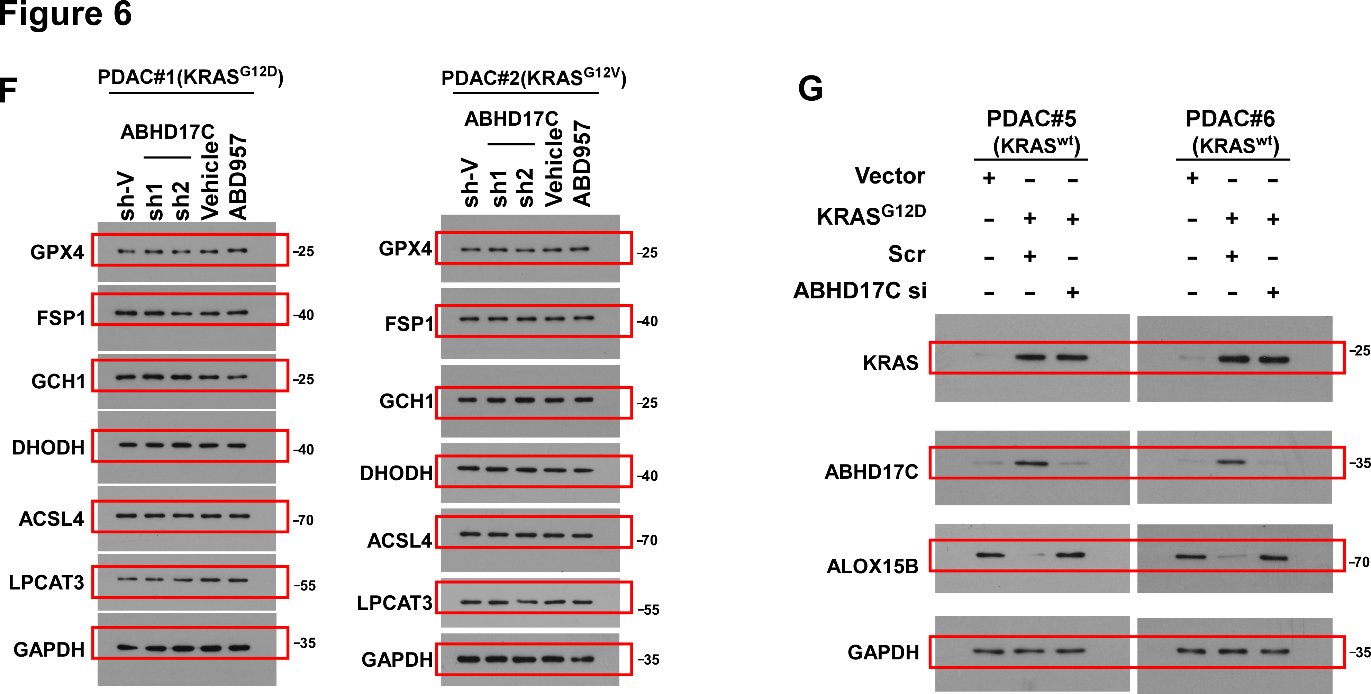


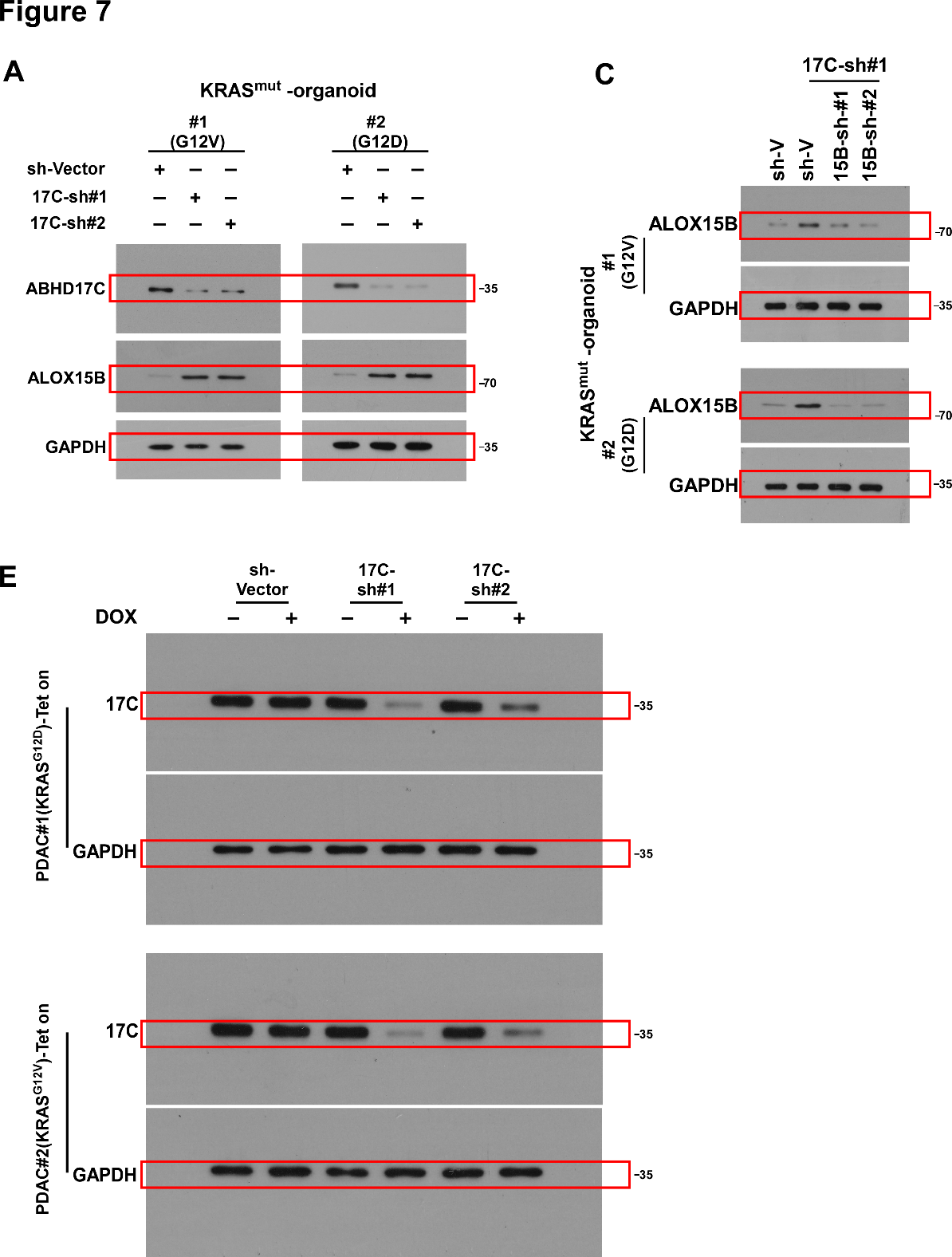


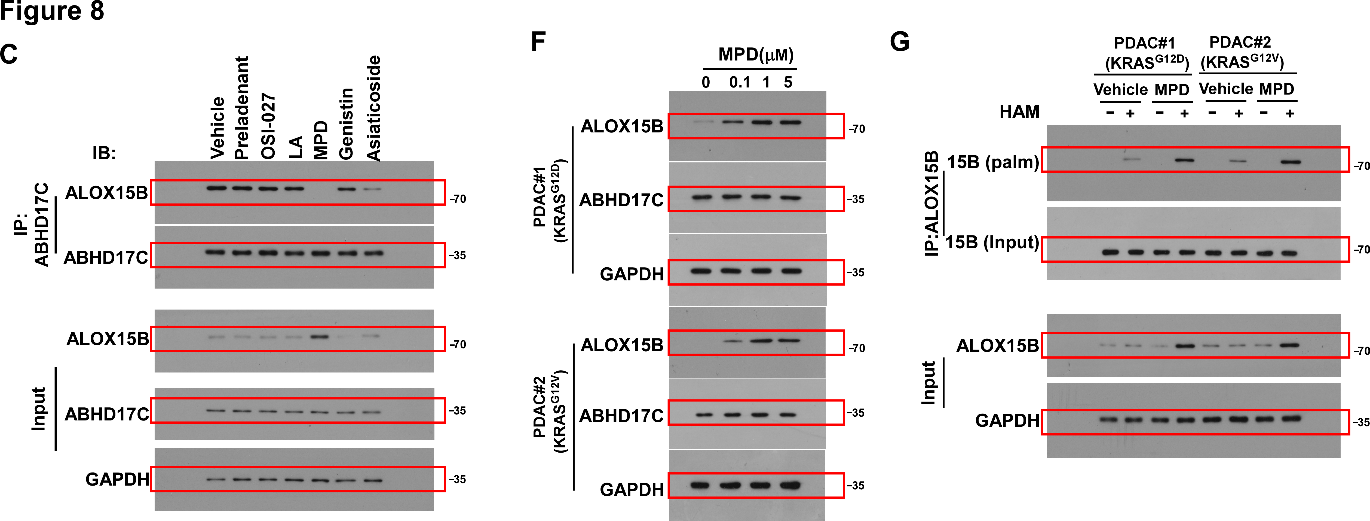


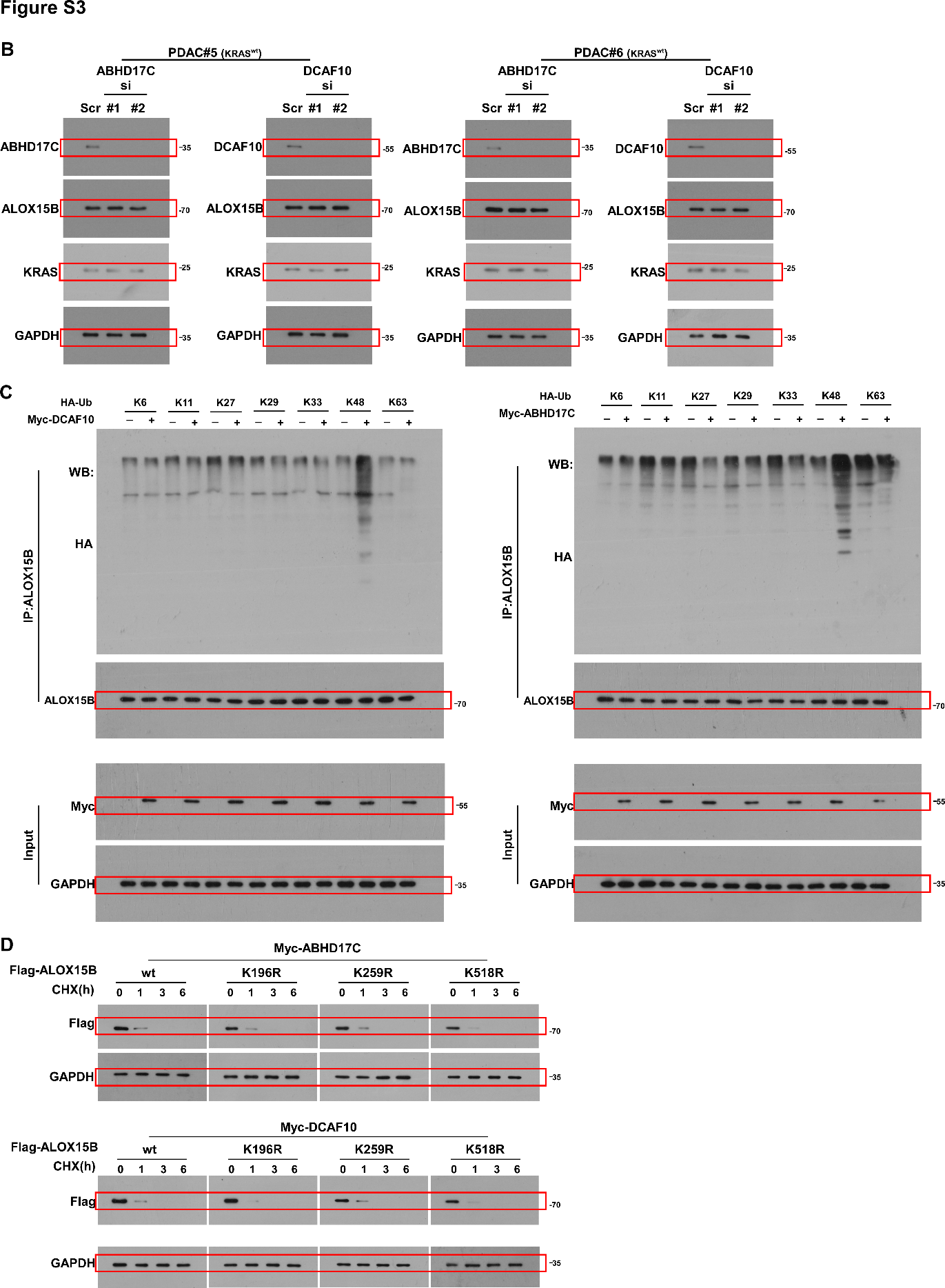


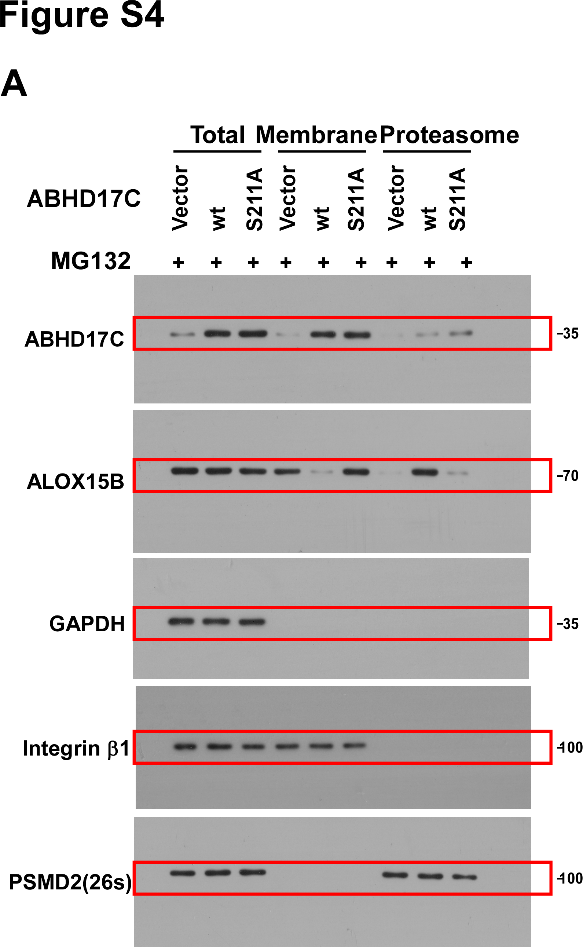


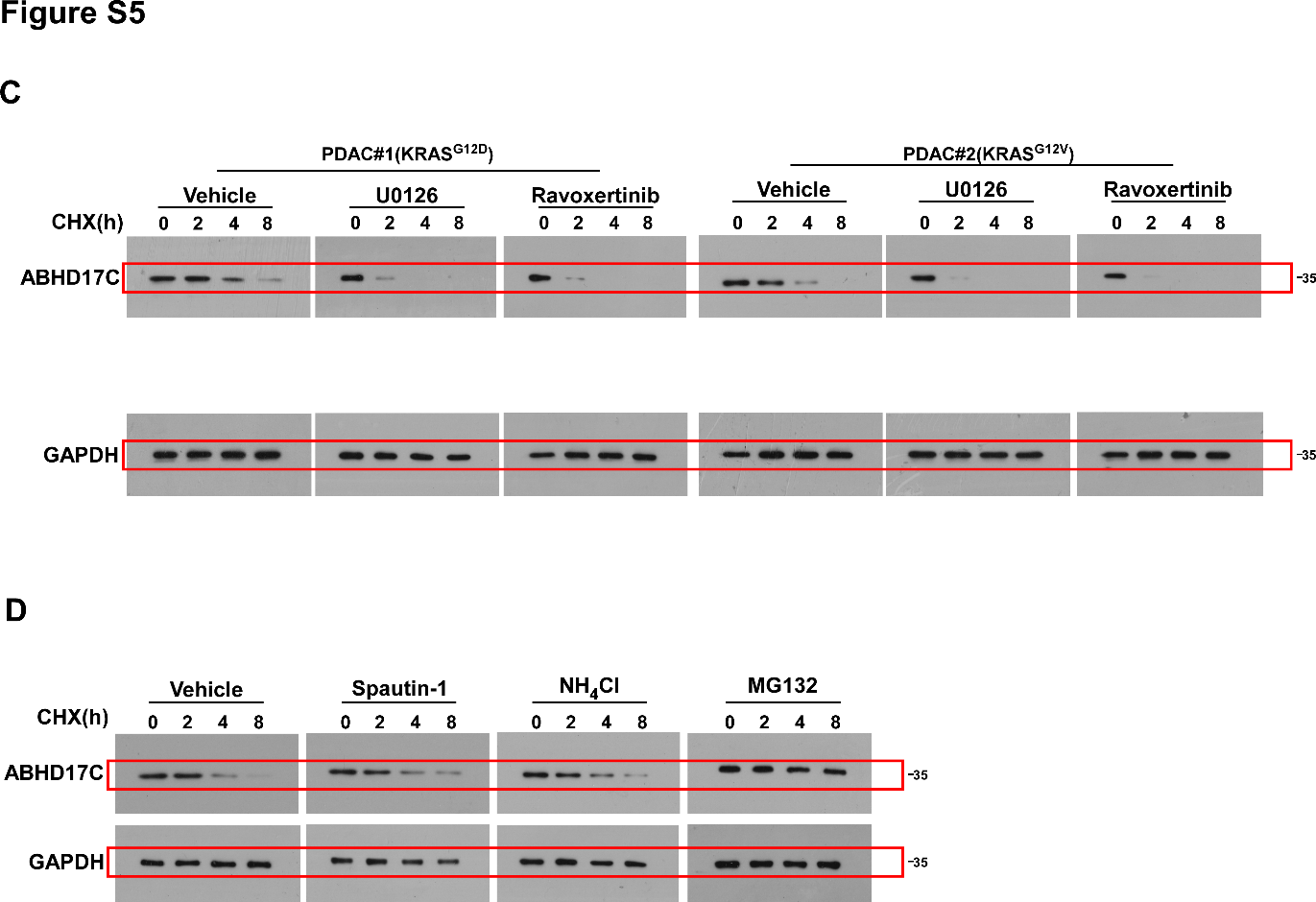


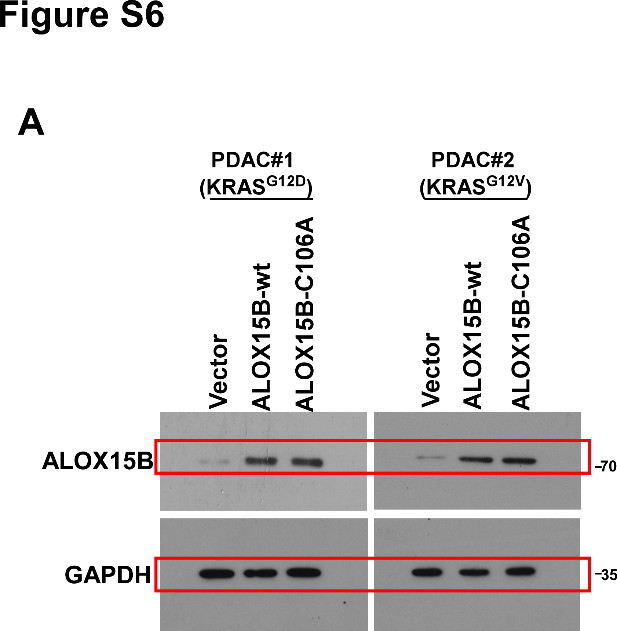


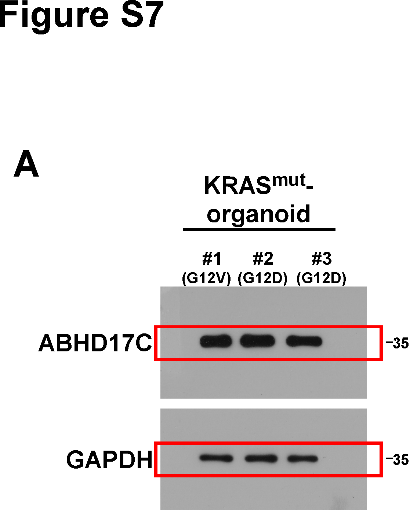


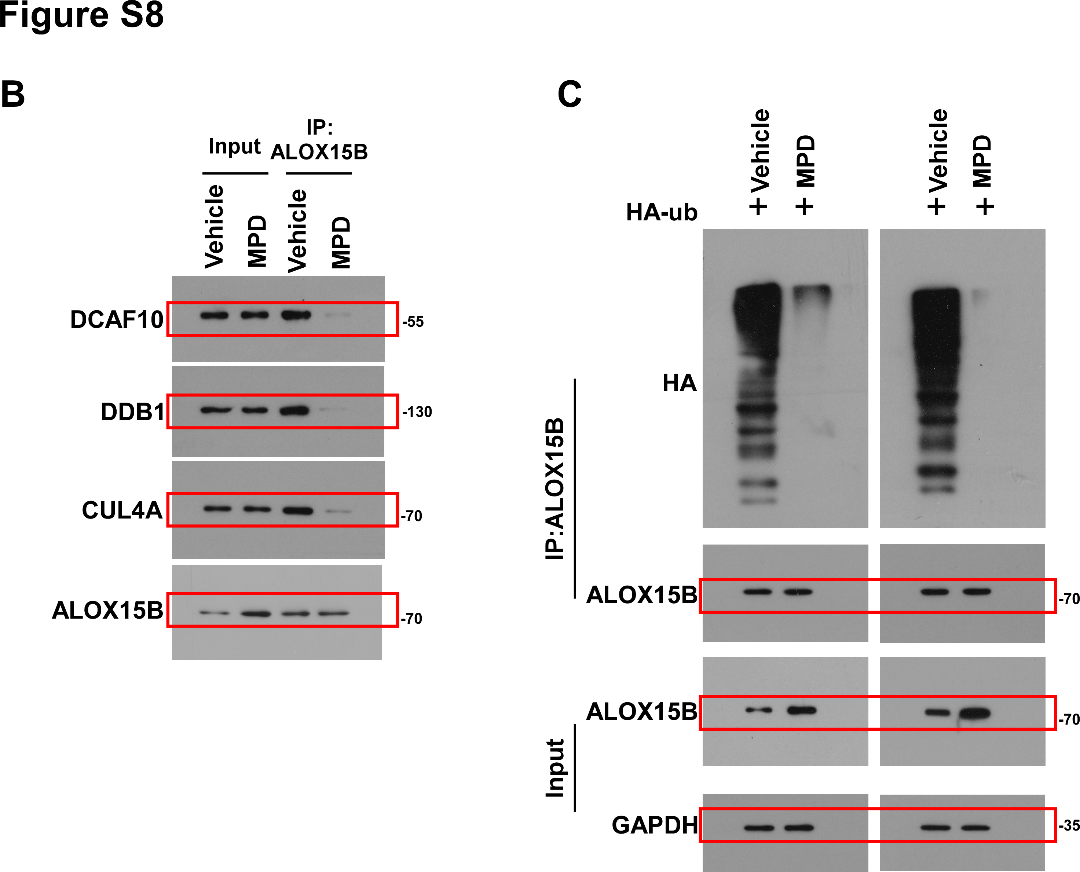

Supplement: Supplementary file 1 — Supporting Information [file ADVS-12-e04470-s001.docx]
